# Fluorescent mannosides serve as acceptor substrates for glycosyltransferase and sugar-1-phosphate transferase activities in *Euglena gracilis* membranes

**DOI:** 10.1016/j.carres.2016.11.017

**Published:** 2017-01-13

**Authors:** Irina M. Ivanova, Sergey A. Nepogodiev, Gerhard Saalbach, Ellis C. O'Neill, Michael D. Urbaniak, Michael A.J. Ferguson, Sudagar S. Gurcha, Gurdyal S. Besra, Robert A. Field

**Affiliations:** a Department of Biological Chemistry, John Innes Centre, Norwich Research Park, Norwich NR4 7UH, UK; b Biomedical and Life Sciences, Lancaster University, Furness Building, Lancaster LA1 4YG, UK; c College of Life Sciences, University of Dundee, Dundee DD1 5EH, Scotland, UK; d School of Biosciences, The University of Birmingham, Edgbaston, Birmingham B15 2TT, UK

**Keywords:** *Euglena gracilis*, Glycosyltransferases, Fluorescent glycans, *N*-acetylglucosamine-1-phosphate transferase, Enzyme assays

## Abstract

Synthetic hexynyl α-D-mannopyranoside and its α-1,6-linked disaccharide counterpart were fluorescently labelled through CuAAC click chemistry with 3-azido-7-hydroxycoumarin. The resulting triazolyl-coumarin adducts, which were amenable to analysis by TLC, HPLC and mass spectrometry, proved to be acceptor substrates for α-1,6-ManT activities in mycobacterial membranes, as well as α- and β-GalT activities in trypanosomal membranes, benchmarking the potential of the fluorescent acceptor approach against earlier radiochemical assays. Following on to explore the glycobiology of the benign protozoan alga *Euglena gracilis*, α-1,3- and α-1,2-ManT activities were detected in membrane preparations, along with GlcT, Glc-P-T and GlcNAc-P-T activities. These studies serve to demonstrate the potential of readily accessible fluorescent glycans as substrates for exploring carbohydrate active enzymes.

## Highlights

• Assays for the analysis of carbohydrate-active enzymes that rely upon fluorescent acceptor substrates are set out.
• New assays are validated by benchmarking against radiochemical work with known glycosyltransferase activities.
• The installation of a fluorophore on acceptor substrates was easily achieved through click chemistry.
• Fluorescence assays are used to discover GTs activities in *Euglena gracilis* microsomal membranes.

## Graphical abstract

**Figure:**
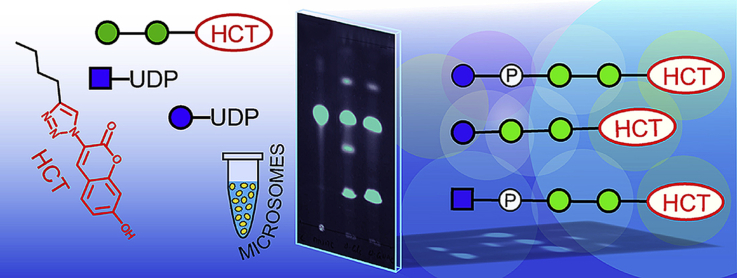

## 1 Introduction

The single-celled protozoan microalga *Euglena* has been the subject of scientific endeavour since it was first observed by van Leeuwenhoek [1]. This flagellated, unicellular microorganism mainly inhabits fresh water, although it is highly adaptable and it can survive under harsh environmental conditions (e.g. low pH, high salinity, high energy ionising radiation) [2]. *Euglena* exhibits both plant- and animal-like characteristics: it can live in light, sustaining itself autotrophically through photosynthesis; or it can survive heterotrophically in the dark, using nutrients from the environment. Despite being considered colloquially as a green alga, *Euglena* is classified in the phylum Euglenozoa [3], which also includes the human parasites *Trypanosoma brucei* [4] and *Leishmania major* [5]. The rich glycobiology of these parasites [6] prompted us to explore the capabilities of *E. gracilis* [7].

Reports of the characterisation of glycans from *Euglena* are limited to date, with most efforts focusing on its crystalline and granular storage β-1,3-glucan paramylon [8]. While *Euglena* do not possess a plant-like polysaccharide cell wall, undefined glycoproteins rich in xylose, mannose, glucose and galactose are present on the outer membrane of their flagella [9]. No other detailed information is currently available about glycoprotein, polysaccharide or GPI anchor structures from these organisms. The presence in *Euglena* of a typical eukaryote *N*-glycan precursor glycolipid [Glc_3_Man_9_GlcNAc_2_-P-P-dolichol] has been confirmed, which is in contrast to that found in the trypanosomes [Man_7-9_GlcNAc_2_-P-P-dolichol] [10]. We recently reported on the transcriptome of *Euglena gracilis* [11], which identified unexpected capacity for carbohydrate biochemistry. This organism possesses at least 126 glycoside hydrolases (GHs) and 229 glycosyltransferases (GTs) [7], [12], under the growth conditions employed for transcriptome analysis – numbers that rival the human CAZome, albeit with a different balance and repertoire of predicted activities. In order to experimentally validate assigned functions, suitable assays are needed. While a varied range of GT assays are available [13], the use of radiolabelled sugar nucleotides remains prevalent. To simplify the assays and avoid the necessity for radioactive material, we were drawn to explore more flexible fluorescence-based assays, employing fluorescently labelled acceptor glycans [14]. In recent work, we have explored capillary electrophoresis with laser-induced fluorescence (CE-LIF) detection as a high sensitivity, high resolution method for glycosyltransferase analyses, for instance [15]. Herein, we report the development of fluorescent coumarin derivatives, coupled with TLC or HPLC-MS analysis, for the straightforward detection and characterisation of *Euglena* carbohydrate-active enzyme activities.

## 2 Results and discussion

**Fig. 1:**
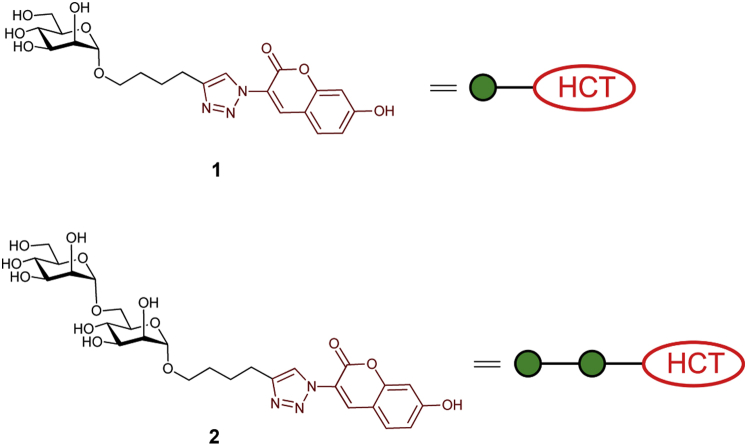
Structure of fluorescent acceptors α-Man-HCT (**1**) and α-Man-(1,6)-α-Man-HCT (**2**) and their schematic representations. HCT denotes fluorescent aglycone residue [(7-hydroxycoumarin-3-yl)-1H-1,2,3-triazlole-4-ylbutlyl)].

The initial goal of these studies was to develop acceptor glycan substrates that could be used to assess the expected eukaryotic *N*-linked glycan and GPI anchor pathway GTs that one might reasonably expect to find in *Euglena* microsomal membranes. This led us to simple α-D-mannoside derivative **1** and its α-1,6-linked disaccharide counterpart **2** (Fig. 1), which incorporate fluorescent coumarin residues and can be assembled by standard glycosylation techniques and copper-catalysed azide-alkyne cycloaddition (CuAAC) click chemistry [16]. These studies would therefore benchmark against our earlier work on related alkyl glycosides which, in assays with radiolabelled sugar nucleotide donors, we have demonstrated serve as acceptor substrates for GTs in Trypanosome [17] and mycobacterial membranes [18].

### 2.1 Chemical synthesis of fluorescent coumarin-based α-D-mannopyranoside derivatives

**Scheme 1:**
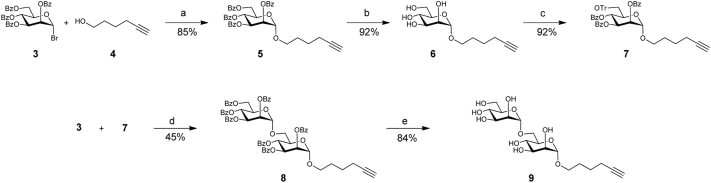
Synthesis of hexynyl glycosides **7** and **9**. Reagents and conditions: (a) AgOTf, CH_2_Cl_2_, –20 → 22 °C, 3.5 h; (b) 30 mM NaOMe in MeOH, RT, 4 h; (c) Ph_3_CCl, DMAP, Py, 37 °C, 40 h then BzCl, Py, RT, 1 h; (d) AgOTf, CH_2_Cl_2_, –20→RT °C, 18 h; e) 10 mM NaOMe in MeOH, RT, 24 h.

Hexynyl α-D-mannopyranoside (**6**) was synthesised in two steps starting from α-mannopyranosyl bromide **3** and 5-hexyn-1-ol (**4**) as outlined in Scheme 1. Glycosylation was performed by activation of glycosyl bromide **3** with AgOTf in the presence of 4 Å molecular sieves, which gave α-glycoside **5** in 85% yield. De-*O*-benzoylation of **5** afforded target α-D-mannopyranoside **6** in 92% yield. The corresponding α-Man-(1 → 6)-Man disaccharide **9** was synthesised in three steps from mannoside **6** by regioselective 6-*O*-tritylation followed by per-*O*-benzoylation to afford **7**, which was isolated in 92% yield. AgOTf-promoted glycosylation of trityl ether **7** with glycosyl bromide **3** gave α-linked disaccharide **8** in an unoptimised 45% yield. De-*O*-benzoylation of **8** afforded target disaccharide **9** in 84% yield. The α-configuration of the newly formed inter-sugar linkages in **5** and **8** were confirmed by the characteristic value of one bond C—H coupling of the anomeric carbon signal (^1^*J*_C-H_ ∼ 171 Hz) in ^1^H-coupled ^13^C NMR spectra of α-mannosides [19].

**Scheme 2:**
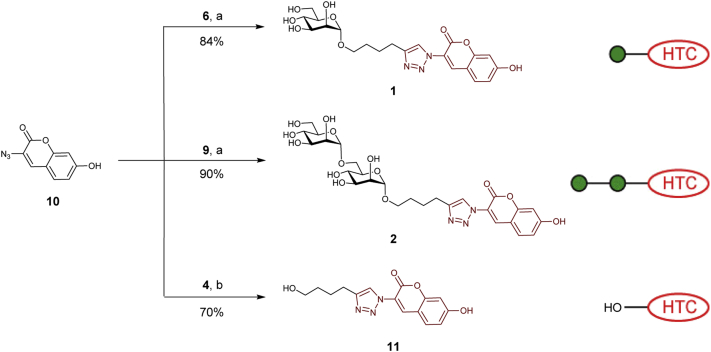
Synthesis of fluorescently labelled compounds **1**, **2** and **11** and their schematic representation. Reagents and conditions: (a) NaAsc, CuSO_4_, MeOH—H_2_O (1:1), RT, 2 h; (b) NaAsc, CuSO_4_, MeOH, RT, 2 h.

The alkyne functional groups of 5-hexyn-1-ol **4**, monosaccharide **6** and disaccharide **9** were used in fluorogenic CuAAC reactions with known non-fluorescent 3-azido-7-hydroxycoumarin (**10**) [20], generating the corresponding fluorescent adducts **1**, **2** and **11**, as outlined in Scheme 2. These reactions were generally complete after 2 h at room temperature, resulting in respectable yields (70–90%) of the required fluorescent adducts.^2^ The formation of 1,4-linked triazoles was confirmed by the presence of the characteristic triazole signal δ 8.17–8.25 in ^1^H NMR and δ 122.3–124.2 in ^13^C NMR spectra of compounds **1**, **2** and **11**.

### 2.2 Properties of fluorescent coumarin-based α-D-mannopyranoside derivatives

As is to be expected for a phenolic compound, the fluorescence emission of **2** was pH-dependent, with the intensity at pH 9 twice as large as the intensity in the range pH 3 to 6. The detection limit of **2** by TLC visualised by UV irradiation at 365 nm was *ca* 160 pmol, and *ca* 100 nM when detected in solution by fluorimeter. Euglena.

### 2.3 Benchmarking against published radiochemical assays

**Fig. 2:**
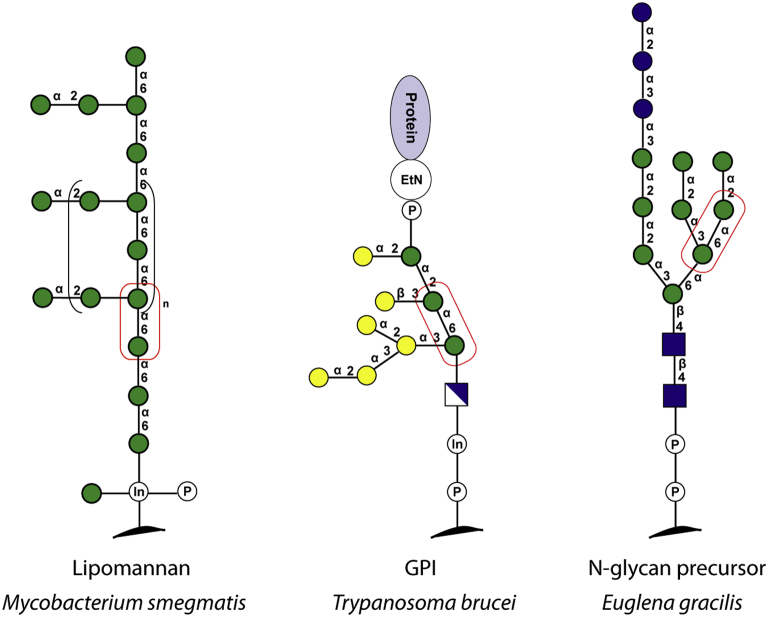
Schematic representation of structures of glycoconjugates from *Mycobacterium smegmatis*, *Trypanosoma brucei* and *Euglena gracilis*. The red boxes indicate disaccharide fragments mimicked by the synthetic acceptors **1** and **2**. (For interpretation of the references to colour in this figure legend, the reader is referred to the web version of this article.)

Published results from radiolabelled assays used to investigate the biosynthesis of cell surface glycoconjugates in *Mycobacterium smegmati* [18] and *Trypanosoma brucei* [17] were used as a benchmark for our practical fluorescence-based assay. In the first series of experiments we targeted α-1,6-ManT activities responsible for the biosynthesis of lipomannan in *M. smegmatis*; in the second series we investigated GalTs involved in the decoration of the core GPI anchor structures in *T. brucei*. Both lipomannan and GPI anchor structures have a common α-Man-1,6-α-Man disaccharide motif. Therefore, it was reasonable to envisage α-Man-HCT (**1**) and α-Man-1,6-α-Man-HCT (**2**) as mimics of intermediate structures involved in the biosynthesis of these glycoconjugates (Fig. 2). In essence, compounds **1** and **2** were anticipated to serve as generic mannoside acceptors for the detection of several glycosyltransferase activities, benchmarking our fluorescence-based assay as well as enabling further investigation of glycoconjugates biosynthesis in *Euglena gracilis*.

In preliminary studies (SI, Sections 2 and 3), we have demonstrated that fluorescence-based assays can be used to detect glycosyltransferase activities in *Mycobacterium smegmatis* and *Trypanosoma brucei*.

We showed that α-Man-1,6-α-Man-HCT (**2**) was active towards both α-1,6-ManT in *Mycobacterium smegmatis* membrane preparations, as well as α- and β-GalTs in *Trypanosoma brucei* microsomal preparations. Thus, incubation of **2** and GDP-Man with *Mycobacterium smegmatis* resulted in the formation of fluorescent α-1,6-linked manno-trioside and -tetraoside (Fig. S3 in SI). In similar experiments involving **2** and UDP-Gal in the presence of *Trypanosoma brucei* microsomal preparations, we observed addition of one galactose residue to fluorescent acceptor **2**, leading to the formation of α- and β-linked fluorescent trisaccharide products (Fig. S7 in SI). The presence of a fluorescent aglycone allowed quick access to information on the biotransformation reaction by TLC. The fluorescent label facilitated product purification by HPLC and product identification by enzymatic digestion followed by TLC. Most importantly, results of our fluorescence-based assays were consistent with data obtained in analogous studies that employed radiolabelled assays [17], [18]. Further details can be found in the Supplementary Information (section 2 and 3).

### 2.4 Mannosyltransferase activities in *Euglena gracilis*

Glycosyltransferases that participate in the biosynthesis of many, perhaps most, eukaryote glycoconjugates are located in the ER and Golgi apparatus [21], presenting a major challenge for their isolation in active form. In this study, our focus was simply on the detection of membrane-bound glycosyltransferases in *Euglena gracilis* microsomal membranes.

#### 2.4.1 Microsomal membranes as a source of glycosyltransferases

*Euglena* microsomal membranes were prepared following a literature procedure [22], from cells cultured in the dark in media supplemented with glucose (this approach provides far more biomass to work with than cells grown autotrophically in the light). After seven days, cells were harvested by centrifugation, lysed by ultrasonication and the microsomal membranes were obtained by ultracentrifugation over a sucrose gradient.

#### 2.4.2 Fluorescence assays to probe *Euglena gracilis* mannosyltransferase activities

**Fig. 3:**
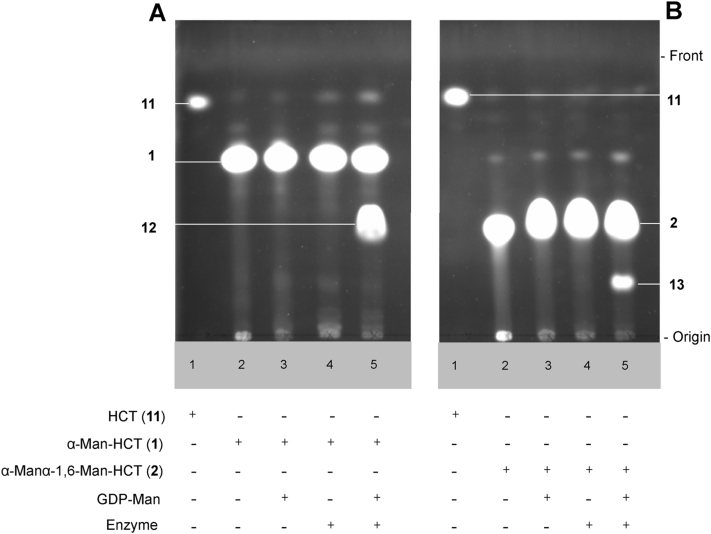
TLC analyses of fluorescence-based assays to assess mannosyltransferase activities in *Euglena gracilis* microsomal membranes. (A) α-Man-HCT (**1**) as acceptor; (B) α-Man-1,6-α-Man-HCT (**2**) as acceptor. Assay conditions: acceptor (2 mM) and donor GDP-Man (4 mM) were incubated for 24 h at 30 °C in HEPES/KOH (10 mM, pH 7.0) buffer supplemented with MgCl_2_ (10 mM), MnCl_2_ (10 mM), KCl (25 mM), glycerol (10%) in the presence of *E. gracilis* microsomal membranes (150 μL, 195 μg of total protein) in total reaction volume of 200 μL, TLC plates were eluted with CHCl_3_:MeOH:H_2_O (10:6:1) and visualised using mid-wave length range UV light. The components of each reaction mixture are shown below the corresponding TLC image and acceptors and products are shown to the side of each TLC image.

With microsomal membrane preps in hand, we established enzyme assays with fluorescent compounds **1** and **2** as acceptor substrates and GDP-Man as donor substrate. To ensure the absence of endogenous GDP-Man in the membrane preparation as well as to assess the potential enzymatic degradation of our fluorescent acceptors, two control assays were included containing either no enzyme or no donor substrate (Fig. 3A and B, lanes 3 and 4). The formation of fluorescent glycosylation products was evident from TLC analyses (Fig. 3A and B, lane 5).

TLC analysis of enzymatic reactions that involved α-Man-HCT (**1**) acceptor showed two distinct fluorescent bands (Fig. 3A, lane 5) corresponding to starting acceptor substrate **1** and product **12**. The latter possessed a similar R_*f*_ value to synthetic α-Man-1,6-α-Man-HCT (**2**) standard. This suggested a probable transfer of one mannose residue to acceptor substrate **1**. In a similar sense, TLC analysis of enzymatic reactions that involved α-Man-1,6-α-Man-HCT (**2**) acceptor also showed two distinct fluorescent bands (Fig. 3B, lane 5): one corresponding to starting acceptor substrate **2** and another more polar band, suggesting the formation of a trisaccharide product **13**. Unexpectedly, products fluorescence intensities visually indicated a higher enzymatic activity in the case of the simpler substrate, monosaccharide **1**, than of the disaccharide **2** In both sets of assays, only minimal *in situ* hydrolysis of the fluorescent acceptors was evident, indicating the absence of appreciable α-mannosidase activity.

#### 2.4.3 Purification of fluorescent products **12** and **13** by HPLC

**Fig. 4:**
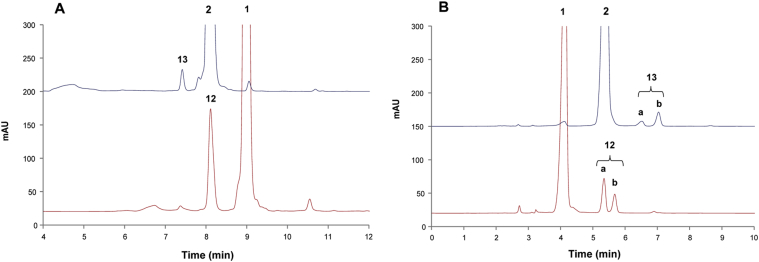
HPLC analyses of reaction mixtures obtained from incubation of fluorescent acceptors with GDP-Man in the presence of *Euglena gracilis* microsomal membranes. UV chromatograms of mannosylated products from α-Man-HCT (**2**) assay (red trace) and α-Man-1,6-α-Man-HCT (**1**) assay (blue trace). (A) Reverse phase purification of disaccharide **12** and trisaccharide **13** products. HPLC conditions: Phenomenex C18(2) (250 mm × 10 mm) chromatography column, mobile phase: water–CH_3_CN (0.1% TFA) (10–90% in 36 min), flow rate 5 mL/min), UV detector at 347 nm. (B) Normal phase HPLC purification of disaccharide **12** and trisaccharide **13** products. HPLC conditions: Phenomenex LUNA NH_2_ (250 mm × 10 mm) chromatography column, mobile phase: CH_3_CN-water (0.1% TFA) (10–80% in 32 min, flow rate 5 mL/min), UV detector at 347 nm. (For interpretation of the references to colour in this figure legend, the reader is referred to the web version of this article.)

In order to establish the structure of the above fluorescent products, they were first purified by reverse phase and then by normal phase HPLC methods. The former enabled isolation of disaccharide **12** and trisaccharide **13** as single HPLC peaks (Fig. 4A), while the latter resolved disaccharide products **12** and **13** into two peaks each, designated as **12a,b** and **13a,b** (Fig. 4B, red trace). The ratio of major (**12a**) to minor (**12b**) product was ca 2:1.

#### 2.4.4 Structural characterisation of fluorescent products

**Fig. 5:**
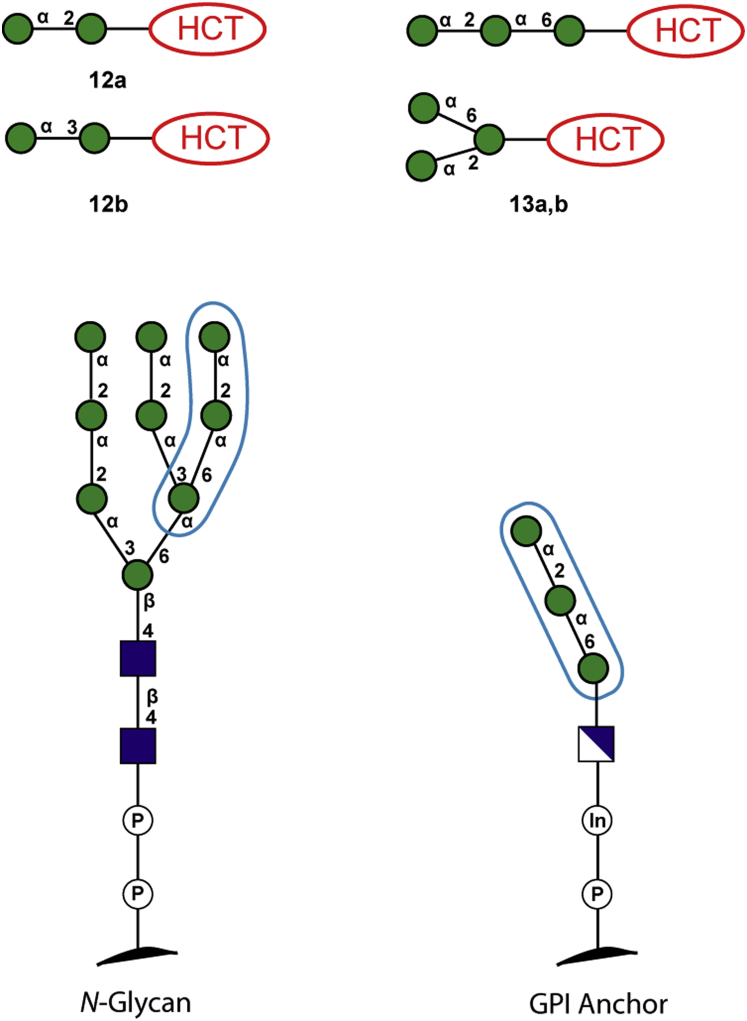
Symbolic representation of mannosyltransferase-catalysed reaction products **12** and **13** and their relationship with structures of *N*-glycan and GPI anchor precursors.

LC-MS analyses confirmed the formation of disaccharide and trisaccharide products from acceptors **1** and **2** (Fig. S10 in SI). The nature of the glycosidic linkages was then analysed by digestion with jack bean α-mannosidase [23], which showed that all products formed from **1** and **2** were α-linked mannosides (Figs. S11 and S12 in SI). Sequential digestion of **12a,b** and **13a,b** was then conducted with three different linkage-specific enzymes to assess the regioselectivity of the enzymatic mannosylation reactions. These experiments employed *Aspergillus saitoi* α-1,2-mannosidase [17b], *Xanthomonas manihotis* α-1,6-mannosidase and a *Xanthomonas manihotis* α-1,2/3-mannosidase [24] (for full details, see Supplementary Information sections 4 and 5). These experiments indicated that α-mannoside products derived from acceptor **1** were mainly α-1,2-linked, with only a minor proportion being α-1,3-linked. The same sequential enzymatic digestions of products derived from disaccharide acceptor **2** showed that solely α-1,2-mannosidic linkages have been formed. Based on these results, structures of disaccharide products **12a,b** and trisaccharide products **13a,b** can be proposed (Fig. 5).

The characterisation of products **12a,b** and **13a,b** confirmed that α-Man-HCT (**1**) and α-Man-1,6-α-Man-HCT (**2**) can act as acceptor substrates for α-1,2- and α-1,3-ManT present in *E. gracilis*. The oligosaccharide sequences of products **12a,b** and **13a,b** shown in Fig. 5 have a close resemblance to structures of fragments of *N*-glycans high mannose chain, thus indicating that the detected enzyme activities are most likely involved in the construction of *N*-glycans in *E. gracilis*. However, the possibility of the involvement of the observed α-1,2-ManT activity in GPI anchor biosynthesis cannot be ruled out.

### 2.5 Exploration of other glycosyltransferases in *Euglena gracilis*

The detection of α-1,2-ManT and α-1,3-ManT activities in *E. gracilis* microsomal membranes prompted us to look for other GT activities in these membrane preparations. Given that we achieved different outcomes with monosaccharide acceptor **1** and disaccharide acceptor **2**, we persisted with both compounds.

#### 2.5.1 Fluorescence assays to probe glycosyltransferase activities

**Fig. 6:**
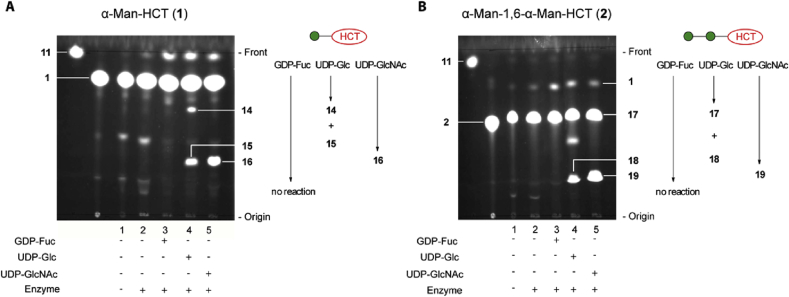
TLC analyses of enzymatic assays involving incubation of acceptors **1** and **2** with a number of sugar nucleotides in the presence of *Euglena gracilis* microsomal membranes. Fluorescent assay conditions: acceptor (2 mM) and donor (4 mM) were incubated for 24 h at 30 °C in HEPES/KOH (10 mM, pH 7.0) buffer supplemented with MgCl_2_ (10 mM), MnCl_2_ (10 mM), KCl (25 mM), glycerol (10%) in the presence of *E. gracilis* microsomal membrane (150 μL, 195 μg of total proteins) in total reaction volume of 200 μL. TLC plates were eluted with CHCl_3_:MeOH:H_2_O (10:8:2) and visualised using mid-wave length range UV light. Components of each reaction mixture are shown below the corresponding TLC trace and products are labelled on the side of the TLC image.

In order to more broadly assess GT activities, acceptor substrates **1** and **2** were incubated with UDP-d-Glc, UDP-d-GlcNAc, or GDP-l-Fuc, in the presence of *E. gracilis* microsomal membranes and the formation of fluorescent products was monitored by TLC (Fig. 6).

Assays with α-Man-HCT (**1**) in the presence of UDP-Glc revealed two new fluorescent bands, designated compounds **14** and **15** (Fig. 6A, lane 4). Judging by their R_*f*_ values, which were similar to that one of disaccharide **2**, faster moving compound **14** was tentatively assigned as a fluorescent disaccharide, while the much more polar product **15** was initially assigned as an oligosaccharide arising from the transfer of several glucose units. Assays with acceptor **1** and UDP-GlcNAc generated a single dominant polar product **16** (Fig. 6A, lane 5), which ran similarly on TLC to polar UDP-Glc-derived **15**.

Assays with disaccharide acceptor **2** led to a TLC outcomes that were qualitatively similar to those of monosaccharide acceptor **1**. Thus, in the presence of UDP-Glc two fluorescent products **17** and **18** were detected whereas in the presence of UDP-GlcNAc TLC showed that only one product, designated as compound **19**, was formed (Fig. 6B, lanes 4 and 5). In reactions with UDP-Glc, acceptor substrate turnover was demonstrably better in the case of disaccharide **2** compared to the monosaccharide **1**, in contrast to the mannosyltransferase activity described in Section 2.4 which provided lower turnover of disaccharide than monosaccharide acceptor. In contrast assays employing GDP-Fuc showed no turnover, in keeping with the anticipated lack of Fuc-Man linkages in eukaryotes.

#### 2.5.2 Characterisation of the less polar fluorescent products **14** and **17** from reactions of α-Man-HCT (**1**) and α-Man-1,6-α-Man-HCT (**2**) with UDP-Glc

**Fig. 7:**
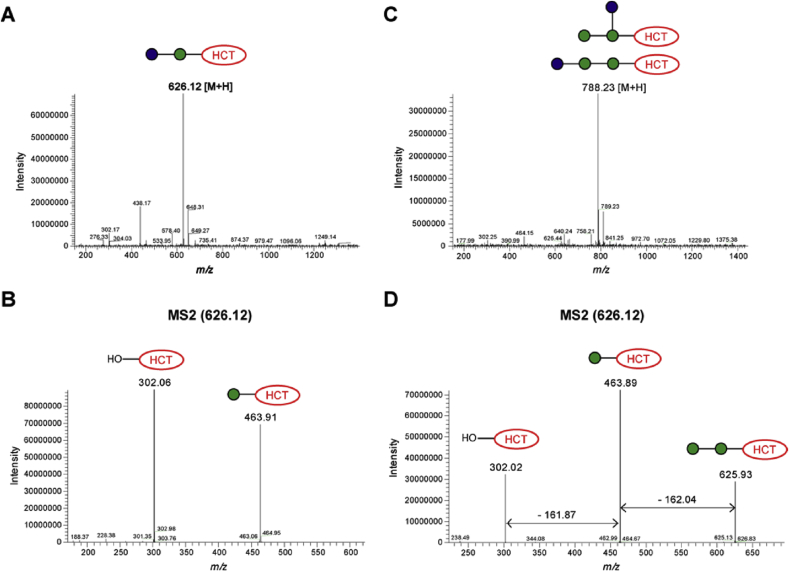
MS and MS2 analyses of products **14** (A, B) and **17** (C, D) obtained from enzymatic biotransformation with UDP-Glc in the presence of *Euglena gracilis* microsomal membranes.

In order to establish the nature of newly formed linkages in fluorescent products obtained from incubations of α-Man-HCT acceptor (**1**) and α-Man-1,6-α-Man-HCT acceptor (**2**) with UDP-Glc and UDP-GlcNAc, these products were purified by reverse phase HPLC and then subjected to LC-MS analysis. Analysis of LC-MS data for disaccharide **14** and trisaccharide **17** confirmed the addition of a single glucose residue to α-Man-HCT (**1**) and to α-Man-1,6-α-Man-HCT (**2**) (Fig. 7). The major peaks observed in the MS spectrum of **14** was *m/z* of 626.12 while for **17** an *m/z* of 788.23 was observed, corresponding to [M+H]^+^ signals of disaccharide and trisaccharide, respectively (Fig. 7B). The tandem mass-spectrometry (MS2) fragmentation data for both compounds showed a sequential loss of hexose units (*m/z* 162) and ultimate release of fluorescent HCT aglycone (**11**) (*m/z* 302.00) (Fig. 7C). Since the glucosylated adducts were the minor products obtained for both acceptors, specific details of the newly formed linkages were not pursued further.

#### 2.5.3 Characterisation of the more polar fluorescent products **15** and **18** from reactions of α-Man-HCT (**1**) and α-Man-1,6-α-Man-HCT (**2**) with UDP-Glc

**Fig. 8:**
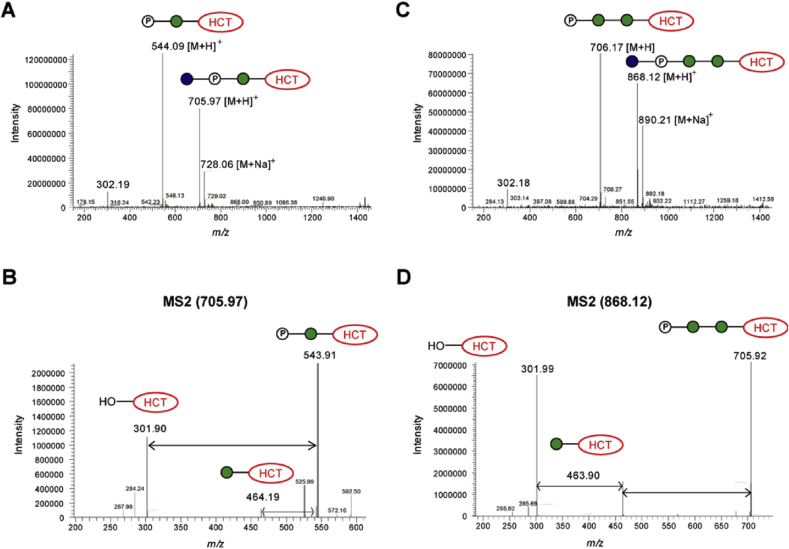
MS and MS2 analyses of products **15** (A, B) and **18** (C, D) obtained from enzymatic biotransformation with UDP-Glc in the presence of *Euglena gracilis* microsomal membranes.

As described in section 2.5.1, TLC mobilities of glucosylated **15** and **18** gave R_*f*_ values that suggested the presence of more than one added sugar residue on acceptors **1** and **2**. In contrast, LC-MS data analyses revealed additions of no more than one sugar residue in each case, with a slightly higher (+80) *m/z* than that calculated for the straightforward addition of a single glucose residue (Fig. 8). On closer inspection of the MS and MS2 data, it became evident the masses observed for products **15** and **18** were consistent with the transfer of a glucose phosphate residue to the acceptor, which immediately accounted for the unexpected polarity of these compounds. While it is formally possible that a glucose residue and a phosphate residue may have been separately transferred to the acceptor, the *en bloc* transfer of a sugar phosphate seemed more plausible on biosynthetic grounds. However, to the best of our knowledge, no enzyme is known that transfers Glc-1-P *en-bloc* onto an acceptor glycan; on the other hand, the corresponding transfer of GlcNAc-1-P is central to the generation of mannose-6-phosphate in lysosomal targeting [25], which informed our thinking about the likely structure of the polar products derived from α-Man-HCT (**1**) and α-Man-1,6-α-Man-HCT (**2**) with UDP-GlcNAc.

#### 2.5.4 Identification of fluorescent polar products **16** and **19** obtained from incubation of α-Man-HCT (**1**) and α-Man-1,6-α-Man-HCT (**2**) with UDP-GlcNAc

**Fig. 9:**
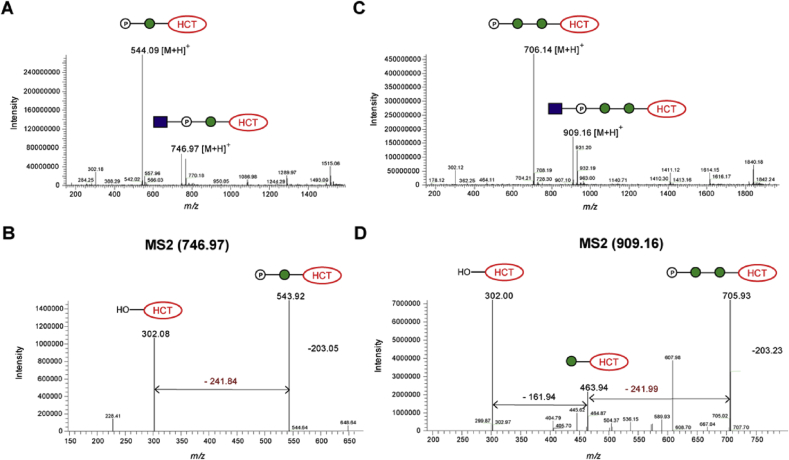
LC-MS analyses of fluorescent products **16** (A, B) and **19** (C, D) obtained from enzymatic biotransformation with UDP-GlcNAc in the presence of *Euglena gracilis* microsomal membranes.

Purified fluorescent compounds **16** and **19**, obtained from incubation of compounds **1** and **2** with UDP-GlcNAc in the presence of *E. gracilis* microsomal membranes, were subjected to LC-MS analysis. These analyses showed similar overall outcomes to those described above for fluorescent **15** and **18**, derived from UDP-Glc (Fig. 9). Briefly, MS revealed *m/z* signals of 746.97 for **16** and *m/z* of 909.16 for **19**, which corresponded to [M+H]^+^ molecular ions of phosphodiester-linked disaccharide and trisaccharide, respectively (Figs. 9B1 and 10B2). The MS2 fragmentation data for both compounds showed loss of GlcNAc, leaving phosphorylated versions of α-Man-HCT (**1**) and α-Man-1,6-α-Man-HCT (**2**).

**Fig. 10:**
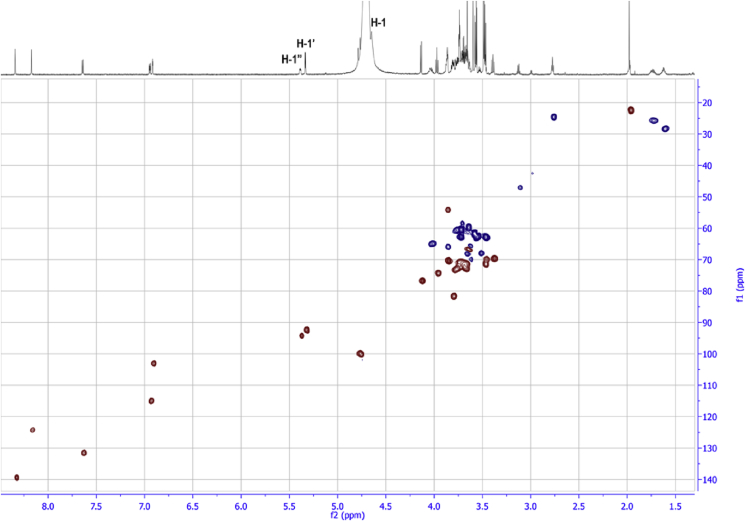
HSQC NMR (25 °C, D_2_O) of compound **19**.

Ahead of scale up (*vide infra*), and in order to gain further evidence for phosphodiester formation, a series of sequential digestions with alkaline phosphatase and TFA were conducted (see Figs. S17–S19 in SI). These experiments showed the lack of sensitivity of **15**/**18** (generated with UDP-Glc) and **16**/**19** (generated with UDP-GlcNAc) to alkaline phosphatase, unless the compounds were pre-treated with TFA. This is in keeping with the proposed inter-sugar phosphodiester linkages.

#### 2.5.5 Confirmation of fluorescent product **19** structure by NMR spectroscopy

In order to substantiate the phosphodiester structures proposed above, an enzymatic reaction involving UDP-GlcNAc and acceptor **2** in the presence of *E. gracilis* microsomal membranes was scaled up. Under standard conditions (24 h at 30 °C, section 4.7) enzymatic conversion of acceptor **2** into product **19** was estimated as 10%. On addition of an extra portion of *E. gracilis* microsomal membranes after an initial 24 h at 30 °C and further incubation for 24 h, the isolated yield of **19** was increased to 43%, amounting to 0.48 mg of material, which was subjected to detailed NMR analysis.

**Fig. 11:**
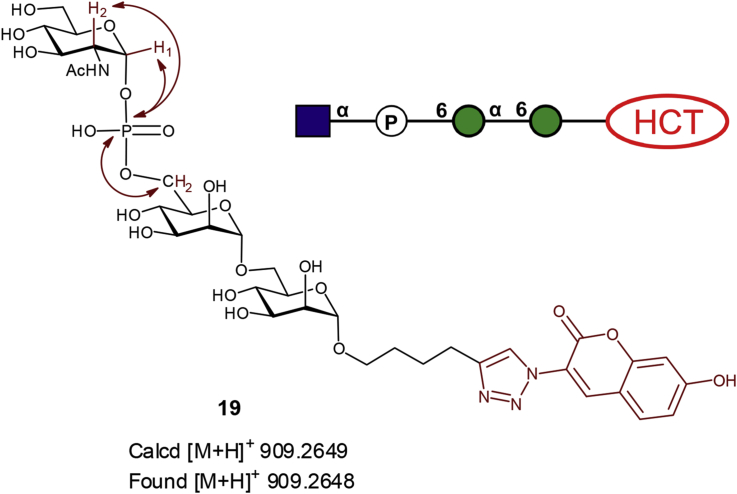
Structure of fluorescent phosphodiester-linked compound **19** as determined by NMR and HRMS. Double arrows indicate ^1^H—^31^P couplings observed in the ^1^H—^31^P HMBC spectrum of **19**.

The ^1^H NMR spectrum of phosphodiester-linked **19** displayed all four coumarin signals in the aromatic region and a singlet at δ 8.26 ppm for the 1,4-triazole ring proton. The signal at δ 5.48 ppm was assigned to the anomeric proton of the GlcNAc residue based on 2D NMR analyses, which showed a ^1^H—^13^C cross-peak in HSQC spectrum at δ_C_ 94.3 ppm (Fig. 10). The other two anomeric signals of the mannose residues, hidden in ^1^H NMR spectra under the solvent peak, were identified through the ^1^H—^13^C HSQC correlation with H-1/C-1 at 5.42/93 ppm and 4.62/100 ppm (Fig. 10). The signal of the anomeric proton of the GlcNAc residue in **19** appears as a characteristic doublet of doublets as a result of both ^3^*J* coupling between H-1 and H-2 (*J*_1,2_ = 3.6 Hz) and ^1^*J* coupling between H-1 and ^31^P (*J*_1,P_ = 7.2 Hz). The small values for both coupling constants are as expected for GlcNAc residue with α-configuration [26]. Only one signal can be found in ^31^P NMR spectrum of compound **19** with a chemical shift (−1.26 ppm) characteristic for phosphodiester linkages [27], 2D analysis of ^1^H—^31^P HMBC spectrum of **19** revealed cross-peaks at δ 5.48 ppm, which corresponds to H-1 of GlcNAc, at 3.95 ppm, which corresponds to H-2 of GlcNAc, and at 4.14 ppm, which corresponds to H-6 of Man' residue, therefore defining that the precise position of the phosphodiester linkage is as shown in Fig. 11. High resolution MS (HRMS) confirmed the molecular formula of **19** as C_35_H_50_N_4_O_22_P (SI, Section 8). These NMR and MS data confirmed that the sugar-phosphate transferase enzyme present in *E. gracilis* microsomal membranes catalyses transfer of a GlcNAc-P sugar residue from the UDP-GlcNAc to the 6′-hydroxyl of the mannose sugar residue of acceptor substrate **2** to form **19**, which possesses a phosphodiester-linkage (Fig. 11).

With both fluorescent monosaccharide **1** and disaccharide **2** acceptor substrates it was possible to detect *N*-acetylglucosamine-1-phosphate transferase activity. Transcriptom analysis [7] for the predicted *E. gracilis* GlcNAc-P-Tase using the amino acid sequence of the *Homo sapiens* gene yielded a hit that is predicted to be a transmembrane protein consisting of 1140 amino acids (124 kDa). This putative enzyme shares 26% identity with the catalytic domain of the *Homo sapiens* enzyme and 26% identity with the *Dictyostelium discoideum* enzyme.

## 3 Conclusions

In this study we set out to develop convenient assays for the analysis of carbohydrate-active enzymes that rely upon fluorescent acceptor substrates, which may be used in place of established radiochemical approaches that generally rely on isotope-labelled donor substrates. Our goal was initially to benchmark the fluorescent approach against our earlier radiochemical work with glycosyltransferase activities found in mycobacterial and trypanosomal membranes.

The installation of a fluorophore on acetylenic alkyl mannosides was easily achieved through click chemistry with a readily accessible azidocoumarin precursor, giving adducts that were straightforward to analyse by TLC, by reverse phase and by normal phase HPLC, providing a convenient interface to inline mass spectrometry analyses. The resulting fluorescent α-mannoside (**1**) and α-1,6-linked dimannoside (**2**) proved to be acceptor substrates for α-1,6-ManT activities in mycobacterial membranes and α- and β-GalT activities in trypanosomal membranes, in keeping with data reported previously from our radiochemical work [17], [18].

Similar experiments with *Euglena* membranes detected the presence of α-1,3- and/or α-1,2-ManT activities when α-mannoside (**1**) and α-1,6-linked dimannoside (**2**) were used as acceptor substrates. Interestingly, monosaccharide (**1**) proved to be a more efficient acceptor than disaccharide (**2**), although the latter was less promiscuous, accepting α-1,3-linkages only. Wider-ranging assays with the same fluorescent acceptors demonstrated no turnover in the presence of GDP-L-Fuc, while UDP-GlcNAc gave rise to a single very polar product and UDP-Glc generated both an apparent glycosylation product as well as a much more polar compound. Detailed LC-MS analyses confirmed the conventional glucosylation of acceptors **1** and **2** as a minor reaction, in addition to the production of much more polar material that from MS analysis proved to be an unexpected glucosyl phosphate diester of the acceptors, namely compounds **15** and **18**. Where UDP-GlcNAc was employed as the donor, only phosphodiester products were formed. In light of the efficiency of this latter reaction, the product from disaccharide **2**, compound **19**, was isolated and further characterised by NMR spectroscopy, confirming it to be the α-GlcNAc-1,6-phospho-α-Man-1,6-α-Man product. Bioinformatic analysis confirmed the presence of a putative GlcNAc phosphotransferase in *Euglena*, albeit one with low sequence identity to the established human and slime mould enzymes.

In summary, these studies illustrate the utility of fluorescent acceptors based on triazolyl-coumarin for the TLC, HPLC and mass spectrometry analysis of glycosyltransferase activities in membrane preparations from a number of species. They also confirmed the presence of ManTs and GlcNAc phosphotransferase in *Euglena* membranes, along with the unexpected presence of a similar activity that transfers glucose-1-phosphate *en bloc* to α-mannoside acceptors. These readily accessible fluorescent acceptors open the way for wider analysis of the under-explored repertoire of carbohydrate-active enzymes in bacterial and protozoal systems.

## 4 Experimental

### 4.1 General methods

Chemicals were purchased as reagent grade and used without further purification. All moisture-sensitive reactions were performed under a dry nitrogen atmosphere using oven-dried glassware. Anhydrous solvents were purchased from Sigma Aldrich and CH_2_Cl_2_ was freshly distilled from calcium hydride prior to use. Reactions were monitored by thin-layer chromatography (TLC) aluminium plates (Silica Gel 60 F_254_, E. Merck) with indicated eluents. Compounds were visualised under UV light (λ 254 nm) and by dipping in ethanol-sulphuric acid (95:5, v/v) followed by heating. Fluorescent products were purified on semi-preparative TLC on pre-coated silica gel aluminium plates (Silica Gel 1000 UV254, Analtech). Flash chromatography was performed on a Biotage Isolera MPLC system using pre-packed silica gel cartridges. Nuclear magnetic resonance spectra were recorded on a Bruker Avance III 400 NMR or Bruker Avance 800 spectrometer at 298 K. Chemical shifts (δ) are reported in parts per million (ppm) with respect to internal tetramethylsilane (TMS) in CDCl_3_ and residual HOD signal in D_2_O. NMR signal assignments were made with the aid of COSY and HSQC experiments. The mode of atom numbering of HCT aglycone used for NMR assignments is as shown for compound **11** below.

**Figure:**
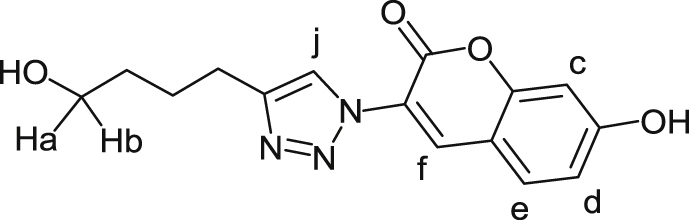

Optical rotations were measured at 20 °C in 1 mL cell in the stated solvent using a Perkin-Elmer 341 polarimeter equipped with a sodium lamp. For HRMS, the samples were diluted into 50% methanol/0.1% formic acid and infused into a Synapt G2-Si mass spectrometer (Waters, Manchester, UK) at 5–10 μl min^−1^ using a Harvard Apparatus syringe pump. The mass spectrometer was controlled by Masslynx 4.1 software (Waters). It was operated in resolution and positive ion mode and calibrated using sodium formate. The sample was analysed for 2 min with 1 s MS scan time over the range of 50–1200 *m/z* with 3.5 kV capillary voltage, 40 V cone voltage, 120 °C cone temperature. Leu-enkephalin peptide (1 ng μl^−1^, Waters) was infused at 10 μl min^−1^ as a lock mass (m/z 556.2766) and measured every 10 s. Spectra were generated in Masslynx 4.1 by combining a number of scans, and peaks were centred using automatic peak detection with lock mass correction. For elemental composition prediction the spectrum elemental composition tool in the Masslynx 4.1 software was used.

*Euglena gracilis* var *saccharophila* Klebs (strain 1224/7) was supplied by culture collection of alga and protozoa (CCAP). The original EG:JM medium was prepared according to recipes from CCAP (www.ccap.ac.uk). HPLC was carried out using a Dionex HPLC system. Total protein concentration was determined using commercially available Bradford*Ultra*, that was purchased from Expedion [28], calibrated against a BSA standard curve. cOmplete™, Mini EDTA-free Protease Inhibitor cocktail was purchased from Sigma Aldrich. Jack bean α-mannosidase were obtained from Sigma Aldrich, *Xanthomonas manihotis* α-1,6-mannosidase from New England Biolabs and *Aspergillus saitoi* α-1,2-mannosidase from Prozyme. Purification of fluorescent products was carried out using a Dionex HPLC system with semi-prep normal phase HPLC Phenomenex Luna NH_2_ (250 × 10 mm) and or C18 reverse phase column (Phenomenex Luna C18(2) 100 Å, 250 × 10 mm) and visualised using UV detector at 347 nm. LC-MS was carried out using a Thermo Finnigan Surveyor HPLC system, with either reverse phase (C18) or normal phase (NH_2_) columns, equipped with a UV detector or an LCQ Deca XP plus (ion trap) MS detector (Thermo Fisher Scientific Inc., Hemel Hempstead, UK). The following HPLC conditions were used for characterisation of compounds **14**–**19**: Kinetex C18 column (50 mm × 2.1 mm, 2.6 μm), 0.1% aq. TFA-CH_3_CN (10–90% over 18 min), flow rate 0.3 mL/min), selected ion monitoring mode with UV detection at 347 nm. TLC separations were performed at room temperature on aluminium-backed silica gel 60 F_254_ or glass plated silica gel 60 Å TLC plates. Samples from enzymatic transformations were applied onto a TLC plate in 2 μL aliquots and dried with a hairdryer between applications. The TLC plates were eluted with either CHCl_3_/MeOH/H_2_O (10:6:1) or (10:8:2) solvent mixture, air dried and directly visualised with the gel imager (Synoptics 2.0 MP) and processed with the GENE SYS ver 1.2.5.0 program.

### 4.2 Chemical synthesis of non-fluorescent and fluorescent α-d-mannopyranoside derivatives

#### 4.2.1 Hexyn-5-yl 2,3,4,6-tetra-O-benzoyl-α-d-mannopyranoside (**5**)

To a solution of 2,3,4,6-tetra-*O*-benzoyl-α-d-mannopyranosyl bromide [29] (**3**) (11.7 g, 17.9 mmol) and 5-hexyn-1-ol (**4**) (2.1 g, 21.5 mmol) in anhydrous CH_2_Cl_2_ (40 mL) containing 4 Å mol. sieves cooled at −20 °C, a solution of AgOTf (6.4 g, 25.1 mmol) in anhydrous toluene (160 mL) was added. The reaction mixture was allowed to warm to room temperature and stirred for 3.5 h. The reaction mixture was neutralised with Et_3_N (2.0 mL), filtered through Celite and concentrated under reduced pressure. The residue was purified by flash column chromatography (hexane/EtOAc 7:3) to give protected hexynyl mannoside **5** as a colourless amorphous solid (10.2 g, 85%). R_*f*_ 0.6 (hexane/EtOAc, 6:4); [α]_D_ - 55.0 (*c* 1.0, CHCl_3_); ^1^H NMR (400 MHz, CDCl_3_): δ 8.11–8.05 (4H, m, Ar), 7.98–7.96 (2H, m, Ar), 7.85–7.83 (2H, m, Ar), 7.61–7.24 (12H, m, Ar), 6.12 (1H, t, *J*_3,4_ ≈ *J*_4,5_ = 8 Hz, H-4), 5.93 (1H, dd, *J*_2,3_ = 3.4 Hz, *J*_3,4_ = 8.0 Hz, H-3), 5.71 (1H, dd, *J*_1,2_ = 1.6 Hz, *J*_2,3_ = 3.4 Hz, H-2), 5.10 (1H, d, *J*_1,2_ = 1.6 Hz, H-1), 4.70 (1H, dd, *J*_5,6a_ = 2.5 Hz, *J*_6a,6b_ = 12.1 Hz, H-6a), 4.50 (1H, dd, *J*_5,6b_ = 4.5 Hz, *J*_6a,6b_ = 12.1 Hz, H-6b), 4.45–4.41 (1H, m, H-5); 3.90–3.84 (1H, m, OC*H*_*a*_H_b_), 3.66–3.58 (1H, m, OCH_a_*H*_*b*_), 2.30–2.26 (2H, m, OCH_2_CH_2_CH_2_C*H*_*2*_CCH), 2.00 (1H, t, *J* = 2.6 Hz OCH_2_CH_2_CH_2_CH_2_CC*H*), 1.86–1.81 (2H, m, OCH_2_C*H*_2_CH_*2*_CH_2_CCH), 1.72–1.66 (2H, m, OCH_2_CH_*2*_C*H*_2_CH_2_CCH); ^13^C NMR (100.6 MHz, CDCl_3_): δ 166.1–165.4 (C=O), 133.4–128.3 (aromatic C), 98.4 (*J*_C-H_ = 171.9 Hz, C-1), 84.0, 70.6 (C-2), 70.1 (C-3), 68.9 (C-4), 68.8, 68.1, 67.0 (C-5), 62.9 (C-6), 28.4, 25.0, 18.2; HRMS (ESI) *m/z* calcd for C_40_H_36_O_10_Na^+^ (M+Na]^+^): 699.2201, found 699.2202.

#### 4.2.2 Hexyn-5-yl α-d-mannopyranoside (**6**)

A solution of protected hexynyl mannoside **5** (3.10 g, 4.8 mmol) in absolute MeOH (60 mL) was treated with 1 M NaOMe in MeOH (1.9 mL, 1.9 mmol). The solution was kept at room temperature for 4 h, neutralised with ion-exchange resin (Amberlite IR-120 H^+^) and concentrated under reduced pressure. The resulting residue was re-dissolved in water (50 mL) and the aqueous phase was washed with dichloromethane (3 × 50 mL). The aqueous extract was evaporated to give unprotected hexynyl mannoside **6** as a colourless syrup (1.1 g, 92%). *R*_*f*_ 0.2 (CH_2_Cl_2_/MeOH, 9:1); [α]_D_ +72.0 (*c* 1.0, CHCl_3_); ^1^H NMR (400 MHz, D_2_O): δ 4.87 (1H, d, *J*_1,2_ = 1.7 Hz, H-1), 3.94 (1H, dd, *J*_1,2_ = 1.7 Hz, *J*_2,3_ = 3.4 Hz, H-2), 3.89 (1H, dd, *J*_5,6a_ = 1.8 Hz, *J*_6a,6b_ = 12.4 Hz, H-6a), 3.81–3.73 (3H, m, H-3, H-6b, OC*H*_*a*_H_b_), 3.68–3.55 (3H, m, H-4, H-5, OCH_a_*H*_*b*_), 2.28–2.23 (3H, m, OCH_2_CH_2_CH_2_C*H*_*2*_CCH, OCH_2_CH_2_CH_2_CH_2_CC*H*), 1.77–1.68 (2H, m, OCH_2_C*H*_*2*_CH_2_CH_2_CCH), 1.66–1.56 (2H, m, OCH_2_CH_2_C*H*_*2*_CH_2_CCH); ^13^C NMR (100.6 MHz, D_2_O): δ 98.4 (C-1), 84.2, 71.5 (C-5), 69.4 (C-3), 68.8 (C-2), 67.9, 66.1, 65.5 (C-4), 59.7 (C-6), 26.5, 23.3, 16.1; HRMS (ESI) *m/z* calcd for C_12_H_20_O_6_Na^+^ (M+Na]^+^): 283.1152, found 283.1147.

#### 4.2.3 Hexyn-5-yl 2,3,4-tri-O-benzoyl-6-O-trityl-α-d-mannopyranoside (**7**)

To a solution of unprotected hexynyl mannoside **6** (1.2 g, 4.4 mmol) in anhydrous pyridine (15 mL) triphenylmethyl chloride (1.8 g, 6.6 mmol) and 4-dimethylaminopyridine (108 mg, 0.88 mmol) were added. The reaction mixture was heated at 37 °C for 40 h, diluted with pyridine (45 mL) and cooled down to 0 °C prior to benzoyl chloride (51.0 mL, 44.2 mmol) addition. The mixture was stirred for 1 h at room temperature and ice-cold water was carefully added. The product was extracted with CH_2_Cl_2_ (3 × 75 mL), the organic extracts were combined and washed with ice-cold 1 M HCl (3 × 75 mL), saturated aqueous NaHCO_3_ solution (4 × 75 mL), dried (MgSO_4_), filtered and concentrated under reduced pressure. The obtained residue was purified by flash column chromatography (hexane/EtOAc 7:3) to give ester protected trityl ether **7** as a colourless amorphous solid (3.3 g, 92%). R_*f*_ 0.77 (hexane/EtOAc, 6:4); [α]_D_ −95.0 (*c* 1.0, CHCl_3_); ^1^H NMR (400 MHz, CDCl_3_): δ 8.16–8.10 (2H, m, Ar), 7.85–7.82 (2H, m, Ar), 7.75–7.73 (2H, m, Ar), 7.50–7.40 (10H, m, Ar), 7.33–7.24 (6H, m, Ar), 7.17–7.07 (8H, m, Ar), 6.02 (1H, t, *J* = 10.2, H-4), 5.78 (1H, dd, *J*_2,3_ = 3.4 Hz, *J*_3,4_ = 10.2 Hz, H-3), 5.67 (1H, dd, *J*_1,2_ = 1.6 Hz, *J*_2,3_ = 3.4 Hz, H-2), 5.12 (1H, d, *J*_1,2_ = 1.6 Hz, H-1), 4.20–4.16 (1H, m, H-5), 3.93–3.87 (1H, m, OC*H*_*a*_H_b_), 3.65–3.59 (1H, m, OCH_a_*H*_*b*_), 3.38 (1H, dd, *J*_5,6a_ = 2.2 Hz, *J*_6a,6b_ = 10.5 Hz, H-6a), 3.28 (1H, dd, *J*_5,6b_ = 4.8 Hz, *J*_6a,6b_ = 10.5 Hz, H-6b), 2.30–2.26 (2H, m, OCH_2_CH_2_CH_2_C*H*_*2*_CCH), 1.97 (1H, t, *J* = 2.6 Hz, OCH_2_CH_2_CH_2_CH_2_CC*H*), 1.86–1.81 (2H, m, OCH_2_C*H*_*2*_CH_2_CH_2_CCH), 1.74–1.66 (2H, m, OCH_2_CH_2_C*H*_*2*_CH_2_CCH); ^13^C NMR (100.6 MHz, CDCl_3_): δ 165.7–165.1 (C=O), 146.8–126.8 (aromatic C), 97.5 (C-1), 84.0, 70.9 (C-2), 70.6 (C-3), 70.9 (C-5), 68.7, 67.7, 67.0 (C-4), 62.3 (C-6), 28.4, 25.1, 18.2; HRMS (ESI) *m/z* calcd for C_52_H_46_O_9_Na^+^ (M+Na]^+^): 837.3034, found 837.3029.

#### 4.2.4 6-O-Hexyn-5-yl (2,3,4,6-tetra-O-benzoyl-α-d-mannopyranosyl)-2,3,4-tri-O-benzoyl-α-d-mannopyranoside (**8**)

To a solution of esterified trityl ether **7** (3.7 g, 4.5 mmol) and mannosyl bromide **3** (4.5 g, 6.8 mmol) in anhydrous CH_2_Cl_2_ containing 4 Å mol. sieves, at −20 °C, a solution of AgOTf (1.6 g, 6.4 mmol) in anhydrous toluene (110 mL) was added. The reaction mixture was allowed to warm to room temperature and stirred for 18 h, neutralised with Et_3_N (3.0 mL), filtered through Celite and concentrated under reduced pressure. The resulting residue was purified by flash column chromatography (hexane/EtOAc 7:3) to give protected hexynyl disaccharide **8** as a colourless amorphous solid (2.3 g, 45%). R_*f*_ 0.6 (hexane/EtOAc, 1:1); [α]_D_ - 54.0 (*c* 1.0, CHCl_3_); ^1^H NMR (400 MHz, CDCl_3_): δ 8.17–8.15 (2H, m, Ar), 8.04–7.98 (8H, m, Ar), 7.87–7.84 (4H, m, Ar), 7.58–7.48 (6H, m, Ar), 7.44–7.34 (10H, m, Ar), 7.30–7.21 (5H, m, Ar), 6.11–6.06 (2H, m, H-4, H-4′), 6.00 (1H, dd, *J*_2′,3′_ = 3.2 Hz, *J*_3′,4′_ = 10.1 Hz, H-3′), 5.94 (1H, dd, *J*_2,3_ = 3.3 Hz, *J*_3,4_ = 10.1 Hz, H-3), 5.78 (1H, dd, *J*_1′,2′_ = 1.5 Hz, *J*_2′,3′_ = 3.2 Hz, H-2′), 5.75 (1H, dd, *J*_1,2_ = 1.6 Hz, *J*_2,3_ = 3.3 Hz, H-2), 5.15 (1H, d, *J*_1′,2′_ = 1.5 Hz, H-1′), 5.12 (1H, d, *J*_1,2_ = 1.6 Hz, H-1), 4.51 (1H, dd, *J*_5′,6a′_ = 2.4 Hz, *J*_6a′,6b′_ = 12.2 Hz, H-6′a), 4.44–4.37 (2H, m, H-5′ H-5), 4.30 (1H, dd, *J*_5′,6a′_ = 4.2 Hz, *J*_6a′,6b′_ = 12.2 Hz, H-6′b), 4.13 (1H, dd, *J*_5,6a_ = 5.4 Hz, *J*_6a,6b_ = 10.9 Hz, H-6b), 4.00–3.95 (1H, m, OC*H*_*a*_H_b_), 3.78 (1H, dd, *J*_5a,6b_ = 2.0 Hz, *J*_6a,6b_ = 10.8 Hz, H-6a), 3.70–3.64 (1H, m, OCH_a_*H*_*b*_), 2.35–2.31 (2H, m, OCH_2_CH_2_CH_2_C*H*_*2*_CCH), 1.98 (1H, t, *J* = 2.6 Hz, OCH_2_CH_2_CH_2_CH_2_CC*H*), 1.95–1.87 (2H, m, OCH_2_C*H*_*2*_CH_2_CH_2_CCH), 1.80–1.72 (2H, m, OCH_2_CH_2_C*H*_*2*_CH_2_CCH); ^13^C NMR (100.6 MHz, CDCl_3_): δ 166.0–165.1 (C=O), 133.5–132.9 (aromatic C), 130.1–128.3 (aromatic C), 97.8 (C-1), 97.7 (C-1′), 84.1, 70.6 (C-2), 70.3 (C-2′), 70.2 (C-3; 3′), 69.6 (C-5), 68.9 (C-5′), 68.7, 68.1, 67.0 (C-6), 66.7 (C-4), 62.3 (C-6′), 28.5, 25.1, 18.2; HRMS (ESI) *m/z* calcd for C_67_H_58_O_18_Na^+^ (M+Na]^+^): 1173.3515, found 1173.3517.

#### 4.2.5 Hexyn-5-yl α-d-mannopyranosyl-(1 → 6)-α-d-mannopyranoside (**9**)

A solution of protected hexynyl disaccharide **8** (880 mg, 0.8 mmol) in absolute MeOH (60 mL) was treated with 1 M NaOMe in MeOH (0.5 mL, 0.54 mmol). The solution was kept at room temperature for 24 h, neutralised with ion-exchange resin (Amberlite IR-120 H^+^) and concentrated under reduced pressure. The resulting residue was purified by flash column chromatography (CH_3_CN/H_2_O/NH_3_ 6:3:1) to give unprotected hexynyl disaccharide **9** as a colourless amorphous solid (323 mg, 84%). R_*f*_ 0.66 (CH_3_CN/H_2_O/NH_3_ 6:3:1); [α]_D_ + 80.0 (*c* 1.0, MeOH); 1H NMR (400 MHz, D_2_O): δ 4.91 (1H, d, *J*_1′,2′_ = 1.7 Hz, H-1′), 4.87 (1H, d, *J*_1,2_ = 1.2 Hz, H-1), 4.00 (1H, dd, *J*_1′,2′_ = 1.7 Hz, *J*_2′,3′_ = 3.4 Hz, H-2′), 4.00–3.95 (2H, m, H-2; H-6b′), 3.92 (1H, dd, *J*_5′,6a′_ = 1.6 Hz, *J*_6a′,6b′_ = 11.8 Hz, H-6a′), 3.85 (1H, dd, *J*_2,3_ = 3.4 Hz, J_3,4_ = 9.0 Hz, H-3), 3.81–3.65 (8H, m, H-4′, H-4, H-6a, H-6b, OC*H*_*a*_H_b_, H-3′, H-5′, H-5), 3.62–3.57 (1H, m, OCH_a_*H*_*b*_), 2.39 (1H, t, *J* = 2.6 Hz, OCH_2_CH_2_CH_2_CH_*2*_CC*H*), 2.29–2.25 (2H, m, OCH_2_CH_2_CH_2_C*H*_*2*_CCH), 1.78–1.71 (2H, m, OCH_2_C*H*_*2*_CH_2_CH_2_CCH), 1.67–1.58 (2H, m, OCH_2_CH_2_C*H*_*2*_CH_2_CCH); ^13^C NMR (100.6 MHz, D_2_O): δ 99.8, 99.4, 85.8, 72.6 (C-5), 70.9 (C-3, 3′), 70.0 (C-2), 69.9 (C-2′), 67.3, 67.3, 66.7 (C-4), 66.6 (C-4′), 65.7 (C-6), 60.9 (C-6′), 27.6, 24.5, 17.2; HRMS (ESI) *m/z* calcd for C_18_H_30_O_11_Na^+^ (M+Na]^+^): 445.1680, found 445.1677.

#### 4.2.6 4-(1-(7-Hydroxy-coumarin-3-yl)-1H-1,2,3-triazol-4-yl)-)butyl α-d-mannopyranoside (**1**)

A solution of hexynyl mannoside **6** (17 mg, 0.06 mmol) and 3-azido-7-hydroxy coumarin (**10**) (16 mg, 0.05 mmol) in MeOH/H_2_O (1:1) (1 mL) was treated with 1 M aq. CuSO_4_ (10 μL) and 1 M aq. NaAsc (25 μL). The reaction mixture was allowed to stir at room temperature for 2 h and concentrated under reduced pressure. The resulting residue was purified by semi-prep TLC (CH_2_Cl_2_/MeOH/H_2_O, 80:20:3) to give click adduct **1** as a yellow amorphous solid (25 mg, 84%). R_*f*_ 0.62 (CH_2_Cl_2_/MeOH/H_2_O, 10:8:2); ^1^H NMR (400 MHz, CD_3_OD) δ 8.23 (1H, s, H-f), 8.17 (1H, s, H-j), 7.36 (1H, d, *J*_c,d_ = 8.8 Hz, H-c), 6.60 (1H, dd, *J*_c,d_ = 8.8, *J*_d,e_ = 2.2 Hz, H-d), 6.46 (d, *J*_d,e_ = 2.2 Hz, H-e), 4.64 (1H, d *J*_1,2_ = 1.5 Hz, H-1), 3.76–3.67 (3H, m, H-2, H-6a, OC*H*_*a*_H_b_), 3.62–3.59 (2H, m, H-3, H-6b), 3.51–3.35 (3H, m, H-4, H-5, OCH_a_*H*_*b*_), 2.72 (2H, t, *J* = 7.5 Hz, OCH_2_CH_2_CH_2_C*H*_*2*_), 1.78–1.71 (2H, m, OCH_2_CH_2_C*H*_2_CH_*2*_), 1.65–1.57 (2H, m, OCH_2_C*H*_2_CH_2_CH_*2*_); ^13^C NMR (100.6 MHz, CD_3_OD): δ 159.3, 158.0, 138.5131.3, 124.2, 119.5, 104.7, 101.4 (C-1), 74.7 (C-5), 72.6 (C-3), 72.3 (C-2), 68.7 (C-4), 68.2 (C-6), 30.0, 27.3, 26.0; HRMS (ESI) *m/z* calcd for C_21_H_25_N_3_O_9_Na^+^ ([M+Na]^+^): 486.1483, found 486.1476.

#### 4.2.7 4-(1-(7-Hydroxy-coumarin-3-yl)-1H-1,2,3-triazol-4-yl)-)butyl α-d-mannopyranosyl-(1 → 6)-α-d-mannopyranoside (**2**)

A solution of hexynyl disaccharide **9** (15 mg, 0.04 mmol) and 3-azido-7-hydroxycoumarin (**10**) (7.2 mg, 0.04 mmol) in MeOH/H_2_O (1:1) (1 mL) was treated with 1 M aq. CuSO_4_ (10 μL) and 1 M aq. NaAsc (25 μL). The reaction mixture was allowed to stir at room temperature for 2 h and concentrated under reduced pressure. The resulting residue was purified by semi-prep TLC (CH_2_Cl_2_/MeOH/H_2_O, 80:20:3) to give click adduct **2** as a yellow amorphous solid (20 mg, 90%). R_*f*_ 0.37 (CH_2_Cl_2_/MeOH/H_2_O, 10:8:2); ^1^H NMR (400 MHz, CD_3_OD): δ 8.37 (1H, s, H-f), 8.25 (1H, s, H-j), 7.52 (1H, d, *J*_c,d_ = 8.7 Hz, H-c), 6.78 (1H, dd, *J*_c,d_ = 8.7, *J*_d,e_ = 2.3 Hz, H-d), 6.71 (1H, d, *J*_d,e_ = 2.3 Hz, H-e), 4.72 (1H, d, *J*_1′,2′_ = 1.7 Hz, H-1′), 4.63 (1H, d, *J*_1,2_ = 1.5 Hz, H-1), 3.81–3.50 (14H, m, H-6a, H-6b, H-2′, H-2; H-3, H-3′, OC*H*_*a*_H_b_, H-6′a, H-6′b, H-5′, H-5, H-4′, H-4), 3.41–3.36 (1H, m, OCH_a_*H*_*b*_), 2.73 (2H, t, *J* = 7.5 Hz, OCH_2_CH_2_CH_2_C*H*_*2*_), 1.77–1.70 (2H, m, OCH_2_CH_2_C*H*_2_CH_*2*_), 1.64–1.56 (2H, m, OCH_2_C*H*_2_CH_2_CH_*2*_); ^13^C NMR (100.6 MHz, D_2_O): δ 157.8, 156.8, 138.6, 131.8, 124.3, 115.9, 103.4, 101.6 (C-1), 101.3 (C-1′), 74.8, 74.4, 73.2, 72.8, 72.6, 72.2, 68.7 (H-4, H-4′), 68.6 (O*C*H_a_H_b_), 68.2 (C-6), 67.5, 62.9 (C-6′), 30.0, 27.4, 26.0; HRMS (ESI) *m/z* calcd for C_27_H_35_N_3_O_14_Na^+^ ([M+Na]^+^): 648.2011, found 648.2002.

#### 4.2.8 4-(1-(7-Hydroxy-coumarin-3-yl)-1H-1,2,3-triazol-4-yl)-)butan-1-ol (**11**)

A solution of 5-hexynol (25 mg, 0.1 mmol) and 3-azido-7-hydroxycoumarin (19 mg, 0.1 mmol) in MeOH (0.8 mL) was treated with 1 M aq. CuSO_4_ (10 μL) and 1 M aq. sodium ascorbate (25 μL). The reaction mixture was allowed to stir at room temperature for 2 h and concentrated under reduced pressure. The resulting residue was purified by semi-prep TLC (CH_2_Cl_2_/MeOH/H_2_O, 80:20:3) to give click adduct **2.11** as a yellow amorphous solid (29 mg, 70%). R_*f*_ 0.84 (CH_2_Cl_2_/MeOH/H_2_O, 10:8:2); ^1^H NMR (400 MHz, MeOD): δ 8.37 (1H, s, H-f), 8.24 (1H, s, H-j), 7.54 (1H, d, *J*_c,d_ = 8.6 Hz, H-c), 6.80 (1H, dd, *J*_c,d_ = 8.6, *J*_d,e_ = 2.3 Hz, H-d), 6.72 (d, *J*_d,e_ = 2.3 Hz, H-e), 3.51 (2H, t, *J* = 6.5 Hz, C*H*_*2*_-triazole), 2.72 (2H, t, *J* = 7.5 Hz, HO—C*H*_*2*_), 1.74–1.66 (2H, m, CH_2_), 1.56–1.49 (2H, m, CH_2_); ^13^C NMR (100.6 MHz, CD_3_OD): δ 137.1, 131.9, 122.3, 115.6, 103.4, 33.1, 26.9, 26.0; HRMS (ESI) m/z calcd for C_15_H_16_N_3_O_4_^+^ ([M+H]^+^): 302.1135 found 302.1139.

### 4.3 Preparation of *Euglena gracilis* cells

As described previously [7], an axenic culture of *Euglena gracilis* var *saccharophila* Klebs (strain 1224/7a), was grown in the dark at 30 °C, with shaking (200 rpm), in modified *Euglena gracilis* plus Jaworski's medium (1 × EG plus 1 × JM medium) supplemented with glucose (15 g/l) for 7 days (refer to the Supplementary Information for full details). Dark grown culture after seven days was harvested by centrifugation (800 g for 5 min), washed twice with deionised water and once in HEPES buffer (10 mM, pH 7.0 HEPES/KOH, 25 mM KCl). The harvested cells were re-suspended in HEPES buffer and de-flagellated by the method of cold-shock [30] that required incubation on ice for 2 h and the collection of de-flagellated cells by centrifugation (800 g for 5 min).

### 4.4 Isolation of microsomal membranes from *Euglena gracilis*

The isolation of *Euglena* microsomal membranes was conducted according to published procedures [22]. All isolation steps described were carried out at 0–4 °C. Dark-grown de-flagellated cells were re-suspended in *Euglena* lysis buffer [20 mM, pH 7.0 HEPES/KOH, 50 mM NaCl, protease inhibitors (1 tablet per 100 mL) and ribonuclease A (1 mg)] and disrupted by three 15 s bursts of ultrasonic waves over three consecutive cycles. Residual cell debris was removed by centrifugation (300 g for 3 min) and supernatant was centrifuged at 4200 g for 30 min to remove mitochondria. The supernatant was layered onto a 1.5 M sucrose cushion in 20 mM HEPES buffer (pH 7.0) and centrifuged at 15,000 g for 45 min. The supernatant was removed and layered over a step gradient consisted of 1.3 M, 1.0 M, 0.8 M and 0.5 M sucrose in 20 mM HEPES buffer (pH 7.0). The separation of microsomal fraction was achieved by centrifugation at 150,000 g for 3 h. Microsomal-enriched fractions were collected, re-suspended in 20 mM HEPES buffer (pH 7.0) and centrifuged at 150,000 g for 2 h. The pellets were re-homogenised in 20 mM HEPES buffer (pH 7.0) and stored at −80 °C; under these conditions membrane enzymes remained active for up to a year.

### 4.5 Fluorescence-based assays to probe glycosyltransferase activities in *Euglena gracilis*

Assays were initiated by the addition of 150 μL of freshly thawed *E. gracilis* microsomal membranes (195 μg protein) to a solution of 4 mM donor (GDP-d-Man, GDP-l-Fuc, UDP-d-Glc, or UDP-d-GlcNAc) and 2 mM acceptor (**1** or **2**) in 50 μL reaction buffer (40 mM HEPES/KOH, 100 mM KCl, 40 mM MgCl_2_, 40 mM MnCl_2_, 40% glycerol, pH 7.0). After incubation for 24 h at 30 °C reactions were stopped by addition of CHCl_3_:MeOH (1:1, v/v), microsomal membranes were centrifuged (16,000 g for 5 min), then repeatedly washed with CHCl_3_:MeOH:H_2_O (10:6:1) and centrifuged. Combined supernatants were dried under a gentle stream of air. The residue was re-dissolved in deionised water and passed through 0.45 μm PPTE filter, the filtrate was collected and the sample was freeze-dried. Control assays without donor were performed in parallel in the same conditions.

### 4.6 Exo-glycosidase digestion, phosphate ester and phosphodiester linkage analysis

Jack bean α-mannosidase and green coffee beans α-galactosidase were obtained from Sigma Aldrich, β-galactosidase from Calbiochem, *Xanthomonas manihotis* α-1,6-mannosidase from New England Biolabs and *Aspergillus saitoi* α-1,2-mannosidase from Prozyme. Protocols for their use can be found in the SI, along with details of acid and phosphatase reactions used to assess phosphate esters/diesters.

### 4.7 Enzymatic synthesis of 4-(1-(7-hydroxy-coumarin-3-yl)-1H-1,2,3-triazol-4-yl)-butyl 6-(2-acetamido-2-deoxy-α-d-glucopyranosyl hydrogen phosphate)-α-d-mannopyranosyl-(1 → 6)-α-d-mannopyranoside (**19**)

A large scale reaction was performed to generate sufficient material for structural characterisation by NMR spectroscopy. In this enzymatic transformation acceptor **19** (0.8 mg, 1.28 μ mol) and donor (UDP-GlcNAc) (3.2 mg, 4.9 μmol) were dried out and re-dissolved in 150 μL of stock reaction buffer (40 mM, pH 7.0 HEPES/KOH, 100 mM KCl, 40 mM MgCl_2_, 40 mM MnCl_2_, 40% glycerol) followed by the addition of 450 μL of *E. gracilis* microsomal membranes (585 μg protein) in a total volume of 600 μL. After incubation for 24 h at 30 °C the reaction was supplemented with another portion of *E. gracilis* microsomal membranes (150 μL, 195 μg of membrane proteins) and incubated for a further 24 h at 30 °C. The reaction was stopped by addition of CHCl_3_/MeOH (1 mL). The denatured *E. gracilis* microsomal membranes were removed by several centrifugations (14,000 rpm for 5 min) and washed with CHCl_3_/MeOH:H_2_O (10:6:1) (3 × 1 mL). The washings were combined and solvents dried under gentle stream of air. The residue was re-dissolved in deionised water and passed through a 0.45 μm PPTE filter, the filtrate was collected and the sample was freeze-dried. The residue was then purified by reverse phase HPLC to give compound **20** (0.48 mg, 43%). ^1^H NMR (800 MHz, D_2_O): δ 8.43 (1H, s, H-f), 8.26 (1H, s, H-j), 7.73 (1H, d, *J*_c,d_ = 8.6 Hz, H-c), 7.06 (1H, dd, *J*_c,d_ = 8.6, *J*_d,e_ = 2.2 Hz, H-d), 7.01 (bd, H-e), 5.48 (1H, dd *J*_1,2_ = 3.6 Hz, *J*_1-P_ = 7.2 Hz, H-1), 4.13 (2H, m, H-6a′ and H-6b′), 3.95 (4H, m, H-2″, H-6a, H-6b, H-2′), 3.83–3.54 (m, sugar signals, OC*H*_*a*_H_b_, OCH_a_*H*_*b*_), 2.86 (2H, t, *J*_H-H_ = 7.2 Hz, OCH_2_CH_2_CH_2_C*H*_*2*_), 2.07 (3H, s, CH_3_), 1.87–1.78 (2H, m, OCH_2_CH_2_C*H*_2_CH_*2*_), 1.72–1.70 (2H, m, OCH_2_C*H*_2_CH_2_CH_*2*_); ^31^P NMR (100.6 MHz, D_2_O): δ 1.23; HRMS (ESI) m/z calcd for C_35_H_50_N_4_O_22_P^+^ ([M+H]^+^): calcd 909.2649, found, 909.2648.

## Footnotes

2 The efficiency of all three CuAAC reactions was affected by the formation of traces of by-products with lower R_*f*_ values; the identities of these by-products were not pursued.
