# Supplementary material for: Fluorescent mannosides serve as acceptor substrates for glycosyltransferase and sugar-1-phosphate transferase activities in Euglena gracilis membranes
Source: Carbohydr Res. 2017 Jan 13;438:26–38. doi: 10.1016/j.carres.2016.11.017 (PMC5240791; doi:10.1016/j.carres.2016.11.017)
Supplement: Supplementary file 1 [file mmc1.pdf]

Supplementary Information

Fluorescent mannosides serve as acceptor substrates for glycosyltransferase and sugar-1-phosphate transferase activities in *Euglena gracilis* membranes

Irina M. Ivanova, Sergey A. Nepogodiev, Gerhard Saalbach, Ellis C. O'Neill, Michael D. Urbaniak, Michael A. J. Ferguson, Sudagar S. Gurcha, Gurdyal S. Besra and Robert A. Field*

Table of Contents

1 Examination of detection limits of fluorescently labelled coumarinyl α-D-mannopyranoside derivatives 4
1.1 TLC analyses 4
1.2 Solution analyses 4
2 Benchmarking fluorescence-based assay against radiochemical assay used in Mycobacterium smegmatis 5
2.1 Fluorescence-based assay to probe α-1,6-mannosyltransferase activity in Mycobacterium smegmatis 5
2.2 TLC results from the fluorescence-based assay 6
2.3 Identification of fluorescent products by LC-MS 6
2.4 Linkage analysis through exo-mannosidase digestions 7
3 Benchmarking fluorescence-based assay against radiochemical assay used in *Trypanosoma brucei* 8
3.1 Fluorescence-based assay to probe galactosyltransferase activities responsible for α-galactosylation of GPI anchors in Trypanosoma brucei 8
3.2 TLC results from the fluorescence-based assay 9
3.3 Identification of fluorescent products by LC-MS 10
3.4 Linkage analysis through exo-galactosidase digestions 11
4 Identification of fluorescent products 12 and 13 by LC-MS 12
5 Linkage analysis through *exo*-mannosidase digestions of product 12 and 13 13
5.1 Confirmation of newly formed linkages in fluorescent mannoside products by LC-MS 16
6 Investigation of newly formed linkages in fluorescent products 15, 16, 18 and 19 through enzymatic and chemical degradations 19
7 Characterisation of fluorescent product 19 by NMR spectroscopy 21
8 MS data and elemental composition for compound 19 22

8.1 Elemental Composition Report 22
9 Experimental........ 23
9.1 3-Azido-7-hydroxy-2H-crhomen-2-one 23
9.2 General methods and materials for the biotransformations 23
9.2.1 Hydrolytic enzymes and procedures used for characterisation of biotransformation products... 24
9.3 Fluorescence-based assays to probe mannosyltransferase activities in Mycobacterium smegmatis25
9.4 Fluorescence-based assays to probe galactosyltransferase activities in Trypanosoma brucei 25
9.4.1 Variations of enzyme concentration 26
9.4.2 Increasing donor concentration 26
9.5 Euglena gracilis growth medium composition 26
10 NMR data 28
11 References: 43

List of Figures

Fig. S1 TLC detection range of fluorescently labelled α-Man-1,6-α-Man-HCT (2). 4

Fig. S2 The detection range of fluorescently labelled α-Man-1,6-α-Man-HCT (2) in solution. 5

Fig. S3 TLC analyses of enzymatic assays involving incubation of α-Man-1,6-α-Man-HCT (2) fluorescent acceptor substrate with GDP-Man to detect mannosyltransferase activity in *Mycobacterium smegmatis*. 6

Fig. S4 LC-MS analyses of reaction mixture obtained from incubation of α-Man-1,6-α-Man-HCT (2) with GDP-Man in the presence of *Mycobacterium smegmatis* microsomal membranes. 7

Fig. S5 TLC analyses of *exo*-mannosidase digestions of mixed fluorescent products obtained from α-Man-1,6-α-Man-HCT (2). 8

Fig. S6 TLC analyses of enzymatic assay of fluorescent α-Man-1,6-α-Man-HCT (2) acceptor used in detection of galactosyltransferase activities in *Trypanosoma brucei* membranes. 9

Fig. S7 TLC analyses of enzymatic assays of fluorescent α-Man-1,6-α-Man-HCT (2) acceptor to improve galactosyltransferase activities in *Trypanosoma brucei* microsomal membranes. 10

Fig. S8 UPLC analyses of reaction mixture obtained from incubation of fluorescent α-Man-1,6-α-Man-HCT (**2**) acceptor with UDP-Gal in the presence of *Trypanosoma brucei* microsomal membranes. 11

Fig. S9 Analyses of *exo*-glycosidase digestion of fluorescent products with α-galactosidase from green coffee beans and β-galactosidase from *Escherichia coli*. 12

Fig. S10 Structural identification of fluorescent products obtained from enzymatic reactions with GDP-Man and *Euglena gracilis* microsomal membranes by LC-MS. 13

Fig. S11 TLC analyses to assess stereoselectivity and regioselectivity of disaccharide fluorescent products by *exo*-glycosidase digestions. 15

Fig. S12 TLC analyses to assess stereoselectivity and regioselectivity of trisaccharide fluorescent products by *exo*-glycosidaseF digestions. 16

Fig. S13 Formation of two fluorescent disaccharide isomeric compounds confirmed by LC-MS analysis. .. 17

Fig. S14 Presence of one fluorescent trisaccharide 13 compound confirmed by LC-MS analysis. 18

Fig. S15 TLC analyses to investigate presence of terminal phosphate in fluorescent products through application of enzymatic hydrolysis with alkaline phosphatase. 19

Fig. S16 TLC analyses to investigate the presence of phosphodiester bond in fluorescent products through application of chemical degradation and enzymatic hydrolysis. 20

Fig. S17 Diagrammatic representation of sequential chemical and enzymatic degradation of fluorescent disaccharide and trisaccharide products containing phosphodiester bond. 20

Fig. S18 Selected regions of ^{1}H NMR (800 MHz, D_2O, 25 °C) spectra of compound **19**. 21

Fig. S19 High resolution MS spectrum of compound **19** 22

List of tables

Table 1 Calculated elemental composition for compound 19 22

Table 2 EG:JM medium 27

1 Examination of detection limits of fluorescently labelled coumarinyl α-D-mannopyranoside derivatives

1.1 TLC analyses

The lowest amount of fluorescent α-Man-1,6-α-Man- HCT (**2**) compound that could be detected on the TLC plates using UV-light were determined in the series of experiments. For this purpose, aqueous solution of **2** in a wide range of concentrations was prepared, and 2 μL aliquots were loaded onto a glass-backed TLC plate containing no fluorescent indicator. The TLC plate was eluted with CH_2Cl_2:MeOH (9:1) solvent system and then visualised with a mid-wave length UV light for fluorescence detection (Fig. S1A) and then stained with orcinol for standard carbohydrate detection (Fig. S1B).[1] The results indicated that the fluorescent method of detection for **2** is ca. 100 fold more sensitive than orcinol staining, allowing detection of fluorescent mannoside acceptor at 25 ng or 40 pmols for compound **2**. Value obtained with fluorescent method of detection is lower compared to a typical limits of detection using radiolabelling.

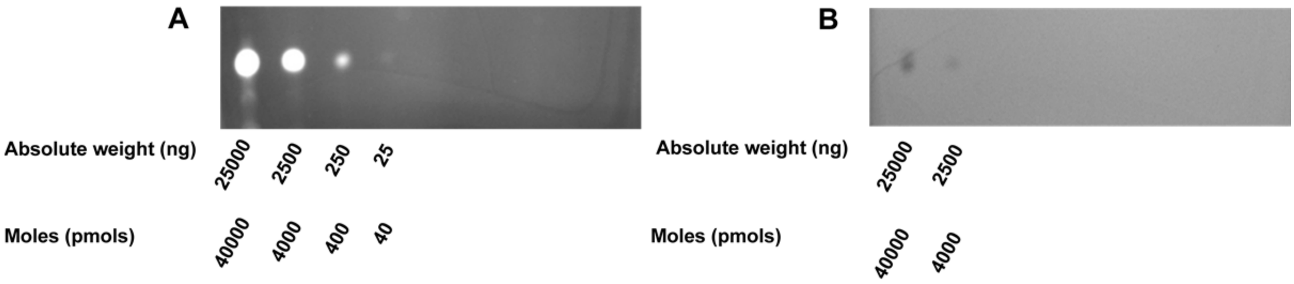

Fig. S1 TLC detection range of fluorescently labelled α-Man-1,6-α-Man-HCT (2).
(A) The TLC plate visualised with mid-wave length UV light (B) The TLC plate developed with orcinol staining. TLC conditions: 2 μL of 2 $\times 10^{-2}$ M, 2 $\times 10^{-3}$ M, 2 $\times 10^{-4}$ M, 2 $\times 10^{-5}$ M aliquots in water were loaded onto the TLC and eluted with (CH_2Cl_2:MeOH:H_2O (80:20:3)).

1.2 Solution analyses

In order to achieve quantification of fluorescently labelled glycoside products obtained from the enzymatic biotransformation the detection limits of α-Man-1,6-α-Man-HCT (**2**) were examined in solution. Again a range of concentrations were prepared at the pH level with the highest fluorescence intensity observed, for **2** at pH 9 in universal buffer (0.04 M H_3BO_3, H_3PO_4, and CH_3COOH). The fluorescence intensity was measured as the concentration of each compound decreased (Fig. S2). The analysis of results showed that concentrations as low as 0.06 μM could be detected using a fluorometer.

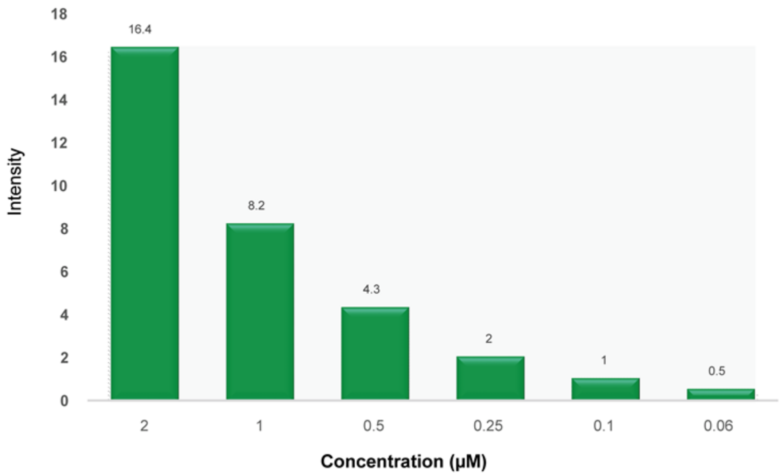

Fig. S2 The detection range of fluorescently labelled α-Man-1,6-α-Man-HCT (2) in solution.
(■) Solution of **2** at pH 9 (universal buffer), Ex_{max} 344 nm, Em_{max} 469 nm.

2 Benchmarking fluorescence-based assay against radiochemical assay used in Mycobacterium smegmatis

2.1 Fluorescence-based assay to probe α-1,6-mannosyltransferase activity in Mycobacterium smegmatis

In order to understand the biosynthesis of lipoarabinomannan, cell-free radiolabelled assays were developed by Dr. Brown and used in Professor Besra's group to study the biosynthetic pathways and substrate specificities of these glycosyltransferases in mycobacterial species.[2,3] One of these radiolabelled assay that looked at the substrate activities of chemically synthesised substrates for ManT responsible for elongation of LM structure was used to benchmark our fluorescence-based methodologies.[87] In their study, chemically synthesised compounds, such as octyl 6-*O*-α-D-manopyranosyl-α-D-mannopyranoside and octyl 6-*O*-α-D-manopyranosyl-1-thio-α-D-mannopyranoside were designed to mimic LM intermediates and to target α-1,6-ManT involved in the elongation of α-1,6-linked LM. During these assays acceptor substrates were incubated with the radiolabelled GDP-[^{14}C]Man in the presence of *M. smegmatis* membranes. [*M. smegmatis* is a fast growing non-pathogenic bacterium that has a similar cell wall composition to *M. tuberculosis* and is used as a model organism.]

The reported results demonstrated that both compounds acted as acceptor substrates for these enzymes, leading to formation of two radiolabelled mannoside products, as evaluated by HPTLC and autoradiography. The nature of α-1,6-glycosidic linkage in these assays has been confirmed through *exo*-glycosidase digestion and permethylation analysis by GC-MS, which indicated formation of a major α-1,6-linked trisaccharide and minor α-1,6-linked tetrasaccharide radiolabelled products. Therefore, in order to benchmark our fluorescence-based methodologies we were looking to obtain similar results.

2.2 TLC results from the fluorescence-based assay

Mycobacterial membranes were obtained by growing *M. smegmatis* (strain mc^2155) in media (Bacto Nutrient Broth) to mid-log phase (about 24 h). The harvested cells were then lysed, centrifuged to remove larger cell particles and ultracentrifuged to obtain the required mycobacterial membranes.[4] Fluorescent α-Man-1,6-α-Man-HCT (**2**) was incubated with GDP-Man in the presence of washed mycobacterial membranes under the same conditions used in the radiolabelled assays. The progress of each enzymatic reaction was monitored by TLC.

During enzymatic assays 20 µL aliquots at 2 h, 4 h, 6 h, and 29 h were taken and stopped by addition of methanol-chloroform (1:1) and visualised on the TLC plate. TLC analysis of the enzymatic reaction from α-Man-1,6-α-Man-HCT (**2**) acceptor showed formation of two new fluorescent bands (Fig. S3). The most intense band below the starting acceptor was tentatively assigned to trisaccharide product and the less intense band with the lower R_f value was assigned as tetrasaccharide product. Fluorescent bands with higher R_f value indicated a possible hydrolysis of α-Man-1,6-α-Man-HCT (**2**).

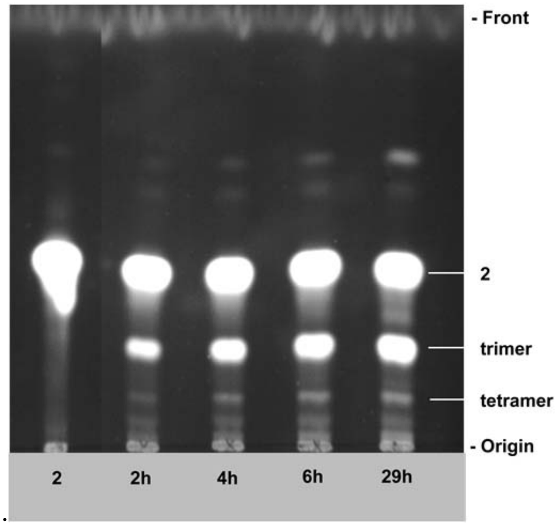

Fig. S3 TLC analyses of enzymatic assays involving incubation of α-Man-1,6-α-Man-HCT (2) fluorescent acceptor substrate with GDP-Man to detect mannosyltransferase activity in *Mycobacterium smegmatis*.
Assay conditions: acceptor (1 mM) and GDP-Man (4 mM) were incubated for 29 h at 37°C in MOPS/KOH (50 mM, pH 7.9) buffer supplemented with $MgCl_2$ (10 mM), DTT (5 mM) in the presence of *M. smegmatis* microsomal membranes (250 µL, 4000 µg of total protein) in total reaction volume of 1 mL. TLC plate was eluted with $CHCl_3$:MeOH:H_2O (10:6:1) visualised using mid-wave length range UV light. Reaction time points are shown below the TLC image and acceptor and products are shown to the side of TLC plate

2.3 Identification of fluorescent products by LC-MS

In order to confirm formation of fluorescent mannoside products the reaction mixture after 29 h of incubation was submitted to the LC-MS analysis. Normal phase LC coupled with UV-detection allowed separation of fluorescent products revealing two peaks at retention time 7.2 min and 8.0 min for the reaction mixture involving α-Man-1,6-α-Man-HCT (**2**) (Fig. S4).

The analysis of MS data of fluorescent trimer confirmed addition of single mannose residue to α-Man-1,6-α-Man-HCT (**2**), thus major signal observed in both MS spectrum had *m/z* of 788.25, respectively, corresponding to $[M+H]^+$ of a trisaccharide molecular ion (Fig. S4 B1a). The MS data for fluorescent tetramer confirmed addition of two mannose residue to α-Man-1,6-α-Man-HCT (**2**) with *m/z* of 950.13 corresponding to $[M+H]^+$ of a tetrasaccharide molecular ion (Fig. S4 B2b). LC-MS data were in agreement with the preliminary TLC observations.

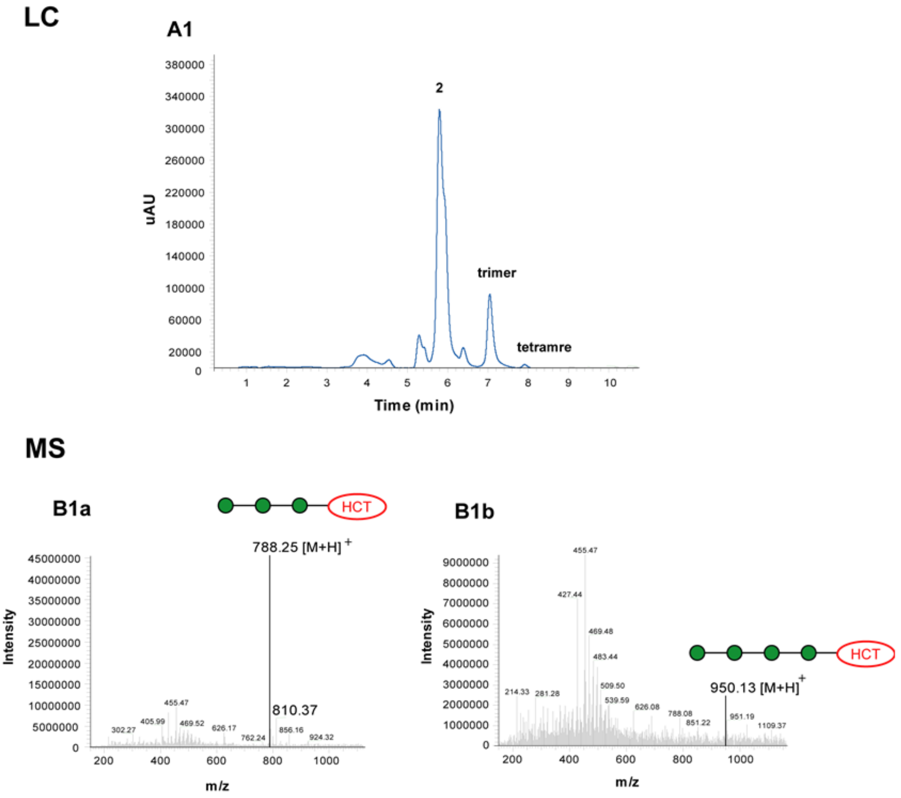

Fig. S4 LC-MS analyses of reaction mixture obtained from incubation of α-Man-1,6-α-Man-HCT (2) with GDP-Man in the presence of *Mycobacterium smegmatis* microsomal membranes.
(A1) UV chromatogram. LC conditions: Kinetex LUNA NH_2 (100 mm/2.0 mm/3 µm), mobile phase: CH_3CN-water (0.1% TFA) (10 to 90 %, 32 min, flow rate: 1 mL/min) UV detector at 347 nm. (B1) MS spectra (B1a) trimer; (B1b) tetramer.

2.4 Linkage analysis through exo-mannosidase digestions

The newly formed linkages in fluorescent trimer and tetramer were assessed through the application of *exo*-mannosidase digestion with a hydrolase of well-defined α-1,6-specificity. For this purpose, both enzymatic reactions were scaled up to obtain sufficient amount of fluorescent products to be analysed. The purification of fluorescent trimer and tetramer from α-Man-1,6-α-Man-HCT (**2**) was attempted by a normal phase HPLC method. Disappointingly, the obtained fractions contained mixed products as some interactions of coumarin-based fluorophore with the amino column were observed, which resulted in tailing of peaks for all fluorescent compounds. HPLC purification of the reaction mixture from α-Man-1,6-α-Man-HCT (**2**) gave two mixed fractions. The first semi-purified fraction contained mixed trisaccharide product, a faint amount

of α-Man-1,6-α-Man-HCT (**2**) and product of hydrolysis α-Man-HCT (**1**) (Fig. S5, lane 1). The second semi-purified fraction contained tetrasaccharide product, small amount of trisaccharide as well as **2** and **1** (Fig. S5, lane 3). Both fractions were subjected to *exo*-mannosidase digestion with *Xanthomonas manihotis* α-1,6-mannosidase (Fig. S5, lane 2 and 4). TLC analysis indicated that hydrolysis of trisaccharide and tetrasaccharide products with α-1,6-mannosidase resulted in formation of α-Man-HCT (**1**). The obtained data is in agreement with the data obtained from the radiolableed assays and also indicates that fluorescent α-Man-1,6-α-Man-HCT (**2**) can act as an alternative acceptor substrate for α-1,6 ManT present in *M. smegmatis* membranes.

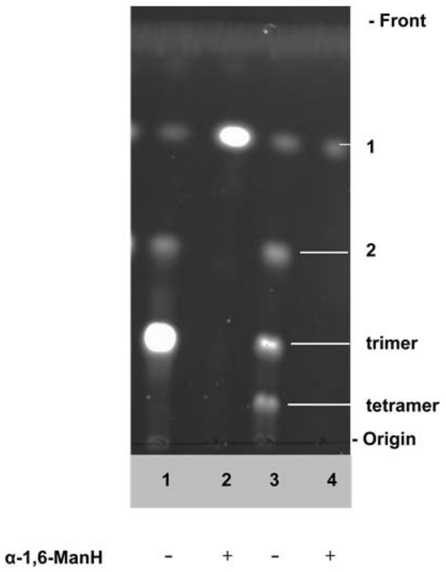

Fig. S5 TLC analyses of *exo*-mannosidase digestions of mixed fluorescent products obtained from α-Man-1,6-α-Man-HCT (2).
Enzyme used in this assay is *Xanthomonas manihotis* α-1,6-mannosidase (α-1,6-ManH). TLC plates were eluted with $CHCl_3$:MeOH:H_2O (10:8:1) and visualised using mid-wave length range UV light. Starting acceptor and products are shown to the right of TLC image.

3 Benchmarking fluorescence-based assay against radiochemical assay used in *Trypanosoma brucei*

3.1 Fluorescence-based assay to probe galactosyltransferase activities responsible for α-galactosylation of GPI anchors in Trypanosoma brucei

Results from a study that investigated substrate specificity of chemically synthesised compounds for galactosyltransferases (GalT) responsible for the decoration of GPI anchor were used to further validate and benchmark our fluorescence-based assay.[5] In the published radiolabelled assays, three acceptor substrates, namely octyl 6-*O*-α-D-manopyranosyl-1-thio-α-D-mannopyranoside, octyl thio-α-D-mannopyranoside and octyl thio-β-D-mannopyranoside were incubated with radiolabelled UDP-[^{3}H]Gal in the presence of isolated *T. brucei* membranes. The published results of these assays indicated that octyl 6-*O*-α-D-manopyranosyl-1-

thio-α-D-mannopyranoside, octyl thio-α-D-mannopyranoside and octyl thio-β-D-mannopyranoside were capable of accepting one galactose residue in either α- or β-configuration, with the α-linked products representing approximately 90, 40 and 10 %, respectively of these galactosylated products. The type of the glycosidic bond has been investigated in the case of only one acceptor, octyl 6-*O*-α-D-manopyranosyl-1-thio-α-D-mannopyranoside, because the main aim of that study was to detect α-GalT activity in *T. brucei* membranes. The type of glycosidic linkage in this assay has been determined by a combination of tandem negative-ion ESI MS and periodate oxidation followed by $NaBH_4$ reduction and indicated formation of a major product with α-1,3-linkage and two minor products with α-1,2 and α-1,4-linkages.

3.2 TLC results from the fluorescence-based assay

In this round of validation, the main aim was to demonstrate that the fluorescent α-Man-1,6-α-Man-HCT (**2**), already used in benchmarking against *M. smegmatis* radiolabelled assays can equally act as acceptor substrate for galactosyltransferases involved in the decoration of the core structure of GPI anchors in *T. brucei* microsomal membranes. The examined mannoside acceptor substrate **2** was incubated with UDP-Gal in the presence of trypanosome membranes under the same conditions as used in the literature radiolabelled assay.[5] Bloodstream *Trypanosoma brucei* (strain 427 variant MITat 1.4) were obtained from the infected rats[6] centrifuged, washed with ice-cold buffer and lysed using hypotonic buffer.[7] A cell-free lysate was then prepared as described in the literature.[5] TLC analysis showed formation of one fluorescent band with R_f value of expected trisaccharide product with a low conversion as judged by TLC (Fig. S6).

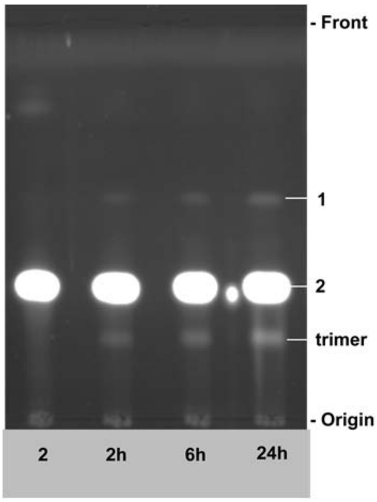

Fig. S6 TLC analyses of enzymatic assay of fluorescent α-Man-1,6-α-Man-HCT (2) acceptor used in detection of galactosyltransferase activities in *Trypanosoma brucei* membranes.

Assay conditions: acceptor substrate (1 mM) and UDP-Gal (1 mM) were incubated for 24 h at 30 °C in reaction buffer HEPES/KOH (100 mM, pH 7.4) supplemented with KCl (50 mM), $MgCl_2$ (10 mM) $MnCl_2$ (10 mM), DTT (2 mM), ATP (2 mM), *N*-tosyl-L-lysine chloromethyl ketone (0.2 mM), leupeptin (2 µg/mL), tunicamycin (2.5 µg/mL), Triton (0.05%) in the presence of *T. brucei* microsomal membranes (50 µL, 1×10^8 cells) in total reaction volume of 200 µL. TLC plates were eluted with $CHCl_3$:MeOH:H_2O (10:6:1) and visualised under UV light.

In order to increase amount of fluorescent product formed in the enzymatic reaction of α-Man-1,6-α-Man-HCT (**2**) with UDP-Gal in the presence of *T. brucei* microsomal membranes variation in two parameters were examined. Firstly, the reaction was carried out with five times the original amount of *T. brucei* microsomal membranes, and secondly with sequential increase of donor concentration during the enzymatic reaction time. The increase in the amount of *T. brucei* microsomal membranes resulted in little change in formation of trisaccharide product as followed from a similar fluorescent intensity when compared to previous TLC analyses (Fig. S6 vs Fig. S7 A). On the other hand, the increase in the amount of enzyme led to increase in hydrolysis of the starting acceptor resulting in increased fluorescent intensity of α-Man-HCT (**1**). The sequential addition of UDP-Gal donor resulted in an overall increase in acceptor substrate conversion as judged by the increase in intensity of fluorescent band of fluorescent trimer (Fig. S7 B).

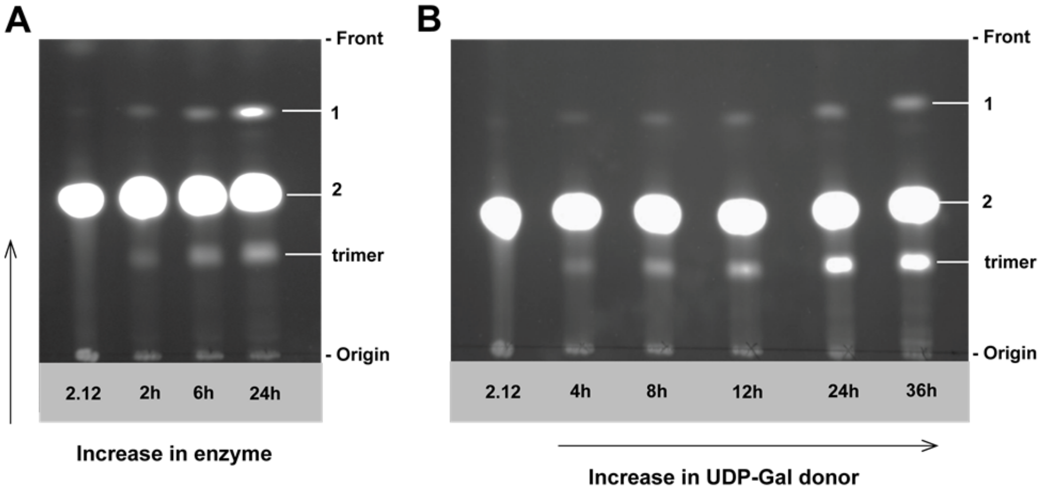

Fig. S7 TLC analyses of enzymatic assays of fluorescent α-Man-1,6-α-Man-HCT (2) acceptor to improve galactosyltransferase activities in *Trypanosoma brucei* microsomal membranes.
(A) The TLC plate features reaction mixture with addition of enzyme (25 µL $5{\times}10^8$ cells); (B) The TLC plate features reaction mixture with addition of UDP-Gal donor (5 µL of 4 mM at each time point). Initial assay conditions: acceptor substrate (1 mM), UDP-Gal (1 mM), reaction buffer HEPES/KOH (100 mM, pH 7.4), KCl (50 mM), $MgCl_2$ (10 mM), $MnCl_2$ (10 mM), DTT (2 mM), ATP (2 mM), *N*-tosyl-L-lysine chloromethyl ketone (0.2 mM), Leupeptin (2 µg/mL), Tunicamycin (2.5 µg/mL), Triton (0.05%); *T. brucei* microsomal membrane (25 µL, $1{\times}10^8$ cells); Total reaction volume of 100 µL. TLC plates were eluted with $CHCl_3$:MeOH:H_2O (10:6:1) and visualised under UV light. Reaction time points are shown below the corresponding TLC image and starting acceptor and product are shown to the left of the TLC image.

3.3 Identification of fluorescent products by LC-MS

In order to confirm formation of trisaccharide compound the reaction mixture from α-Man-1,6-α-Man-HCT (**2**) was subjected to LC-MS analysis. The ion-extracted chromatogram of *m/z* 788.29 indicated presence of two peaks that were designated as **trimer a** (retention time 6.14 min) and **trimer b** (6.23 min) (Fig. S8 A). The MS spectra of two peaks from the ion-extracted chromatogram had signal with *m/z* of 788.29 corresponded to a trisaccharide molecular ion $[M+H]^+$ thereby confirming the addition of a single galactose residue to **2** and formation of trisaccharide fluorescent products (**trimers a,b**). Other peaks present in MS spectra of **trimers a,b** with *m/z* of 626.23, 462.16 and 302.12 were assigned to the fragmentation of

molecular ion $[M+H]^+$ as a result of the sequential loss of hexose unit (*m/z* 162) and release of α-Man-1,6-α-Man-HCT (**2**), α-Man-HCT (**1**) and HCT (**11**), respectively (Fig. S8 B).

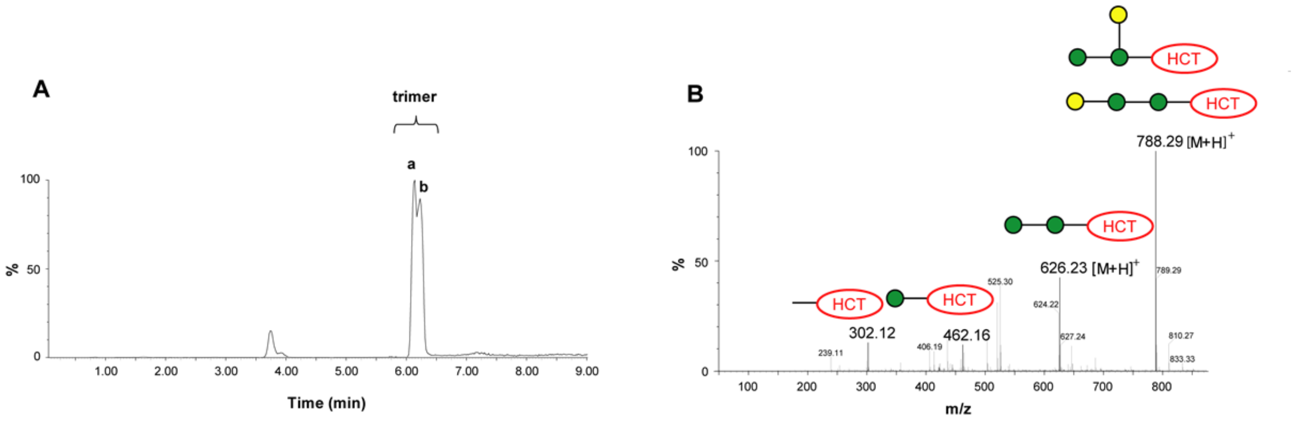

Fig. S8 UPLC analyses of reaction mixture obtained from incubation of fluorescent α-Man-1,6-α-Man-HCT (**2**) acceptor with UDP-Gal in the presence of *Trypanosoma brucei* microsomal membranes.
(A) Extracted ion chromatogram of 2.23. UPLC conditions: Acquity UPLC BEH C18 (100 mm/1.0 mm/1.7 µm), mobile phase: water- CH_3CN (0.1% formic acid) (10 to 90 %, 12 min, flow rate: 0.08 mL/min) MS detector. (B) MS spectrum of trimer a/b.

3.4 Linkage analysis through exo-galactosidase digestions

Assessment of the anomeric configuration of newly formed glycosidic linkages in trisaccharide was achieved through utilisation of enzymatic digestions with α-galactosidase from green coffee beans and β-galactosidase from *Escherichia coli*. The purification of trimer from starting **2** was attempted by a reverse phase HPLC method. The obtained fraction contained some starting material due to human error. The semi-purified fraction was subjected to *exo*-galactosidase digestion with α-galactosidase from green coffee beans and β-galactosidase from *E. coli* and the progress of both reactions were monitored by TLC.

TLC analysis of mixed fraction treated with α-galactosidase resulted in hydrolysis of significant amount of α-linked trisaccharide (**trimer a**) releasing fluorescent α-Man-1,6-α-Man-HCT (**2**) and leading to increase in fluorescent intensity of this compound compared to the intensity of control sample without the enzyme (Fig. S9 lane 1 and 2). In contrast, TLC analysis of mixed fraction with β-galactosidase indicate some hydrolysis of trimer (**trimer b**) compared to the control sample without enzyme (Fig. S9 lane 1 and 3). Moreover, there is a slight difference in R_f value of (**trimer a)** and (**trimer b**) where the R_f value of (**trimer a**) is slightly higher compared to the R_f value of (**trimer b**) (Fig. S9 lane 2 and 3).

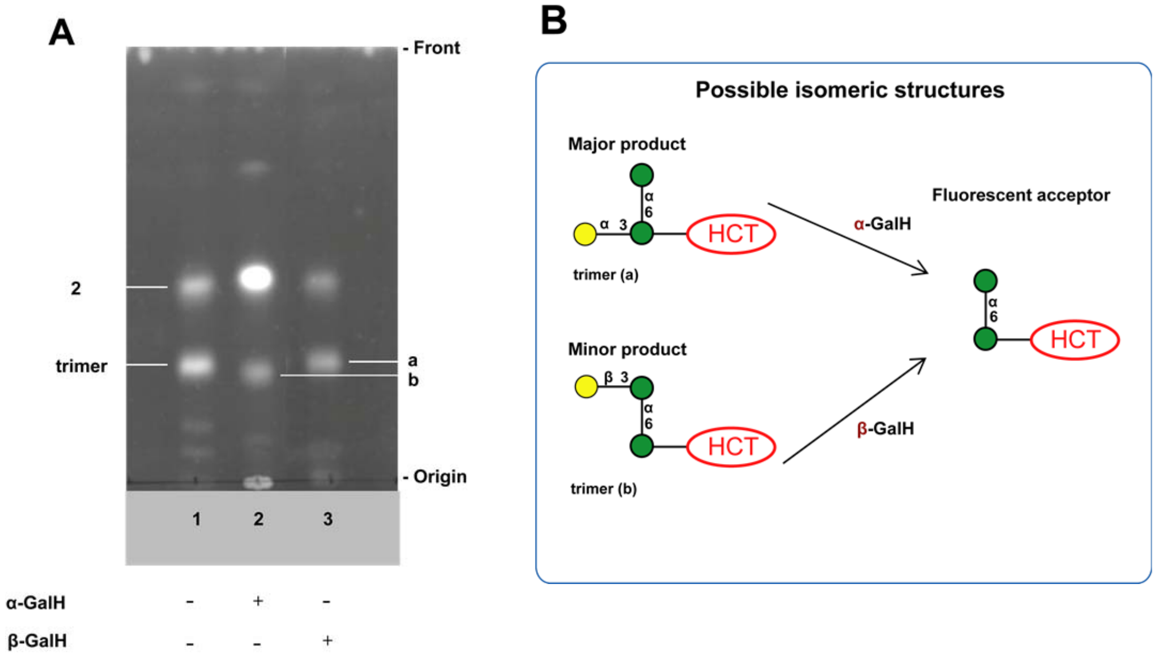

Fig. S9 Analyses of *exo*-glycosidase digestion of fluorescent products with α-galactosidase from green coffee beans and β-galactosidase from *Escherichia coli.*
(A) The TLC plate after exo-galactosidase digestion. Control experiment L1 (without enzyme) were conducted in parallel with hydrolysis reactions. The TLC plate were eluted with $CHCl_3$:MeOH:H_2O (10:6:1) and visualised using mid-wave length range UV light. The starting acceptor and products are shown to the left of the TLC image. (B) Schematic representation of degradation of two possible products by *exo*-galactosidases and release of fluorescent acceptor.

According to *exo*-galactosidase digestion data obtained from radiolabelled assays and the structural information of GPI anchor side chains in *T. brucei*, the major trisaccharide (**trimer a**) product hydrolysed by α-galactosidase was tentatively assigned to have an α-1,3-linkage to the reducing mannose of **2**. The minor (**trimer b**) product was tentatively assigned to have a β-1,3-linkage to the non-reducing mannose of **2** (Fig. S9 B).

4 Identification of fluorescent products 12 and 13 by LC-MS

Normal phase chromatographic conditions were used for the LC separation of purified disaccharide **12** and trisaccharide **13** compounds (Fig. S10, A1 red trace and SI 10, A2 blue trace). MS data of disaccharide **12a/b** (retention time 5.3 min) and trisaccharide **13a/b** (6.7 min) confirmed addition of single mannose residues to the α-Man-HCT (**1**) and α-Man-1,6-α-Man-HCT (**2**). Thus, major peaks observed in the MS spectrum of **12** (*m/z* 626.31) and **13** (*m/z* 788.16) correspond to $[M{+}H]^+$ of disaccharide and trisaccharide molecular ions (Fig. 10, B1 and B2), respectively. The MS2 fragmentation pattern of $[M{+}H]^+$ showed the sequential loss of hexose units (*m/z* 162) and ultimate release of fluorescent HCT (**11**) aglycone (*m/z* 302.00) in both MS2 spectra of **12** and **13** (Fig. 10, C1 and C2), respectively.

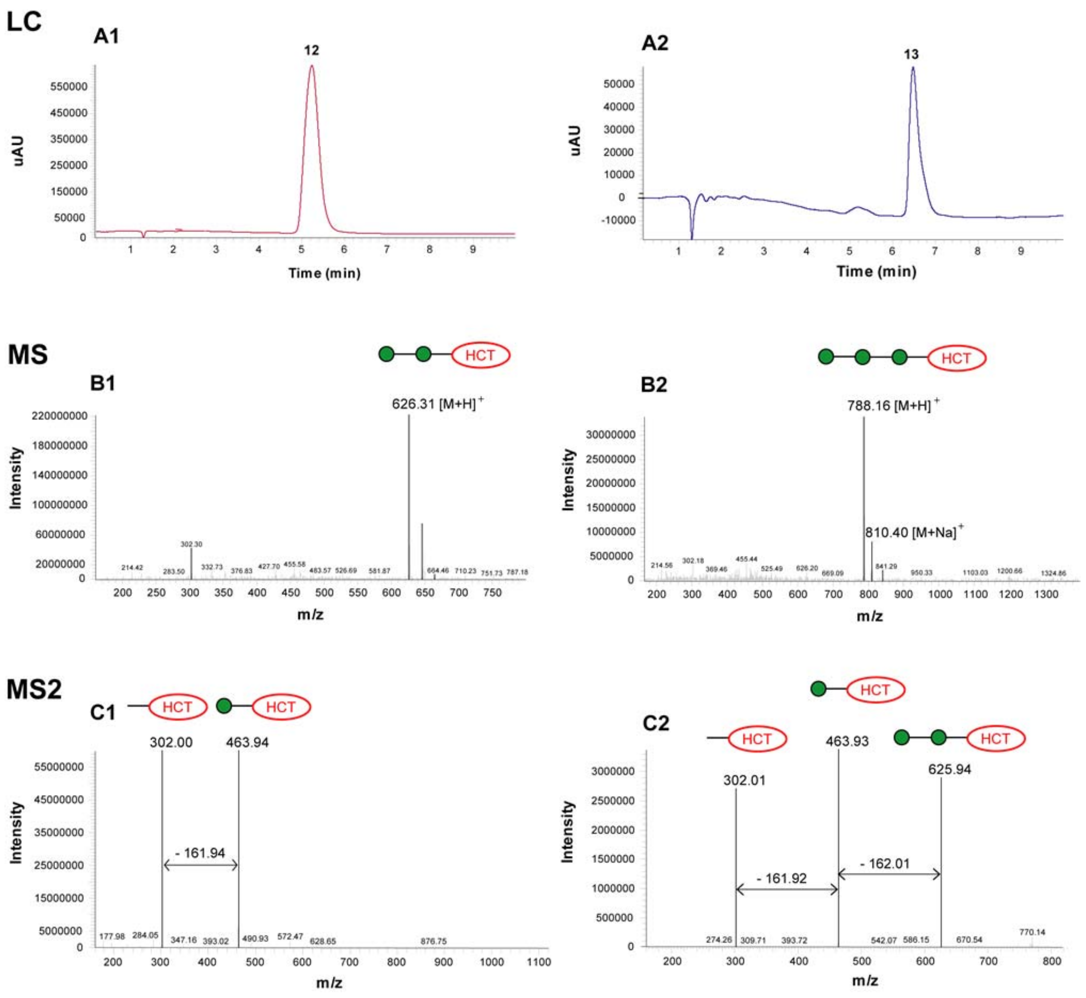

Fig. S10 Structural identification of fluorescent products obtained from enzymatic reactions with GDP-Man and *Euglena gracilis* microsomal membranes by LC-MS.
(A) UV chromatogram (A1) disaccharide **12** from α-Man-HCT (**1**) assay (red trace); (A2) trisaccharide **13** from α-Man-1,6-α-Man-HCT (**2**) assay (blue trace). LC conditions: Kinetex LUNA NH_2 (50 mm/2.1 mm/2.6 µm); mobile phase: CH_3CN- water (0.1% TFA) (10 to 90 %, 12 min, flow rate: 0.3 mL/min); UV detector at 347 nm. (B) MS spectra for **12** (B1) and **13** (B2). (C) MS2spectra of **12** (C1) and **13** (C2).

5 Linkage analysis through *exo*-mannosidase digestions of product 12 and 13

Information about the anomeric configuration of glycosidic linkages in disaccharide **12** and trisaccharide **13** were assessed through *exo*-glycosidase digestion with jack bean α-mannosidase.[8] Sequential digestion of **12** and **13** with three different linkage-specific *Aspergillus saitoi* α-1,2-,[9] *Xanthomonas manihotis* α-1,6- and *Xanthomonas manihotis* α-1,2/3-mannosidases,[10] capable of cleaving α-1,2-, α-1,6- and α-1,2/3-linked mannosides selectively, gave access to structural information on the regioselectivity of linkages formed between transferred mannose residues and α-Man-HCT (**1**) and α-Man-1,6-α-Man-HCT (**2**) acceptor substrates. Disaccharide **12** and trisaccharide **13** compounds were incubated with *exo*-glycosidase and the outcome of these reactions was analysed by TLC (Figs. SI 11 and 12).

TLC analyses of jack bean α-mannosidase digestion from disaccharide **12a/b** revealed formation of fluorescent band with the same R_f value as fluorescent HCT (**11**) aglycone, corresponding to removal of two mannose residues from **12** (Fig. S11A, lane 2 and Fig. S11B(i)). Incubation of disaccharide **12a/b** with *Xanthomonas manihotis* α-1,6-mannosidase had no effect on fluorescent compounds and therefore, formation of the α-1,6-linkage was excluded (Fig. S11A, lane 4). TLC analysis of disaccharide **12a/b** digestion with *Aspergillus saitoi* α-1,2-mannosidase released a significant amount of fluorescent product with the same R_f value as α-Man-HCT (**1**). However, a small amount of fluorescent disaccharide **12b** remained unhydrolysed, which is indicative of the presence of another isomeric compound with a different linkage (Fig. S11A, lane 6 and Fig. S11B(ii)). In order to assess what type of glycosidic linkage the underlying mannoside possessed, it was subjected to further digestion with *Xanthomonas manihotis* α-1,2/3-mannosidase. In this case, both fluorescent products were digested. The disaccharide **12a** was mainly converted into a fluorescent product with the same R_f value as HCT (**11**) aglycone, along with a minor fluorescent product with the same R_f as α-ManT-HCT (**1**) (Fig. S11A, lane 8 and Fig. S11B(iii)). Considering the 1,3-mannosidase activity of *Xanthomonas manihotis* α-1,2/3-mannosidase, it is reasonable to suggest that the mannose residue was attached to the fluorescent acceptor substrate α-ManT-HCT (**1**) mainly through α-1,2-linkage and in a minor amount through α-1,3-linkage.

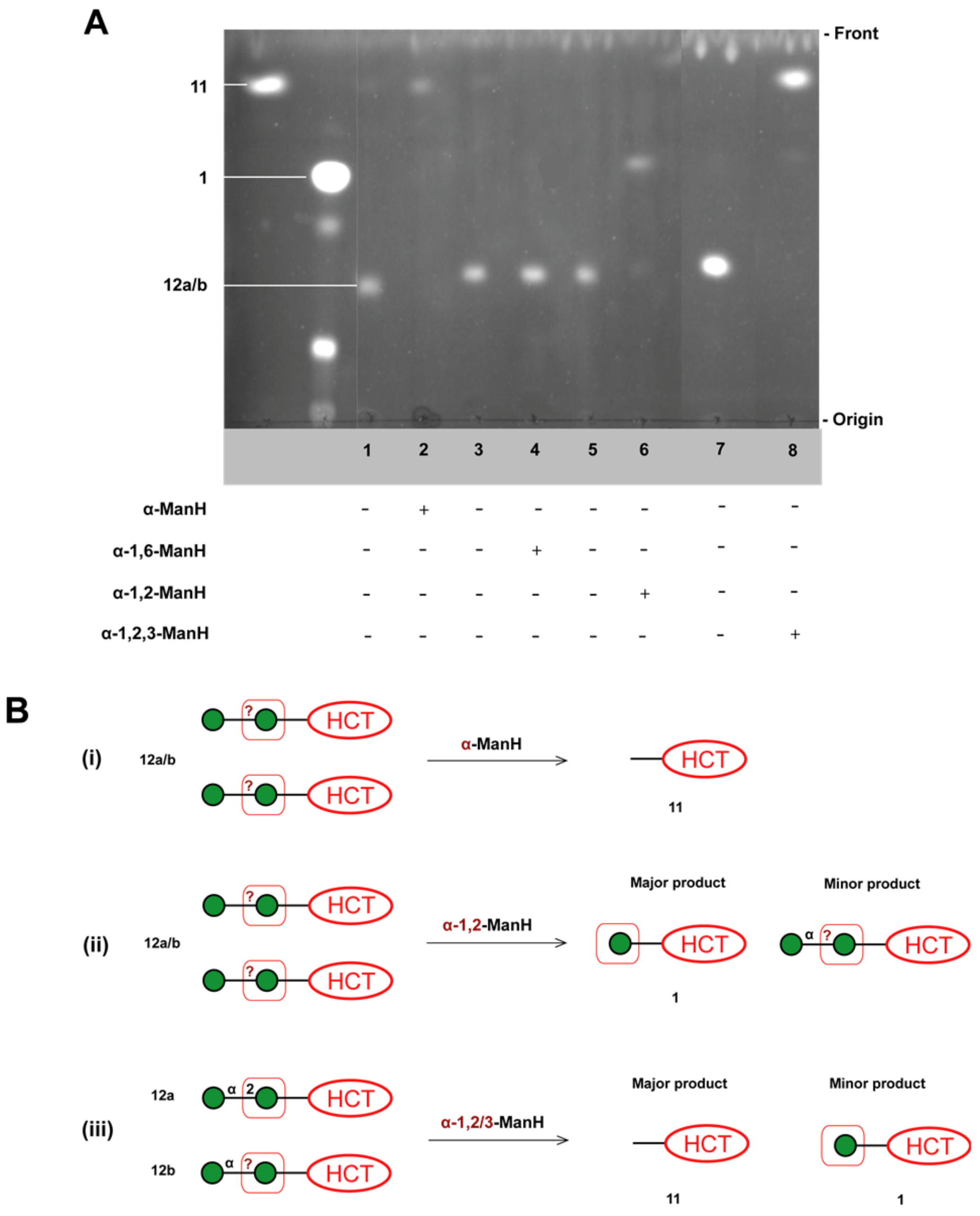

Fig. S11 TLC analyses to assess stereoselectivity and regioselectivity of disaccharide fluorescent products by *exo*-glycosidase digestions.
(**A**) TLC image of *exo*-glycosidase digestion of disaccharide 12a/b with jack bean α-mannosidase (α-ManH), *Xanthomonas manihotis* α-1,6-mannosidase (α-1,6-ManH), *Aspergillus saitoi* α-1,2-mannosidase (α-1,2-ManH) and *Xanthomonas manihotis* α-1,2/3-mannosidase (α-1,2/3-ManH). Enzymes used in the assays are shown below the TLC traces and acceptors and products are shown to the side of each TLC image. Control experiments, lane 1, 3, 5 and 7 (without enzyme), were conducted in parallel with hydrolysis reactions. TLC plates were eluted with $CHCl_3$:MeOH:H_2O (10:6:1) and visualised using mid-wave length range UV light. (B) Diagrammatic reaction schemes of *exo*-glycosidase digestion of 12a/b

Similarly, TLC analysis from jack bean α-mannosidase digestion of trisaccharide **13** revealed removal of three mannose residues releasing a fluorescent product with the same R_f value as fluorescent HCT (**11**) aglycone (Fig. S12A, lane 2 and Fig. S12B(i)). Incubation of trisaccharide **13** with *Xanthomonas manihotis* α-1,6-mannosidase also had no effect on fluorescent compound therefore, excluding formation of the α-1,6-

linkage (Fig. S12A, lane 4). The hydrolysis of **13** with *Aspergillus saitoi* α-1,2-mannosidase resulted in formation of only one fluorescent product with the same R_f value as fluorescent α-Man-1,6-α-Man-HCT (**2**), thus indicating introduction of α-1,2-mannopyranosidic linkage in this product (Fig. S12, line 6, and Fig. S12B (ii)).

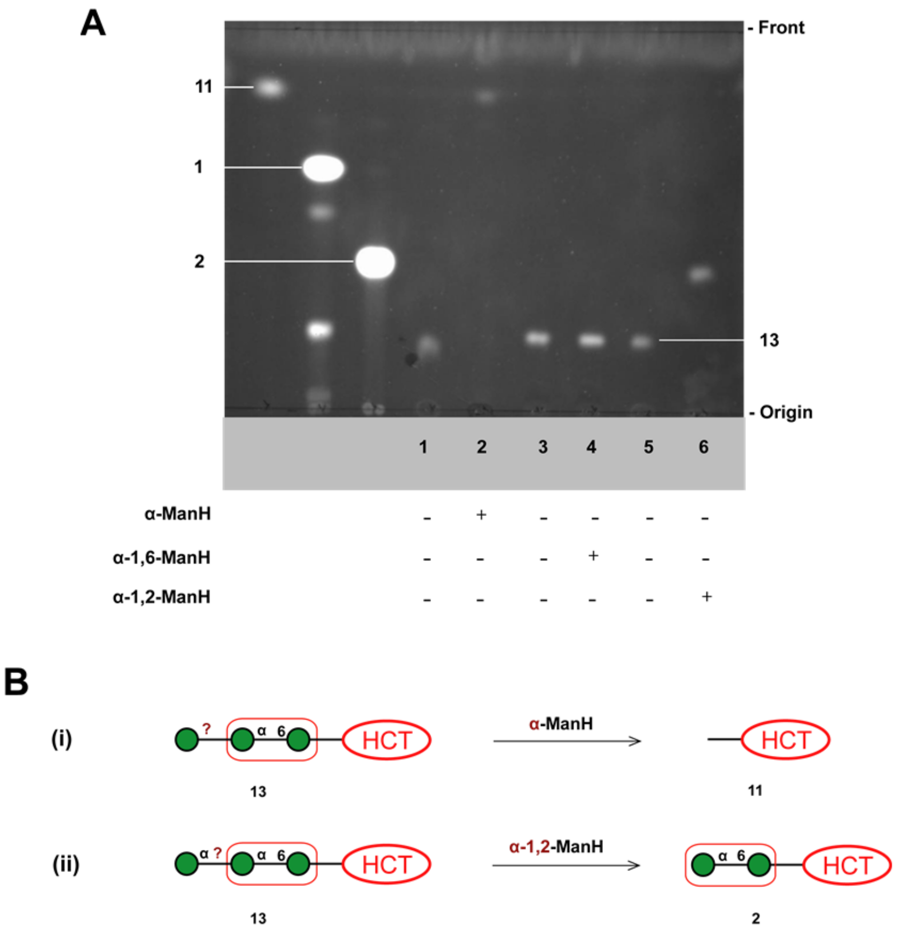

Fig. S12 TLC analyses to assess stereoselectivity and regioselectivity of trisaccharide fluorescent products by *exo*-glycosidaseF digestions.
(A) TLC image of *exo*-glycosidase digestion of trisaccharide **13** with jack bean α-mannosidase (α-ManH), *Xanthomonas manihotis* α-1,6-mannosidase (α-1,6-ManH) and *Aspergillus saitoi* α-1,2-mannosidase (α-1,2-ManH). Enzymes used in the assays are shown below the TLC traces and acceptors and products are shown to the side of each TLC image. Control experiments, lane 1, 3 and 5 (without enzyme), were conducted in parallel with hydrolysis reactions. TLC plates were eluted with $CHCl_3$:MeOH:H_2O (10:6:1) and visualised using mid-wave length range UV light. (B) Diagrammatic reaction schemes of *exo*-glycosidase digestion of **13** with (i) α-ManH, (ii) α-1,2-ManH.

5.1 Confirmation of newly formed linkages in fluorescent mannoside products by LC-MS

TLC analysis of *Aspergillus saitoi* α-1,2-mannosidase digestion revealed an incomplete hydrolysis of disaccharide **12a/b**, indicating presence of mixed fluorescent compounds with major α-1,2- **12a** and minor α-1,3-linkages **12b** (Fig. S11A, lane 6). The minor fluorescent disaccharide **12b** had a low fluorescent intensity on the TLC plate, thereby making final conclusions challenging. In order to confirm these TLC

observations, the reaction mixture from *Aspergillus saitoi* α-1,2-mannosidase digestion of disaccharide **12** was submitted to LC-MS analyses (Fig. S13B).

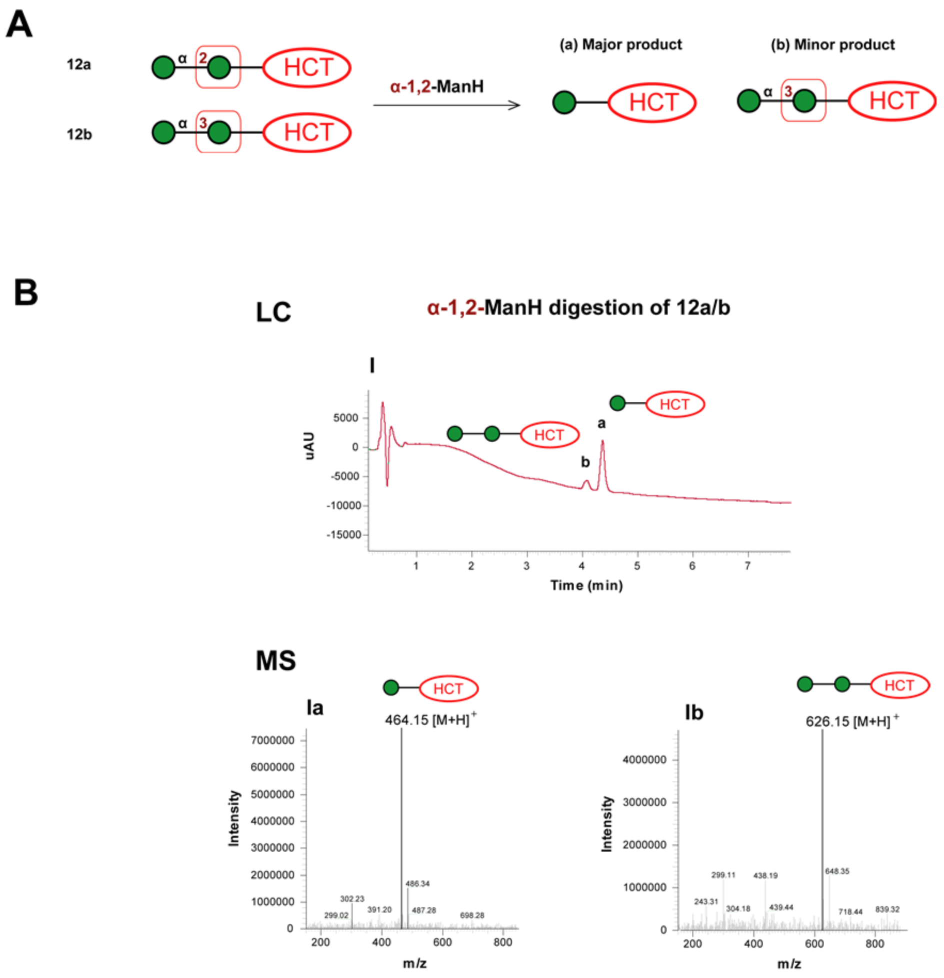

Fig. S13 Formation of two fluorescent disaccharide isomeric compounds confirmed by LC-MS analysis.
(A) Diagrammatic reaction schemes of *exo*-glycosidase digestion of 12a/b. (B I) UV chromatogram of reaction mixture from exo-glycosidase digestion of 12a/b with *Aspergillus saitoi* α-1,2-mannosidase (α-1,2-ManH). LC conditions: Kinetex C18 (50 mm/2.1 mm/2.6 µm); mobile phase: water (0.1% TFA)-CH_3CN (10 to 90 %, 18 min, flow rate: 0.3 mL/min); UV detector at 347 nm. (B Ia) MS spectrum of major monosaccharide product α-Man-HCT (1) obtained from hydrolysis of 12a/b; (B Ib) MS spectrum of minor disaccharide product α-Man-1,6-α-Man-HCT (**2**) obtained from hydrolysis of 12a/b.

The LC chromatogram from *Aspergillus saitoi* α-1,2-mannosidase digestion of disaccharide **12a/b** revealed presence of two peaks a minor peak (**b**) at retention time 4.0 min and a major (**a**) at 4.4 min (Fig.SI 13B, I). This result was in agreement with TLC observation verifying presence of two isomeric products in **12a/b** mixed compounds. The MS spectrum of peak (**a**) had *m/z* of 464.15, which corresponded to fluorescent α-Man-HCT (**1**) formed from **12a** as a result of the hydrolysis by *Aspergillus saitoi* α-1,2-mannosidase (Fig. S13B, Ia). The MS spectrum of peak (**b**) had *m/z* of 626.15, which corresponded to unhydrolysed **12b** (Fig. S13B, Ib).

The LC chromatogram of *Aspergillus saitoi* α-1,2-mannosidase digestion of trisaccharide **13** revealed presence of only one peak (**a**) at retention time 4.0 min (Fig. S14A and BI). The MS spectrum of peak (**a**) had *m/z* of 626.28, which corresponds to fluorescent α-Man-1,6-α-Man-HCT (**2**) (Fig. S14B, Ia) indicating the complete hydrolysis of **13** by α-1,2-mannosidase.

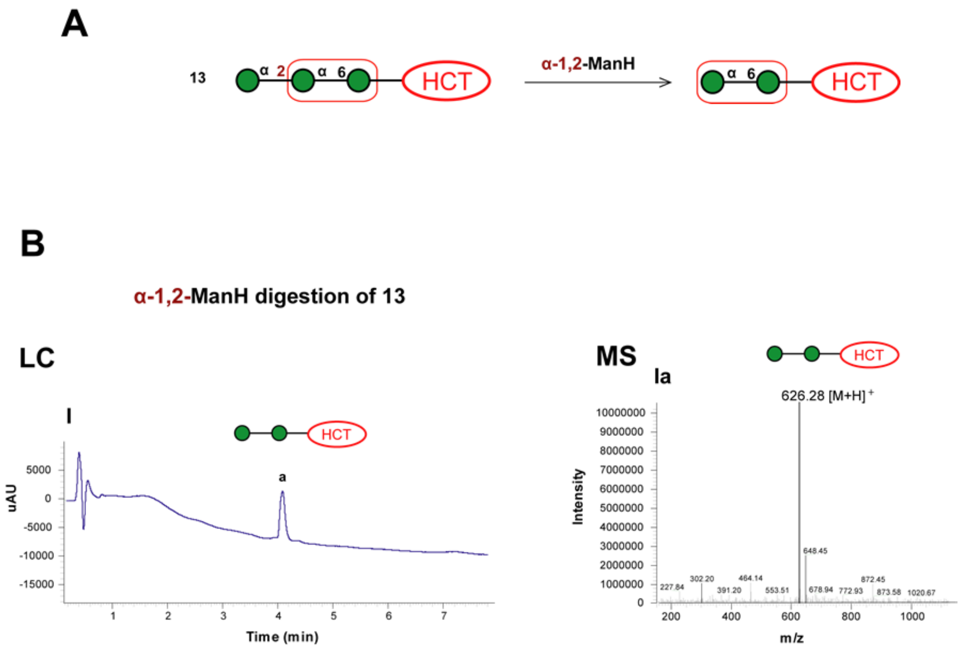

Fig. S14 Presence of one fluorescent trisaccharide 13 compound confirmed by LC-MS analysis.
(A) Diagrammatic reaction schemes of *exo*-glycosidase digestion of 13. (B I) UV chromatogram of reaction mixture from exo-glycosidase digestion of 13 with *Aspergillus saitoi* α-1,2-mannosidase (α-1,2-ManH). LC conditions: Kinetex C18 (50 mm/2.1 mm/2.6 µm), mobile phase: 0.1% aq. TFA-CH_3CN (10 to 90 %, 18 min, flow rate: 0.3 mL/min) UV detector at 347 nm. (B Ia) MS spectrum of disaccharide product α-Manα-1,6-Man-HCT (2) obtained from hydrolysis of 13.

6 Investigation of newly formed linkages in fluorescent products 15, 16, 18 and 19 through enzymatic and chemical degradations

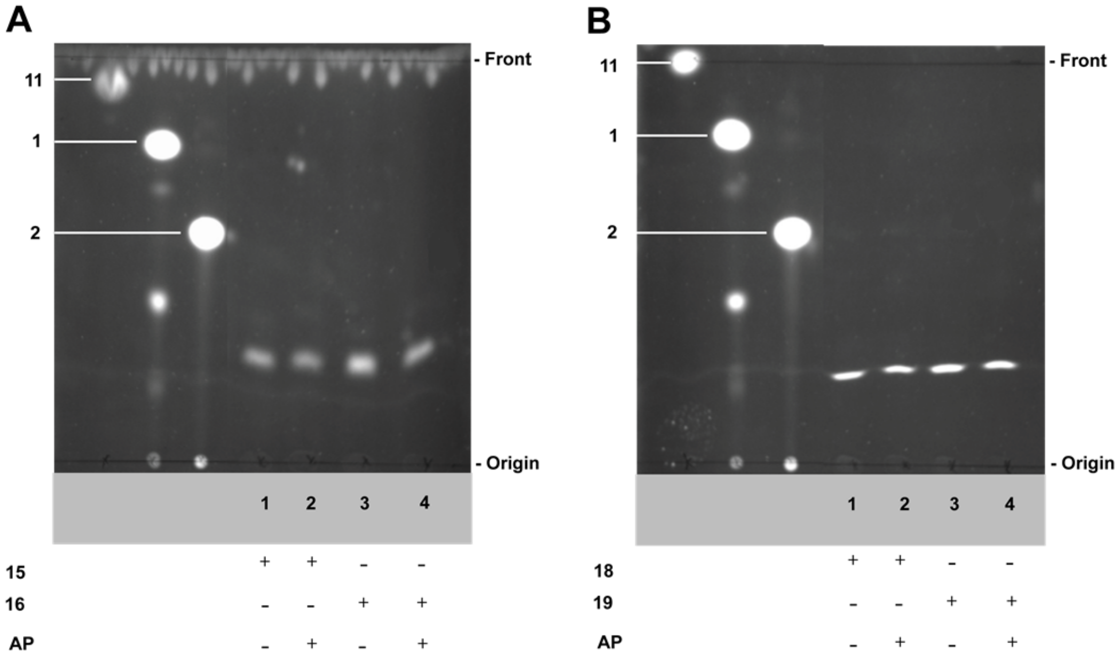

Fig. S15 TLC analyses to investigate presence of terminal phosphate in fluorescent products through application of enzymatic hydrolysis with alkaline phosphatase.

TLC images (A) Enzymatic hydrolysis of **15**/**16** (UDP-Glc) with alkaline phosphatase (AP); (B) Enzymatic hydrolysis of **18**/**19** (UDP-GlcNAc) with AP. Components and enzyme used in these assays are shown in the table below the TLC traces. Control experiments, lane 1 and 2 (without enzyme), were conducted in parallel with these assays. TLC plates were eluted with $CHCl_3$:MeOH:H_2O (10:8:2) and visualised using mid-wave length range UV light.

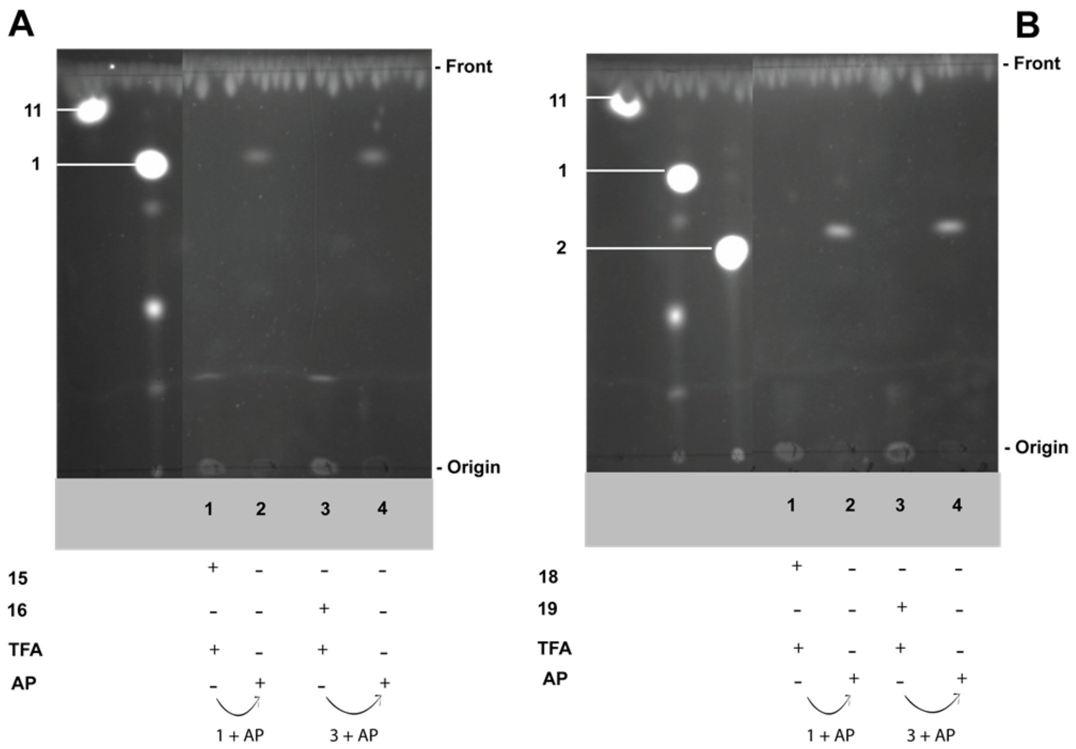

Fig. S16 TLC analyses to investigate the presence of phosphodiester bond in fluorescent products through application of chemical degradation and enzymatic hydrolysis.

Chemical degradation with TFA and enzymatic hydrolysis with AP (A) disaccharide 15/16 (B) trisaccharide 18/19. Components used in these assays are shown in the table below the TLC traces. Control experiments lane 1 and 2 (without enzyme) were conducted in parallel with these assays. The TLC plates eluted with $CHCl_3$:MeOH:H_2O (10:8:2) and visualised using mid-wave length range UV light.

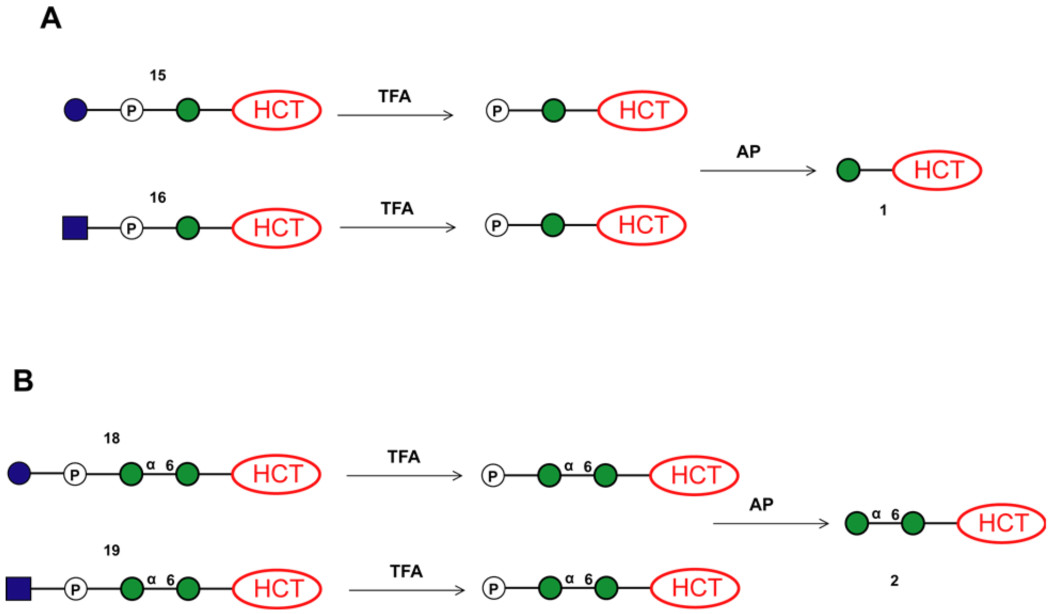

Fig. S 1

Fig. S17 Diagrammatic representation of sequential chemical and enzymatic degradation of fluorescent disaccharide and trisaccharide products containing phosphodiester bond.

TFA - trifluoroacetic acid and AP - alkaline phosphatase.

7 Characterisation of fluorescent product 19 by NMR spectroscopy

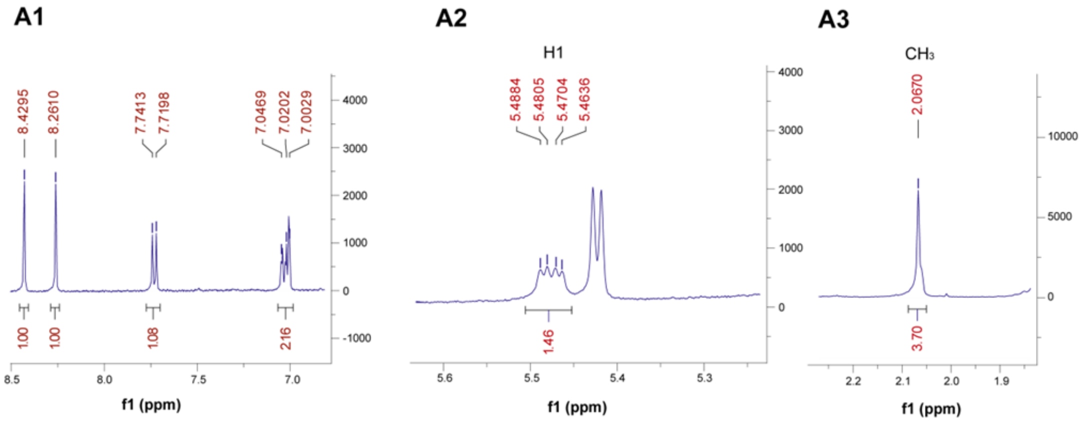

Fig. S18 Selected regions of ^{1}H NMR (800 MHz, D_2O, 25 °C) spectra of compound **19**.

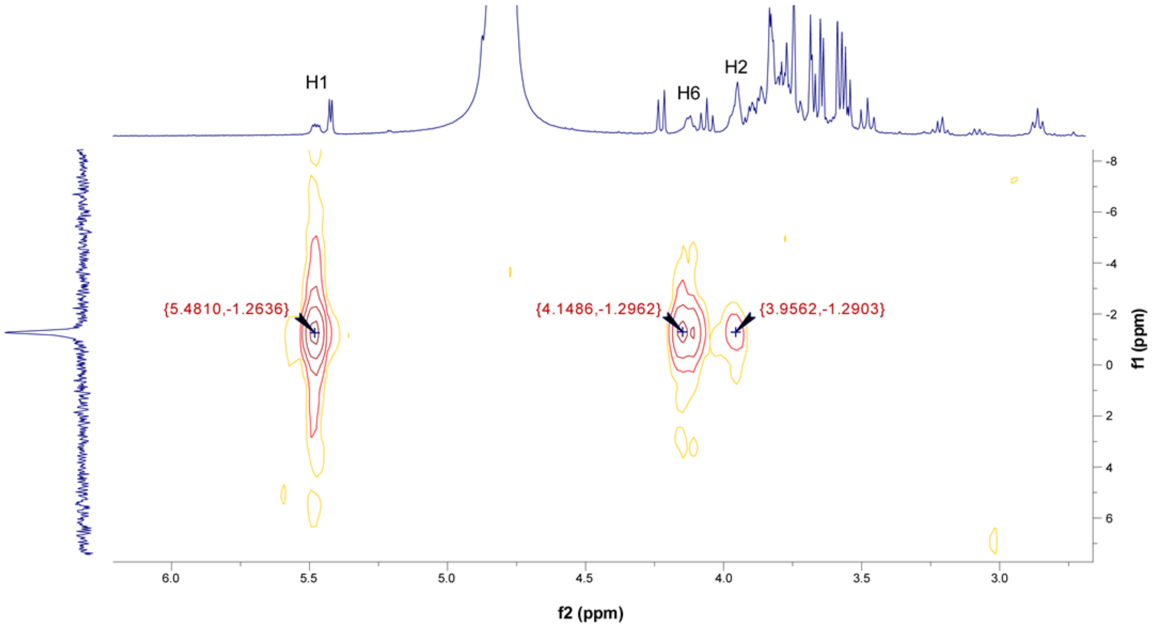

Fig. S19 ^{1}H–^{31}P HMBC spectrum (f1 ^{31}P and f2 ^{1}H) of compound **19**.

8 MS data and elemental composition for compound 19

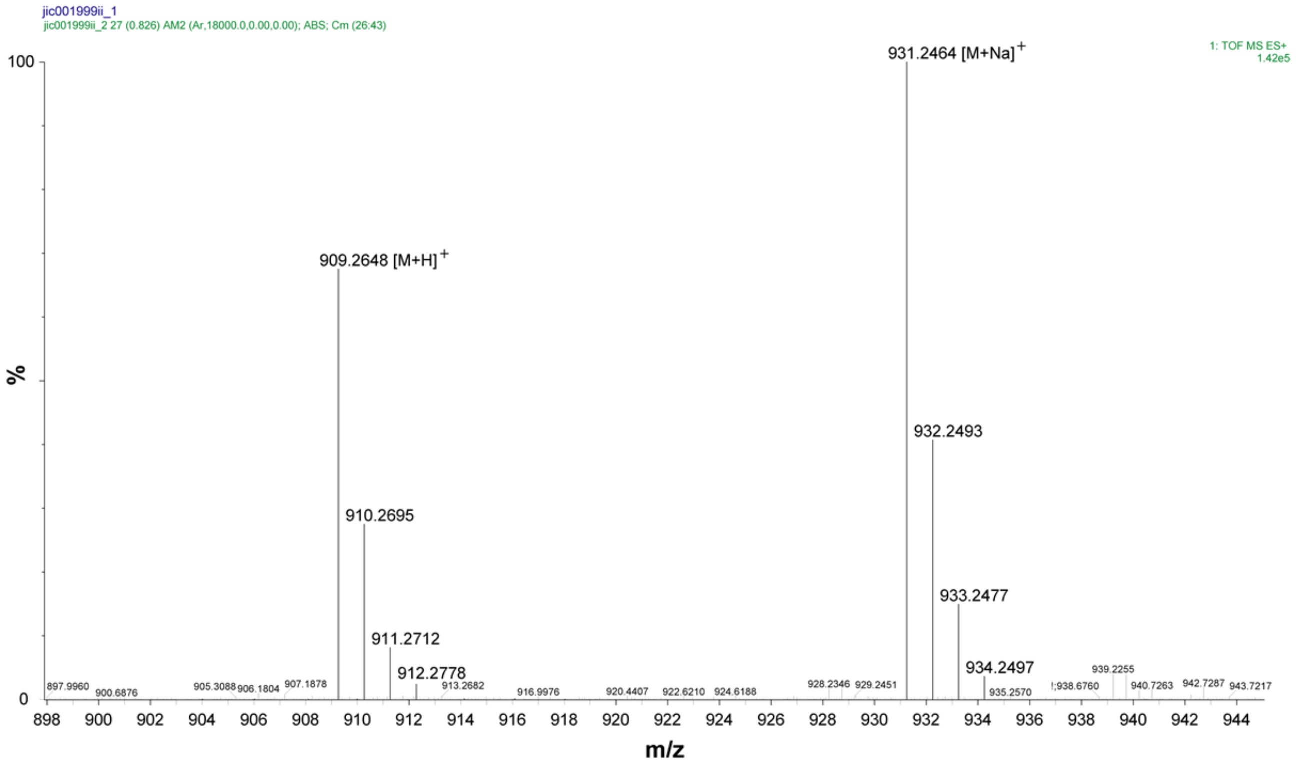

Fig. S19 High resolution MS spectrum of compound **19**

8.1 Elemental Composition Report

Single Mass Analysis

Tolerance = 5.0 PPM / DBE: min = -1.5, max = 50.0

Element prediction: On (Carbon range ± 3) (Set 0 < Cl < 9, 0< Br < 9 and 0 < S < 7 for enhanced filtering)

Number of isotope peaks used for i-FIT = 3

Monoisotopic Mass, Even Electron Ions

737 formula(e) evaluated with 3 results within limits (up to 50 best isotopic matches for each mass)

Elements Used:

C: 0-40 H: 0-55 N: 0-5 O: 0-25 P: 0-2

Minimum: -1.5

Maximum: 20.0 5.0 50.0

Table 1 Calculated elemental composition for compound **19**

Calc. Mass	mDa	PPM	DBE	Formula	i-FIT	Fit Conf (%)	C	H	N	O	P
909.2654	-0.6	-0.7	13.5	$C_{35}H_{50}N_4O_{22}P$	169.3	96.79	35	50	4	22	1

9 Experimental

9.1 3-Azido-7-hydroxy-2H-crhomen-2-one

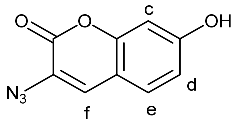

2,4-Dihydroxybenzaldehyde (20.0 g, 145 mmol), *N*-acetylglycine (16.9 g, 144 mmol) and anhydrous sodium acetate (38.0 g, 463 mmol) were suspended in acetic anhydride (500 mL) and refluxed at 140 °C under stirring for 12 h. The solvent was evaporated and resulting mixture was redissolved in water, extracted with EtOAc and organic solvent dried under reduced pressure to yield brown slurry. The resulting mixture was dispersed in a mixture of concentrated HCl and ethanol (400 mL), stirred under reflux for 2 h, then iced cold water (350 mL) was added to dilute the solution. The resulting solution was then cooled to 0 °C on ice bath, following addition of $NaNO_2$ (20 g, 290 mmol). The mixture was stirred for 30 min and NaN_3 (28 g, 434 mmol) was added in portions. After stirring for 12 h, the resulting precipitate was filtered off, washed with water and dried under reduced pressure to give azidocoumarin as a brown solid (3.7 g, 12%). ^{1}H NMR (400 MHz, DMSO) δ 7.57 (1H, s, H-f), 7.47 (1H, d, $J_{d,e}$ = 8.5 Hz, H-e), 6.81 (1H, dd, $J_{c,d}$ = 2.3 Hz, $J_{d,e}$ = 8.5 Hz, H-d), 6.76 (1H, d, $J_{c,d}$ = 2.3 H-c). ^{1}H NMR data were in accordance with the literature.[11]

9.2 General methods and materials for the biotransformations

All reagents used were of analytical grade and all solvents were HPLC grade. *Mycobacterium smegmatis* membranes were prepared by S.S. Gurcha and G.S. Besra; *Trypanosoma bruce* membranes were prepared by M.D. Urbaniak and M. Ferguson. Jack bean α-mannosidase and green coffee beans α-galactosidase were obtained from Sigma Aldrich, β-galactosidase from Calbiochem, *Xanthomonas manihotis* α-1,6-mannosidase from New England Biolabs and *Aspergillus saitoi* α-1,2-mannosidase from Prozyme. High resolution ESI MS data were obtained using the Waters Synapt G2 and Water Synapt G2-S MS detector coupled to T-wave ion mobility. TLC separations were performed at room temperature on aluminium-backed silica gel 60 F_{254} or glass plated silica gel 60 TLC plates (Merck). Samples from enzymatic transformations were applied onto a TLC plate in 2 µL aliquots and dried with a hairdryer between applications. The TLC plates were eluted with stated solvent mixture, air dried and directly visualised with the gel imager. In fluorescence-based assays concentrations for buffer, acceptors and donors are stated as a total concentration used in enzymatic reactions. Stated concentrations in each fluorescence-based assays are final concentration in solution.

9.2.1 Hydrolytic enzymes and procedures used for characterisation of biotransformation products.

9.2.1.1 Jack bean α-mannosidase

Purified fluorescent mannosylated products trimer and tetramer **12** and **13** (30 μM) were redissolved in 0.1 M sodium acetate buffer (pH 5.0) and incubated at 37 °C for 24 h with and without 0.75 U of jack bean α-mannosidase (30 μL final volume). After incubation, reactions were terminated by boiling for 5 min.

9.2.1.2 ***Green coffee bean α-galactosidase***

Fluorescent products (**trimer a,b**, Fig S9A) (30 μM) were redissolved in 50 mM phosphate buffer (pH 7.3) containing 10 mM of $MgCl_2$ and the reaction mixture was incubated at 37 °C for 24 h with and without 0.7 U of green coffee bean α-galactosidases (10 μL final volume). After incubation, reactions were terminated by addition of 10 μL of MeOH.

9.2.1.3 Escherichia coli β-galactosidase

Fluorescent products (**trimer a.b**, Fig S9A) (30 μM) were redissolved in 50 mM phosphate buffer (pH 6.5) and the reaction mixture was incubated at 21 °C for 24 h with and without 0.9 U of *E.coli* β-galactosidase (10 μL final volume). After incubation, reactions were terminated by addition of 10 μL of MeOH.

9.2.1.4 Aspergillus saitoi α-1,2-mannosidase

Purified fluorescent mannosylated products **12a,b** and **13a,b** (30 μM) were redissolved in reaction buffer (pH 5.0) and incubated at 37°C for 24 h with and without 80 mU of *Aspergillus saitoi* α-1,2-mannosidase from Prozyme (10 μL final volume). After incubation, reactions were terminated by boiling for 5 min.

9.2.1.5 Xanthomonas manihotis α-1,6-mannosidase

Purified fluorescent mannosylated products **12a,b** and **13a,b** (30 μM) were redissolved in GlycoBuffer 1 (5 mM $CaCl_2$, 50 mM sodium acetate, pH 5.5), supplemented with 100 μg/mL of BSA and incubated at 37 °C for 24 h with and without 80 mU of *Xanthomonas manihotis* α-1,6-mannosidase (30 μL final volume). After incubation, reactions were terminated by boiling for 5 min.

9.2.1.6 Xanthomonas manihotis α-1,2/3-mannosidase

Purified fluorescent mannosylated products **12a,b** and **13a,b** (30 μM) were redissolved in GlycoBuffer 1 (5 mM $CaCl_2$, 50 mM sodium acetate, pH 5.5), supplemented with 100 μg/mL of BSA and incubated at 37 °C for 24 h with and without 64 mU of *Xanthomonas manihotis* α-1,2/3-mannosidase (30 μL final volume). After incubation, reactions were terminated by boiling for 5 min.

9.2.1.7 Alkaline phosphatase digestion

To purified fluorescent product **15**, **18** and **16**, **19** (80 μM, 10 μL) were digested with 1 U of alkaline phosphatase in reaction buffer (50 mM, pH 8.0 Tris/HCl). The enzymatic reaction was mixed and incubated at 25 °C for 20 h in 20 μL of final volume. After incubation, reactions were terminated by boiling for 5 min.

9.2.1.8 Trifluoroacetic acid and alkaline phosphatase degradation

Purified fluorescent product **15**, **18** and **16**, **19** (60 μM, 10 μL) was treated with 40 mM of trifluoroacetic for 15 min at 100 °C. After drying, the sample was dissolved in 10 μL of water and digested with 1 U of alkaline phosphatase in reaction buffer (50 mM, pH 8.0 Tris/HCl). The enzymatic reaction was incubated at 25 °C for 20 h in 20 μL of final volume. After incubation reactions were terminated by boiling for 5 min.

9.3 Fluorescence-based assays to probe mannosyltransferase activities in Mycobacterium smegmatis

Buffers and incubation conditions from previously established cell-free radiolabelled assay were used to probe α-1,6-mannosyltransferase activities in fluorescence-based assay.[4]

In the fluorescence-based assay 250 μL acceptor (**2**) (1 mM) was incubated with 250 μL of GDP-Man (2 mM) in 250 μL of reaction buffer (50 mM, pH 7.9 MOPS/KOH, 10 mM $MgCl_2$, 5 mM DTT). The reaction was initiated by the addition of 250 μL of mycobacterial membranes (4000 μg of protein) in a total volume of 200 μL. Aliquots of 20 μL at 2 h, 4 h, 6 h, and 29 h were taken and each aliqoutes was stopped by addition of 20 μL of $CHCl_3$/MeOH (1:1, vv). After incubation for 29 h at 37 °C the main reaction was stopped with $CHCl_3$/MeOH (1:1, v/v) (80 μL). The denatured mycobacterial membranes were removed by several centrifugations (16000 g for 5 min) and washed with $CHCl_3$/MeOH:H_2O (10:6:1) (3 x 100 μL). The washings were combined and solvents dried under gentle stream of air. The residue was re-dissolved in deionised water and passed through a 0.45 μm PPTE filter, the filtrate was collected and the sample freeze dried.

9.4 Fluorescence-based assays to probe galactosyltransferase activities in Trypanosoma brucei

Buffers and incubation conditions from previously established cell-free radiolabelled assay were used to probe galactosyltransferase activities in fluorescence-based assay.[5] The reaction buffer composition was as follows: 100 mM, pH 7.4 HEPES/KOH, 50 mM KCl, 10 mM $MgCl_2$, 10 mM $MnCl_2$, 2 mM ATP, 2 mM dithiothreitol, 2 mg/mL leupeptin, 2.5 μg/mL tunicamycin, 0.2 mM *N*-tosyl-L-lysine chloromethyl ketone, 0.05% Triton X-100. *T. brucei* microsomal membranes were centrifuged (12000 rpm for 2 min) and resuspended in reaction buffer. Subsequently, 25 μL of UDP-Gal donor (1 mM) and 25 μL of acceptor **2** (1 mM) were freeze-dried and resuspended in 50 μL of reaction buffer. The reactions were initiated by the addition of 50 μL of *T. brucei* microsomal membranes in a total volume of 100 μL and incubated at 30 °C.

Aliquots of 20 μL were taken periodically and a mixture of $CHCl_3$/MeOH (20 μL, 1:1, v/v) was added to each aliquot in order to stop the reaction. After taking the last aliquot (24 h or 36 h) the main reaction was stopped with $CHCl_3$/MeOH (1:1, v/v) (40 μL). The resulting denatured *T. brucei* microsomal membranes were removed by several centrifugations (16000 g for 5 min) and washed with $CHCl_3$/MeOH:H_2O (10:6:1) (3 x 50 μL). The washings were combined and solvents dried under gentle stream of air. The residue was re-dissolved in deionised water and passed through a 0.45 μm PPTE filter, the filtrate was collected, and the sample was freeze-dried for storage.

9.4.1 Variations of enzyme concentration

Two fluorescence assays were performed as described above but with different amount of *T. brucei* microsomal membranes added. In the first assay of microsomal membranes were prepared from $1x10^7$ cells and in the second assay from $5x10^8$ cells. In both cases aliquots were taken after 2 h, 6 h, and 24 h, treated as described above and analysed by TLC (see Fig. S6 and Fig. S7A).

9.4.2 Increasing donor concentration

The fluorescence assay was performed as described above with addition of *T. brucei* microsomal membranes obtained from $1x10^8$ cells. Aliquots (10 μL) were taken after incubation for 4 h, 8 h, 12 h, 24 h and 36 h and analysed by TLC (see Fig S7B). At the same time points (4 h, 8 h, 12 h, 24 h) the mixture was supplemented with 5 μL of UDP-Gal donor (4 mM).

9.5 Euglena gracilis growth medium composition

The composition of the medium used for growing *Euglena gracilis* culture in the dark was based on EG:JM medium (https://www.ccap.ac.uk/media/documents/EG_JM.pdf) and further modified as indicated in Table 2. To make EG medium all constituents stated in table 2 were dissolved in 1 L deionised water. JM medium was prepared by mixing 1 mL stock solutions listed in Table 2 and diluted to male 1 L solution. EG:JM medium consisted of 1:1 mixture of EG and JM media.

Table 2. EG:JM medium composition

EG medium		JM medium		
Medium	*Per 1 L*	*Stock*		*Per 200 mL*
$CaCl_2$ (stock solution 1g/dm^{-3})	10.0 mL	**1**	$Ca(NO_3)_2 \cdot 4H_2O$	4.0 g
NaOAc $3 \cdot H_2O$	1.0 g	**2**	KH_2PO_4	2.48 g
Casein hydrolysate	3 g	**3**	$MgSO_4 \cdot 7H_2O$	10.0 g
Yeast extract	2.0 g	**4**	$NaHCO_3$	3.18 g
Glucose	15 g	**5**	EDTAFeNa<br>$EDTANa_2$	0.45 g<br>0.45 g
		**6**	H_3BO_3<br>$MnCl_2.4H_2O$<br>$(NH_4)_6Mo_7O_{24} \cdot 4H_2O$	0.496 g<br>0.278 g<br>0.20 g
		**7**	Cyanocobalamin<br>Thiamine·HCl<br>Biotin	0.008 g<br>0.008 g<br>0.008 g
		**8**	$NaNO_3$	16.0 g
		**9**	$Na_2HPO_4 \cdot 12H_2O$	7.2 g

.

10 NMR data

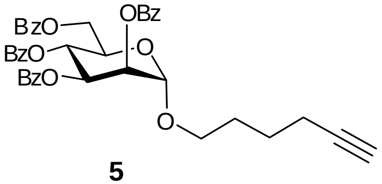

OBz
BzO
BzO
BzO
O
O

5

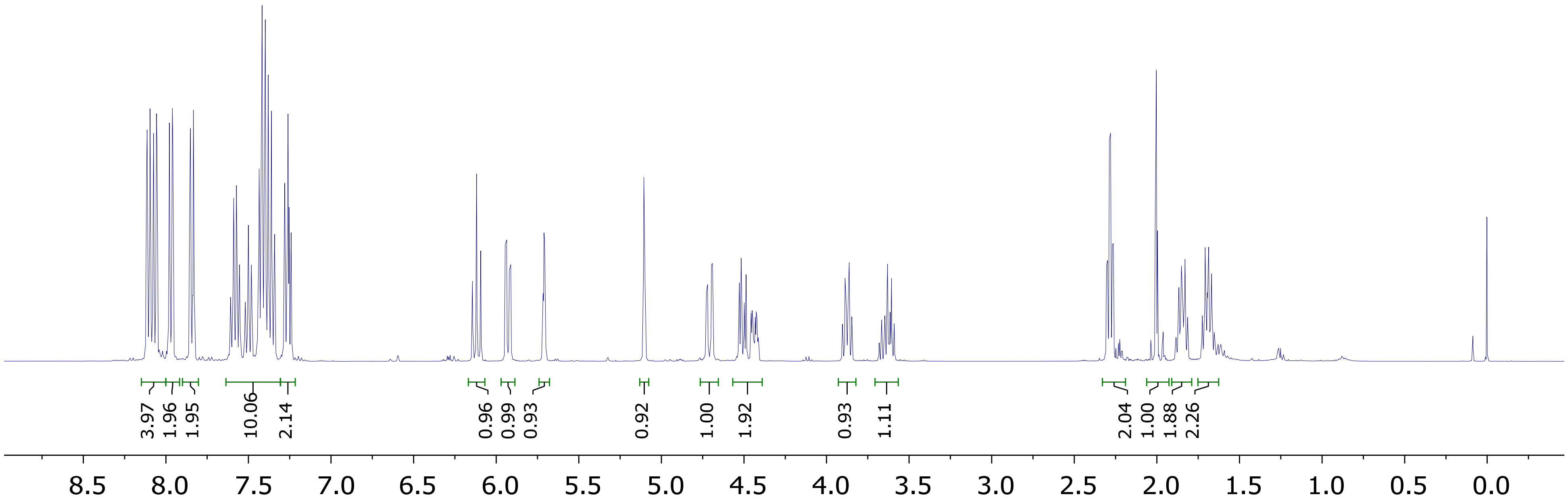

3.97
1.96
1.95
10.06
2.14
0.96
0.99
0.93
0.92
1.00
1.92
0.93
1.11
2.04
1.00
1.88
2.26
8.5
8.0
7.5
7.0
6.5
6.0
5.5
5.0
4.5
4.0
3.5
3.0
2.5
2.0
1.5
1.0
0.5
0.0

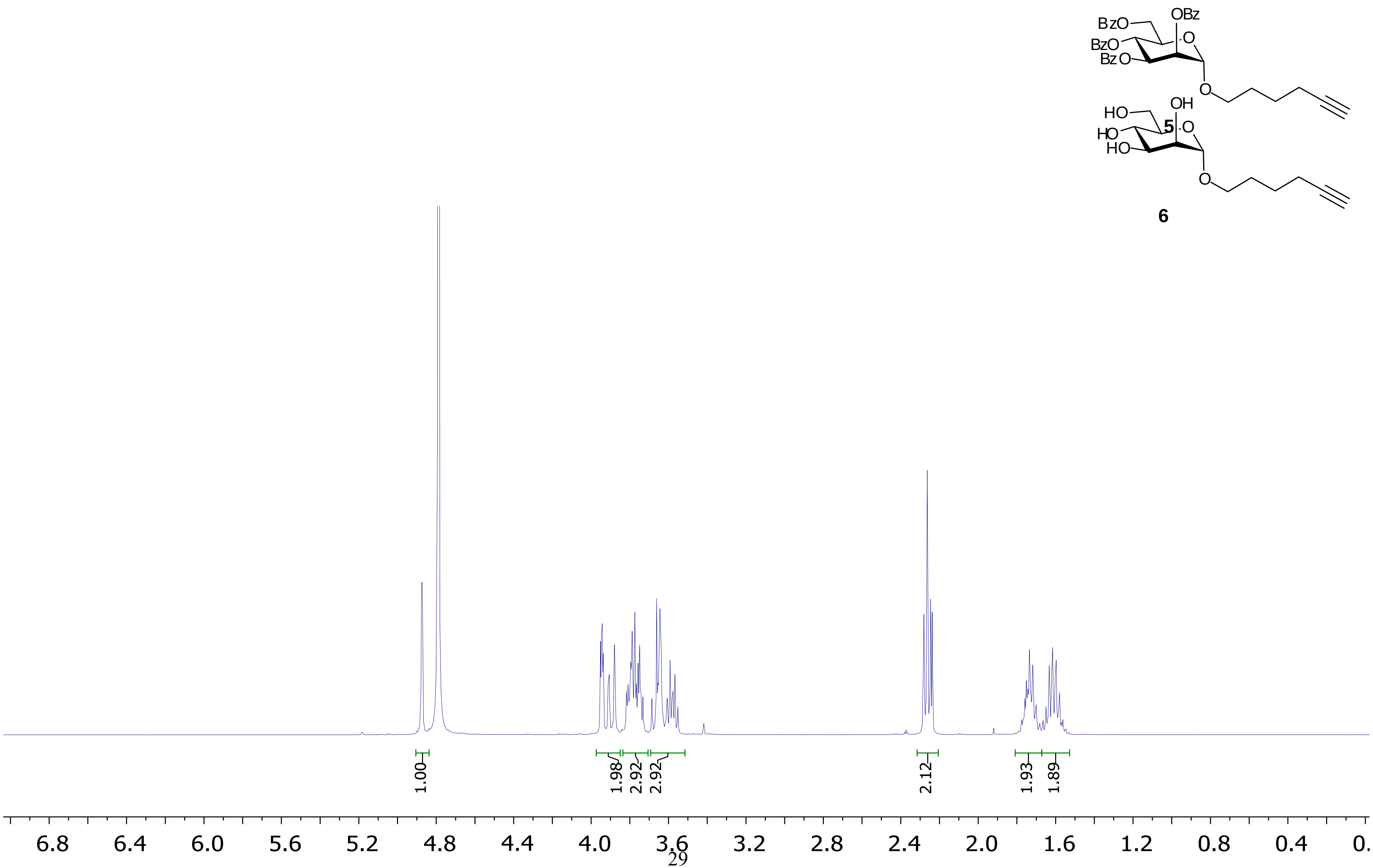
BzO
OBz
O
BzO
BzO
O
5
HO
OH
O
HO
HO
O
6
1.00
1.98
2.92
2.92
2.12
1.93
1.89
6.8
6.4
6.0
5.6
5.2
4.8
4.4
4.0
3.6
3.2
2.8
2.4
2.0
1.6
1.2
0.8
0.4

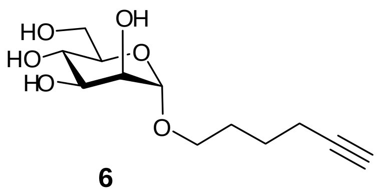

6

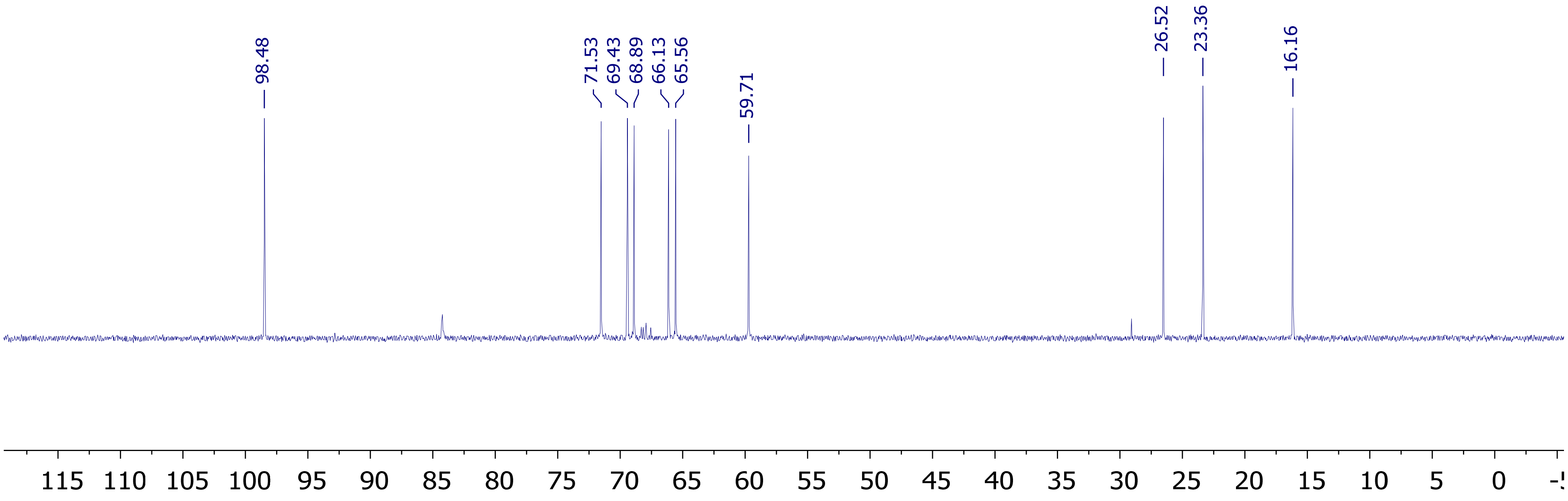
98.48
71.53
69.43
68.89
66.13
65.56
59.71
26.52
23.36
16.16
115 110 105 100 95 90 85 80 75 70 65 60 55 50 45 40 35 30 25 20 15 10 5 0

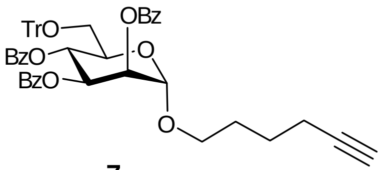
TrO
OBz
O
BzO
BzO
O

7

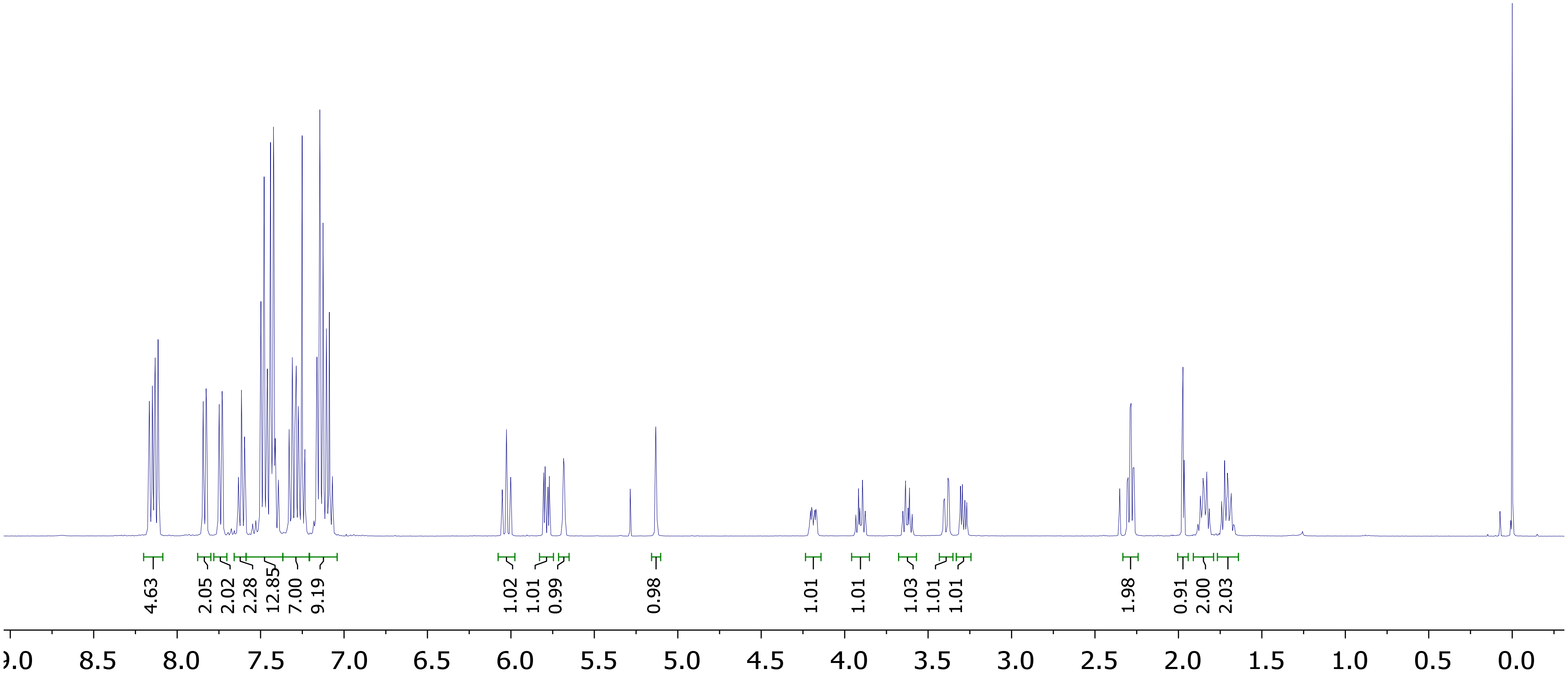
4.63
2.05
2.02
2.28
12.85
7.00
9.19
1.02
1.01
0.99
0.98
1.01
1.01
1.03
1.01
1.01
1.98
0.91
2.00
2.03
8.5
8.0
7.5
7.0
6.5
6.0
5.5
5.0
4.5
4.0
3.5
3.0
2.5
2.0
1.5
1.0
0.5
0.0

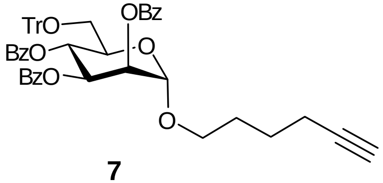
TrO
OBz
BzO
BzO
O
O

7

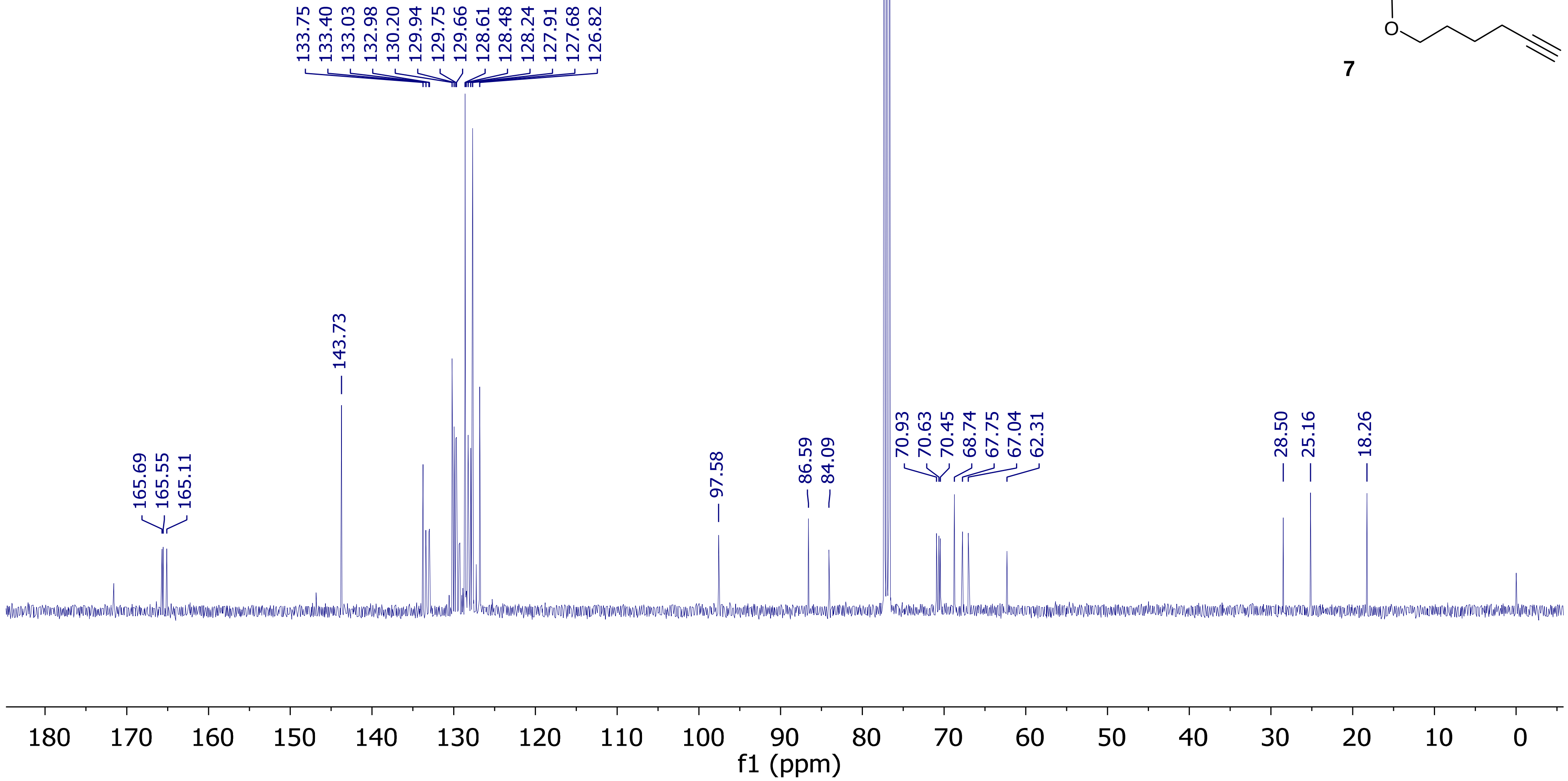
133.75
133.40
133.03
132.98
130.20
129.94
129.75
129.66
128.61
128.48
128.24
127.91
127.68
126.82
143.73
165.69
165.55
165.11
97.58
86.59
84.09
70.93
70.63
70.45
68.74
67.75
67.04
62.31
28.50
25.16
18.26
180
170
160
150
140
130
120
110
100
90
80
70
60
50
40
30
20
10
0
f1 (ppm)

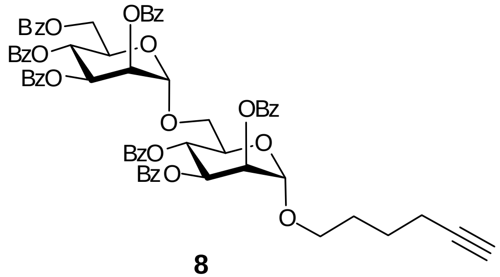

8

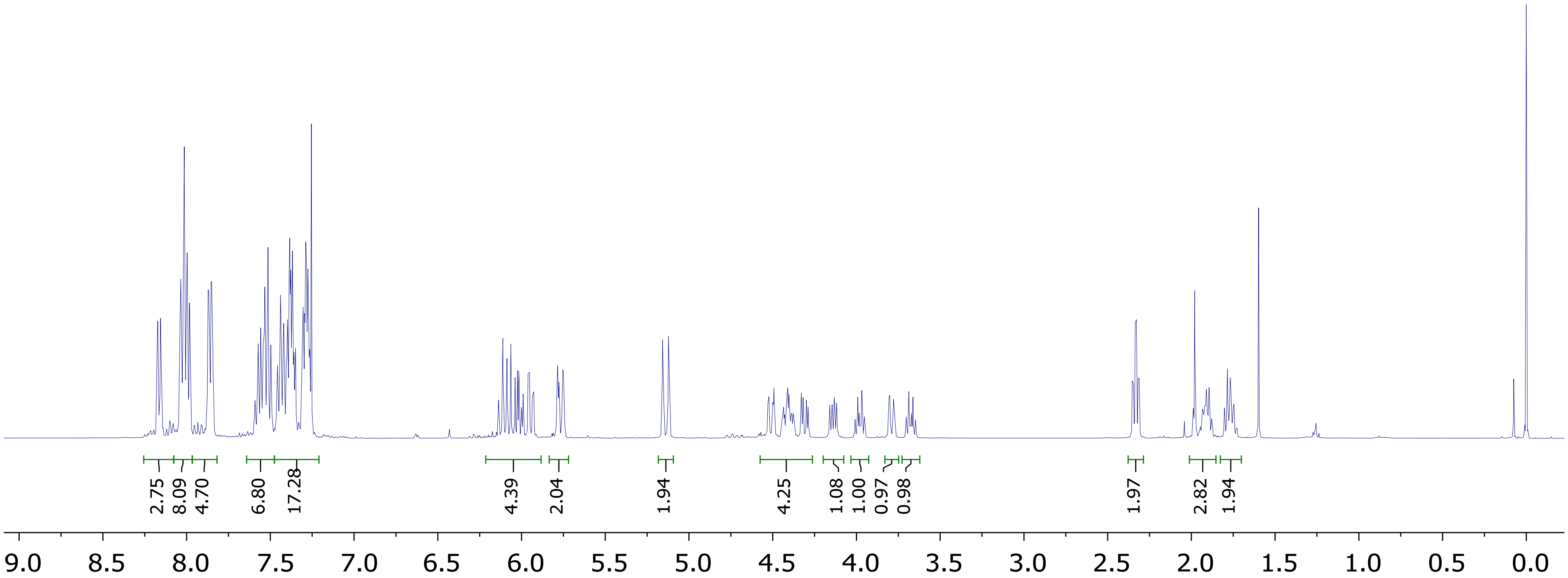
2.75
8.09
4.70
6.80
17.28
4.39
2.04
1.94
4.25
1.08
1.00
0.97
0.98
1.97
2.82
1.94
9.0 8.5 8.0 7.5 7.0 6.5 6.0 5.5 5.0 4.5 4.0 3.5 3.0 2.5 2.0 1.5 1.0 0.5 0.0

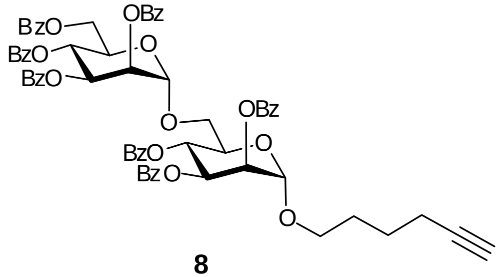

BzO
BzO
BzO
OBz
O
O
OBz
O
BzO
BzO
O

8

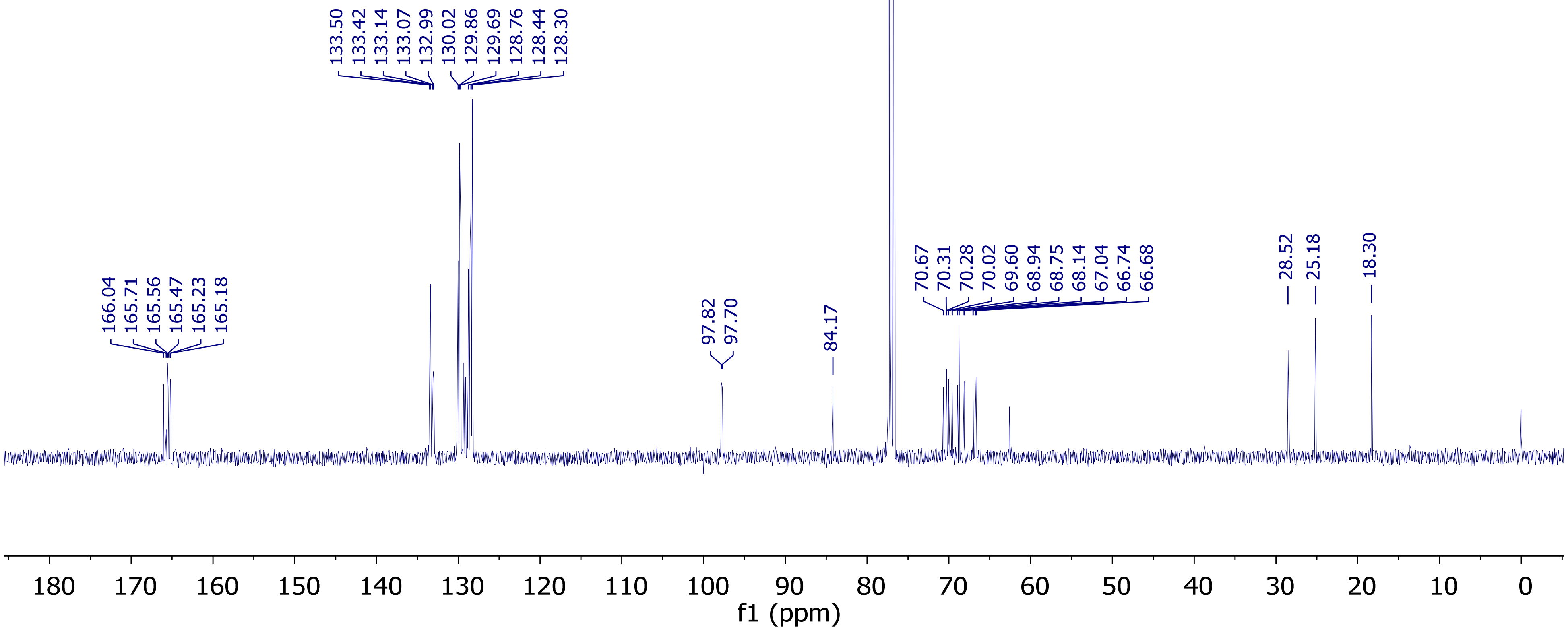

166.04
165.71
165.56
165.47
165.23
165.18
133.50
133.42
133.14
133.07
132.99
130.02
129.86
129.69
128.76
128.44
128.30
97.82
97.70
84.17
70.67
70.31
70.28
70.02
69.60
68.94
68.75
68.14
67.04
66.74
66.68
28.52
25.18
18.30
180
170
160
150
140
130
120
110
100
90
80
70
60
50
40
30
20
10
0
f1 (ppm)

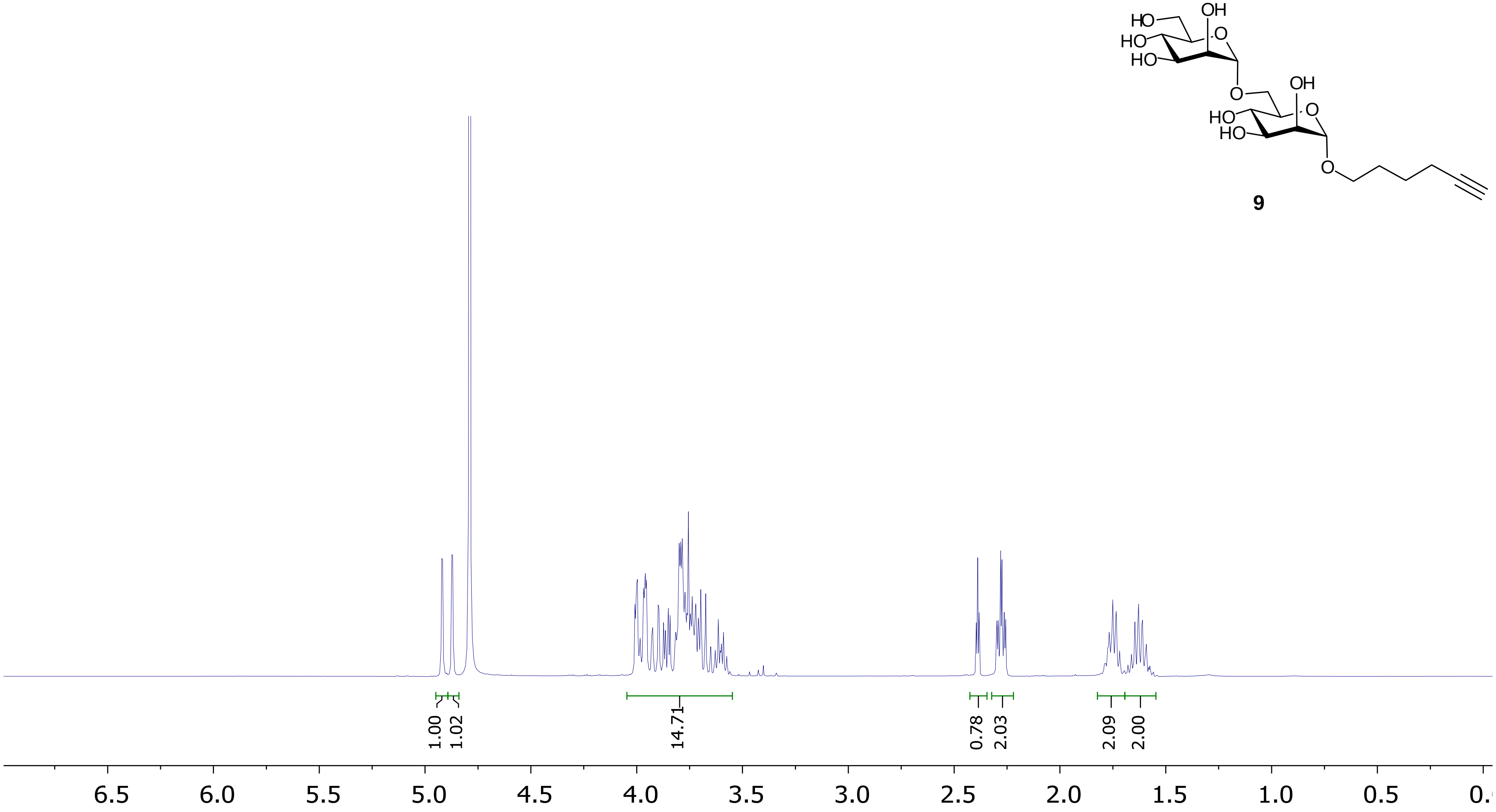
HO
HO
HO
OH
O
O
OH
HO
HO
O
O
9
1.00
1.02
14.71
0.78
2.03
2.09
2.00
6.5
6.0
5.5
5.0
4.5
4.0
3.5
3.0
2.5
2.0
1.5
1.0
0.5

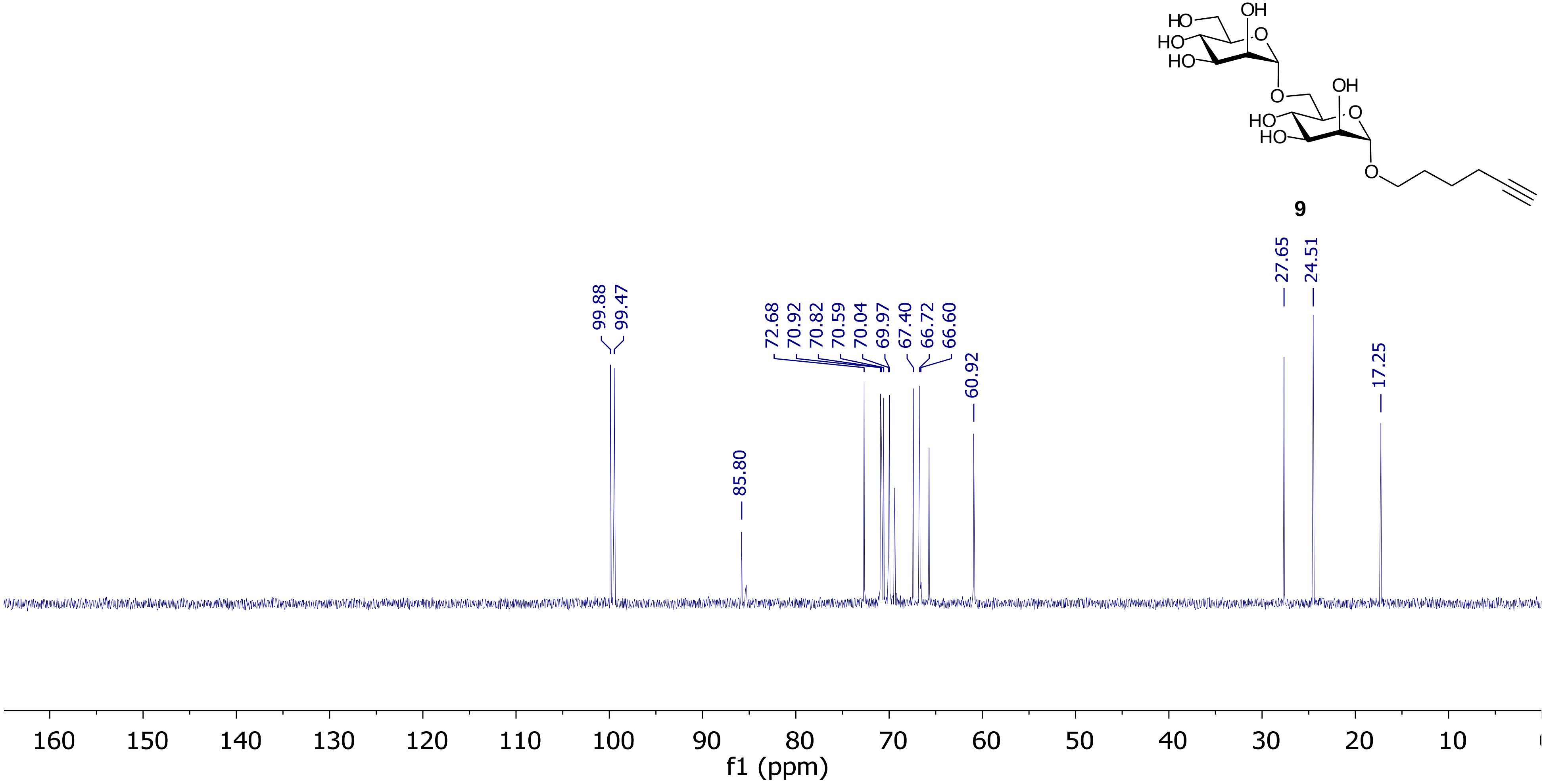

9
99.88
99.47
85.80
72.68
70.92
70.82
70.59
70.04
69.97
67.40
66.72
66.60
60.92
27.65
24.51
17.25
160
150
140
130
120
110
100
90
80
70
60
50
40
30
20
10
f1 (ppm)

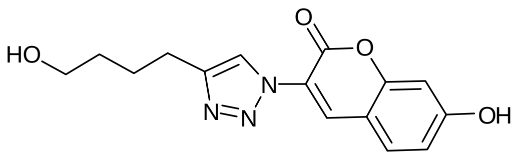
HO
O
O
N
N
N
OH

11

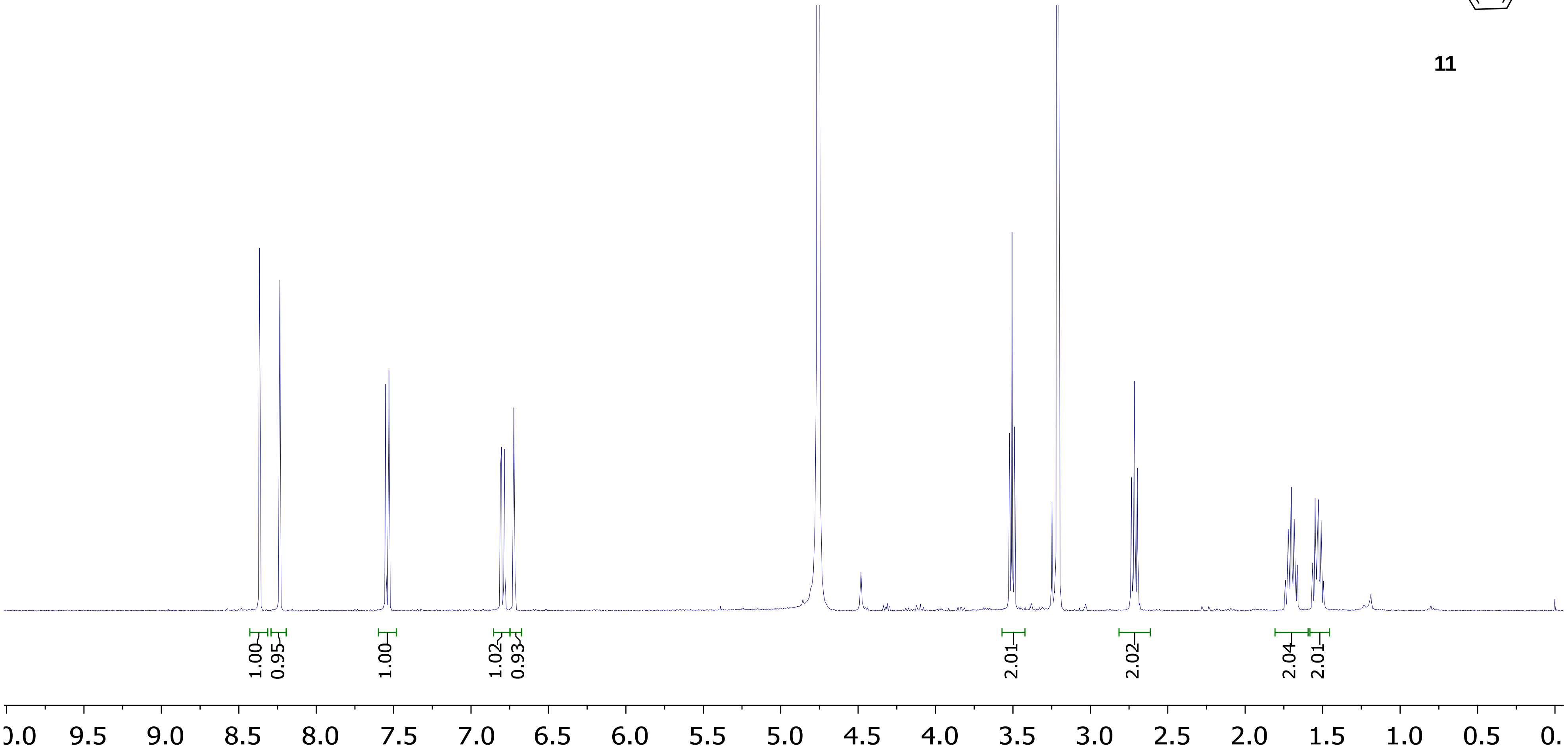
1.00
0.95
1.00
1.02
0.93
2.01
2.02
2.04
2.01
9.5
9.0
8.5
8.0
7.5
7.0
6.5
6.0
5.5
5.0
4.5
4.0
3.5
3.0
2.5
2.0
1.5
1.0
0.5

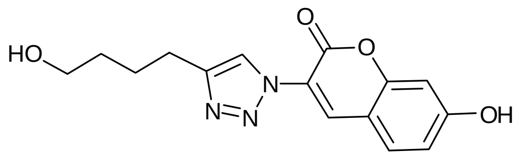
HO
O
O
N
N=N
OH

11

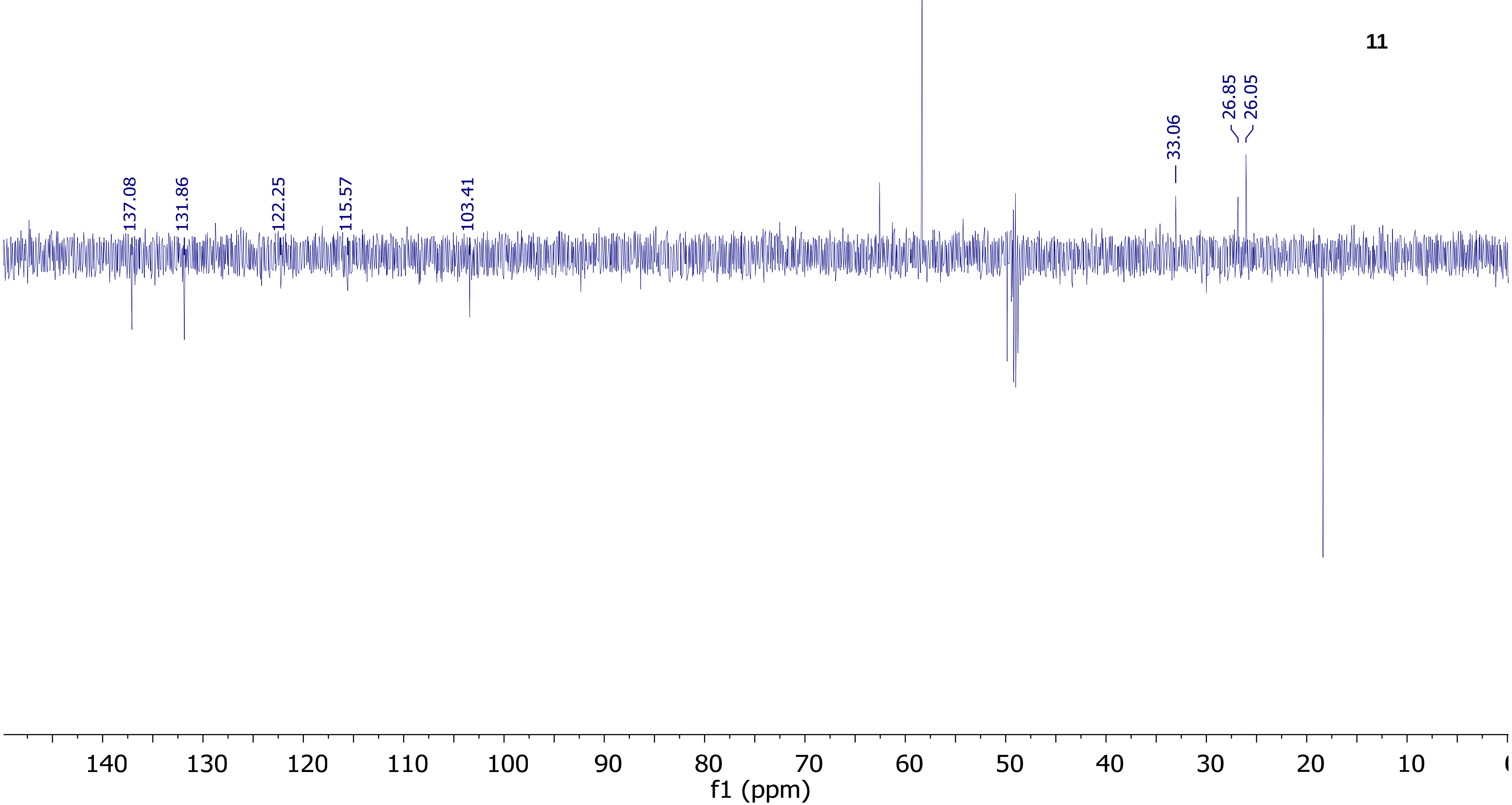
137.08
131.86
122.25
115.57
103.41
33.06
26.85
26.05
140
130
120
110
100
90
80
70
60
50
40
30
20
10
f1 (ppm)

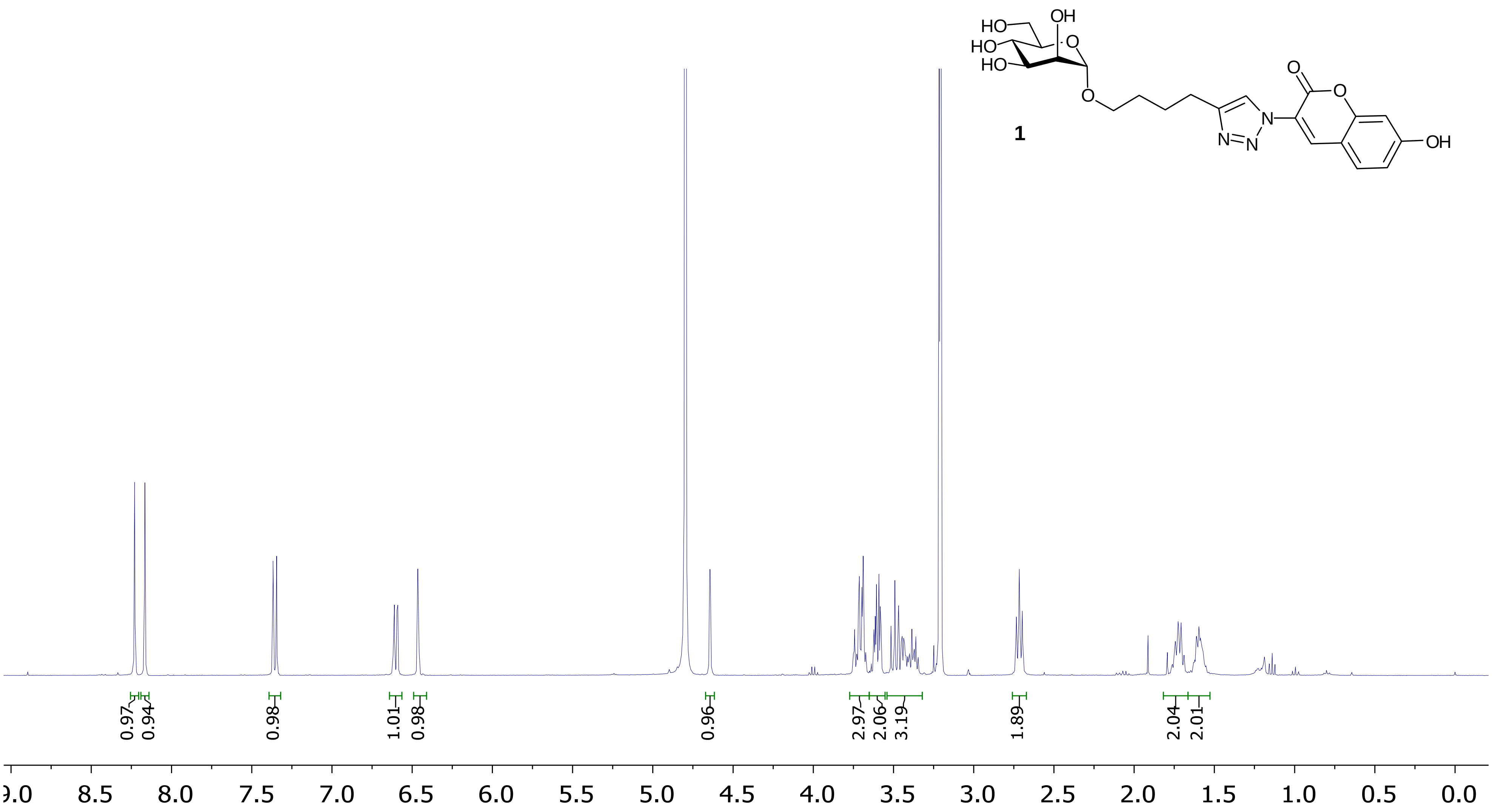
HO
HO
HO
OH
O
O
1
O
O
N
N
N
OH
0.97
0.94
0.98
1.01
0.98
0.96
2.97
2.06
3.19
1.89
2.04
2.01
8.5
8.0
7.5
7.0
6.5
6.0
5.5
5.0
4.5
4.0
3.5
3.0
2.5
2.0
1.5
1.0
0.5
0.0

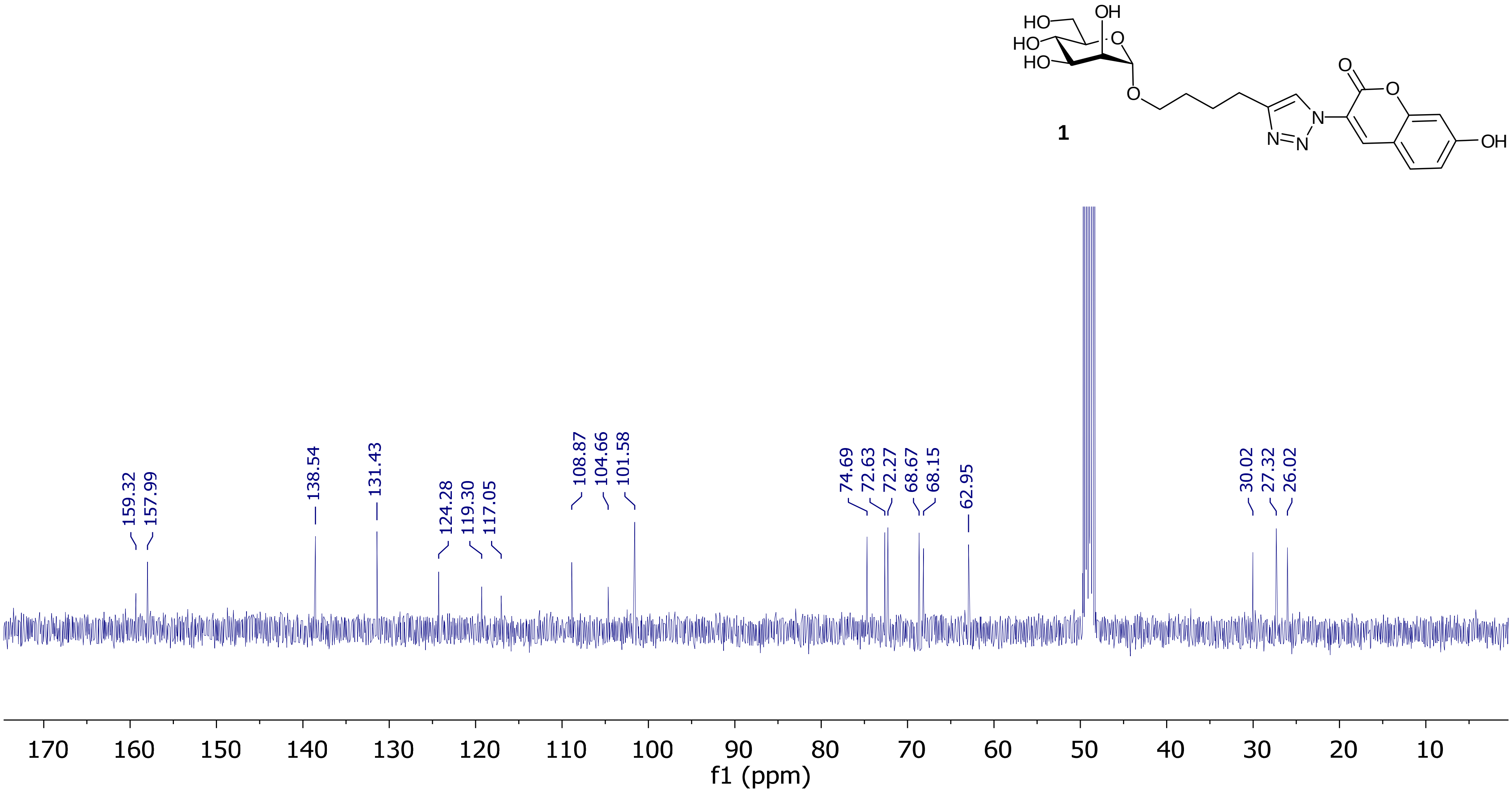
HO
HO
HO
OH
O
O
O
O
O
N
N=N
OH
1
159.32
157.99
138.54
131.43
124.28
119.30
117.05
108.87
104.66
101.58
74.69
72.63
72.27
68.67
68.15
62.95
30.02
27.32
26.02
170 160 150 140 130 120 110 100 90 80 70 60 50 40 30 20 10
f1 (ppm)

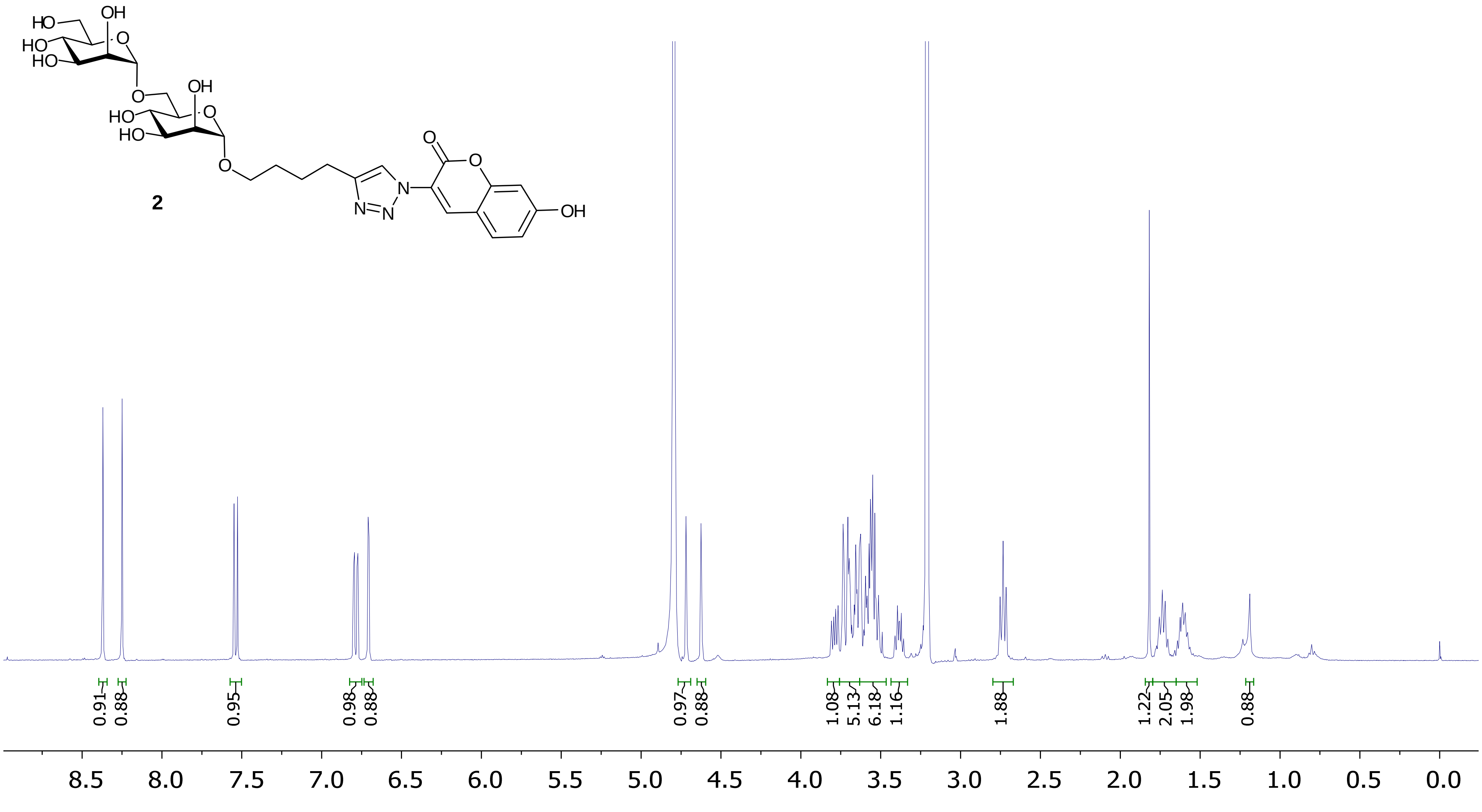
2
0.91
0.88
0.95
0.98
0.88
0.97
0.88
1.08
5.13
6.18
1.16
1.88
1.22
2.05
1.98
0.88
8.5 8.0 7.5 7.0 6.5 6.0 5.5 5.0 4.5 4.0 3.5 3.0 2.5 2.0 1.5 1.0 0.5 0.0

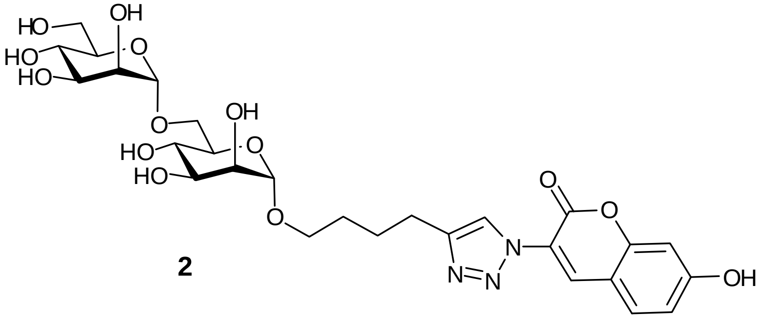
HO
OH
O
HO
HO
O
OH
O
HO
HO
O
O
O
N
N=N
OH

2

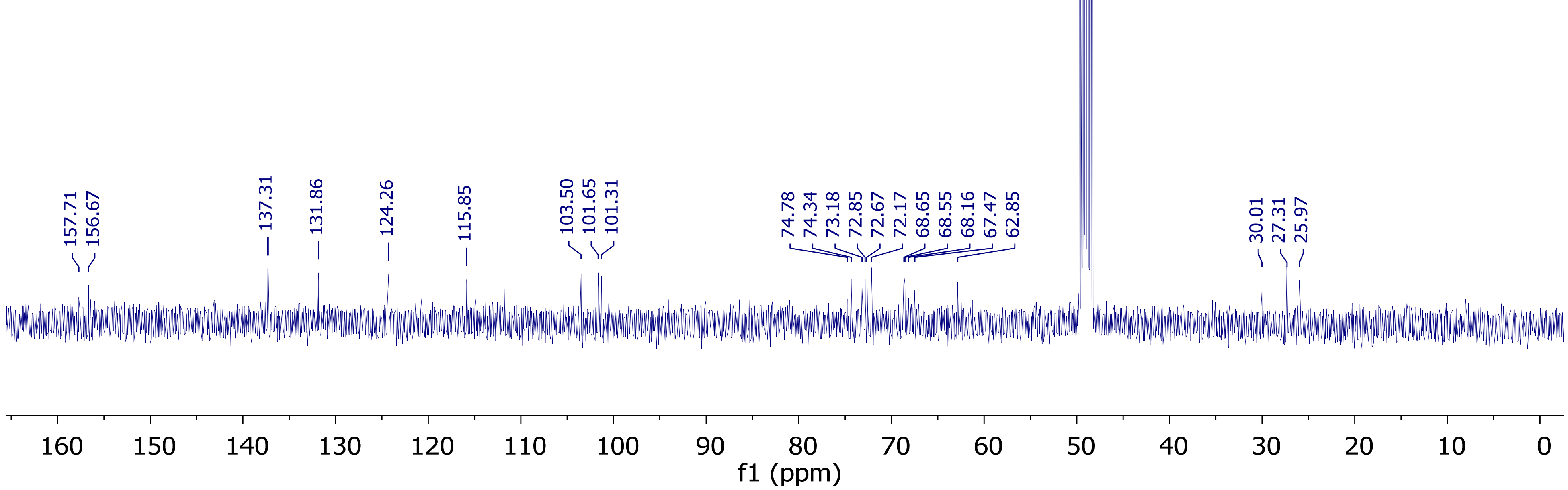
157.71
156.67
137.31
131.86
124.26
115.85
103.50
101.65
101.31
74.78
74.34
73.18
72.85
72.67
72.17
68.65
68.55
68.16
67.47
62.85
30.01
27.31
25.97
160 150 140 130 120 110 100 90 80 70 60 50 40 30 20 10 0
f1 (ppm)

11 References:

1. Bruckner, J., Estimation of monosaccharides by the orcinol-sulphuric acid reaction. *Biochem. J.* **1955**, *60*, 200-205.
2. Tam, P. H.; Besra, G. S.; Lowary, T. L., *ChemBioChem* **2008**, *9*, 267-278.
3. Subramaniam, V.; Gurcha, S. S.; Besra, G. S.; Lowary, T. L., *Bioorg. Med. Chem.* **2005**, *13*, 1083-1094.
4. Brown, J. R.; Field, R. A.; Barker, A.; Guy, M.; Grewal, R.; Khoo, K. H.; Brennan, P. J.; Besra, G. S.; Chatterjee, D., *Bioorgan. Med. Chem.* **2001**, *9*, 815-824.
5. Pingel, S.; Field, R. A.; Guther, M. L. S.; Duszenko, M.; Ferguson, M. A. J., *Biochem. J.* **1995**, *309*, 877-882.
6. Cross, G. A., *Parasitology* **1975**, *71*, 393-417.
7. Masterson, W. J.; Doering, T. L.; Hart, G. W.; Englund, P. T., *Cell* **1989**, *56*, 793-800.
8. Smith, T. K.; Crossman, A.; Paterson, M. J.; Borissow, C. N.; Brimacombe, J. S.; Ferguson, M. A. J., *J. Biol. Chem.* **2002**, *277*, 37147-37153.
9. Brown, J. R.; Guther, M. L. S.; Field, R. A.; Ferguson, M. A. J., *Glycobiology* **1997**, *7*, 549-558.
10. Corfield, A. P., *Methods Mol. Biol.* **1993**, *19*, 269-286.
11. K. Sivakumar, F. Xie, B.M. Cash, S. Long, H.N. Barnhill, Q. Wang, *Org. Lett.*, **2004**, *6*, 4603-4606.
