# Supplementary material for: Fluorescent mannosides serve as acceptor substrates for glycosyltransferase and sugar-1-phosphate transferase activities in Euglena gracilis membranes
Source: Carbohydr Res. 2017 Jan 13;438:26–38. doi: 10.1016/j.carres.2016.11.017 (PMC5240791; doi:10.1016/j.carres.2016.11.017)
Supplement: Supplementary file 2 [file mmc2.pdf]

Supplementary Information (Part 2)

Fluorescent mannosides serve as acceptor substrates for glycosyltransferase and sugar-1-phosphate transferase activities in *Euglena gracilis* membranes

Irina M. Ivanova, Sergey A. Nepogodiev, Gerhard Saalbach, Ellis C. O'Neill, Michael D. Urbaniak, Michael A. J. Ferguson, Sudagar S. Gurcha, Gurdyal S. Besra and Robert A. Field*

Predicted protein sequence of N-acetylglucosamine-1-phosphotransferase from *Euglena gracilis*

For more information see: http://jicbio.nbi.ac.uk/euglena/

```
>Euglena
IFFSVRRYATAAPLTRAMARDVWLAAAGKLLQRQCYTFLGSRVGLALLFI
  ASVVTLNSVLQVIDGWFSSGIVCPHRCDWDWRDNLQRRSFEAFLGHEPID
  VVYTWVNGSDPRLKATLEEWKHAGLPHDAAHAPNATPAAPNASRAANASA
  AAHDRADASRYQDNQELRYSLRSVEHFAGWVRHIYIVTNGQVPAWLDLNN
  PRVTVVPHAALFPNHSHLPTFSSPAIEAHLHRIPGLSRRFLYLNDDTFFG
  NYIRPEDFYTPIHGHRIYLSWAVPDCSPGCTGTWIGDGYCDKACNVSACN
  FDGGDCLGKAASSYGSNWDNYDYGSYNYHWNPYAQTSCHSGCMDNWIGDK
  FCDTACNVEACGFDATDCGVDEVRKRLHSFHLSPNLTNYSVDGRLNAFVI
  NLTLGLPAGMEVRAARHRGGGDAIRATALSKPHHLLVIVLKALDRRQPVA
  FALEGEAHGAHVELNFTVVVGRDRPPNTTNHTGNLTSNMTNDNVGPTSSP
  QLGAPLHALTPSPAPPPATSHQSAAALLDPSAAPLGHSPPAATPAATTLV
  VAKPPTSPDPAQTPAHAPSPPSTPAGSSKPHPAPPGLDSKDTASDPPAPS
  PTPQPSPPDVATARRLLQLRRRIARPAERSEPVLVPMLEPAPPYRLRLPS
  PPPTAAPAPQGVNDPFADSGRPGEAPRGIDPRDLQAAVVGAERLSRLDAL
  AQHSPRPAVRTTPAGPGRAGQRRLLDTFGDSLKFVNHLFTVKWGHHPRKV
  PAHMPHMIDRDVMAALWREWPAQWEATSAAHFRSAADMQFAFSHFYYIVH
  AKAPVDPIQFFQERLDINGNGYLEPQEFRRLAVLLFDKKVTKESLDQITV
  VDGDVAPRNATVAKARNTTAAATNATGAAAPNSTGAHRSLLYLDEDDWGY
  GAGNIAATSTEEKTETGHSYYGWDDDDLVYGKPKGGSSGGAGANRSGGYA
  WDSSGSRYSSAASHQEHGEPTMPKEYLDAVARVVGLATGGVGLLDATRFV
  ASGLSKLVIGDKQAATYKHQLVDLDEVSFFMIRDNASTVQHQMDHILAKQ
  PKFICINDNMNHSHPENSKVVAVIHDFFEIYFPWRSSFELPAGKTNPYQY
  LDDMAAARKARRKAAGKFSAAVVCLVGLAVFYSFVGLNAVLHWRPGHRRQ

  IHDRHDV
```

Multiple alignment of putative N-acetylglucosamine-1-phosphotransferase from *Euglena gracilis* to known protein sequences

Protein BLAST https://blast.ncbi.nlm.nih.gov/Blast.cgi?PAGE=Proteins

Multiple alignment Clustal Omega https://www.ebi.ac.uk/Tools/msa/clustalo/

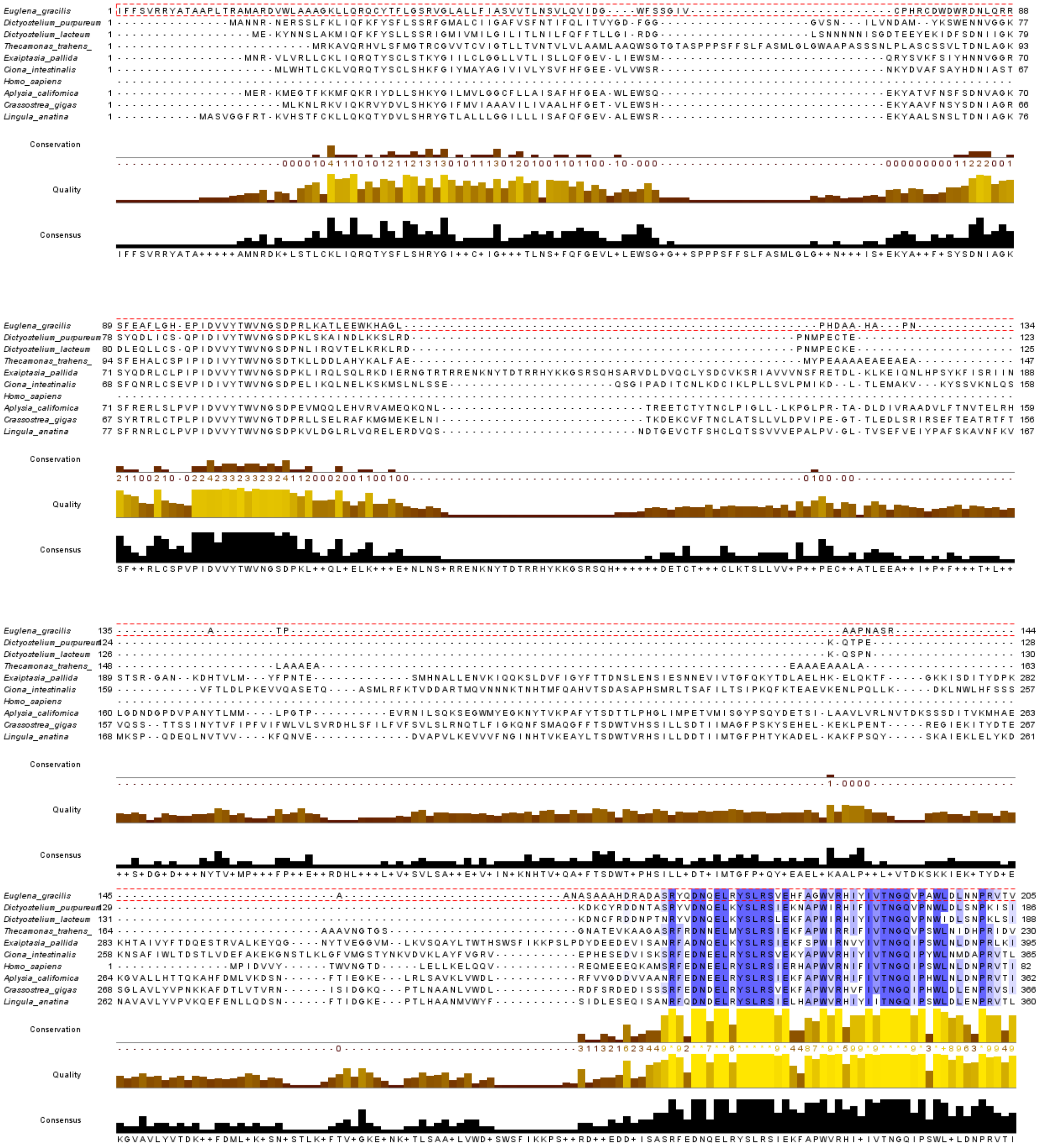

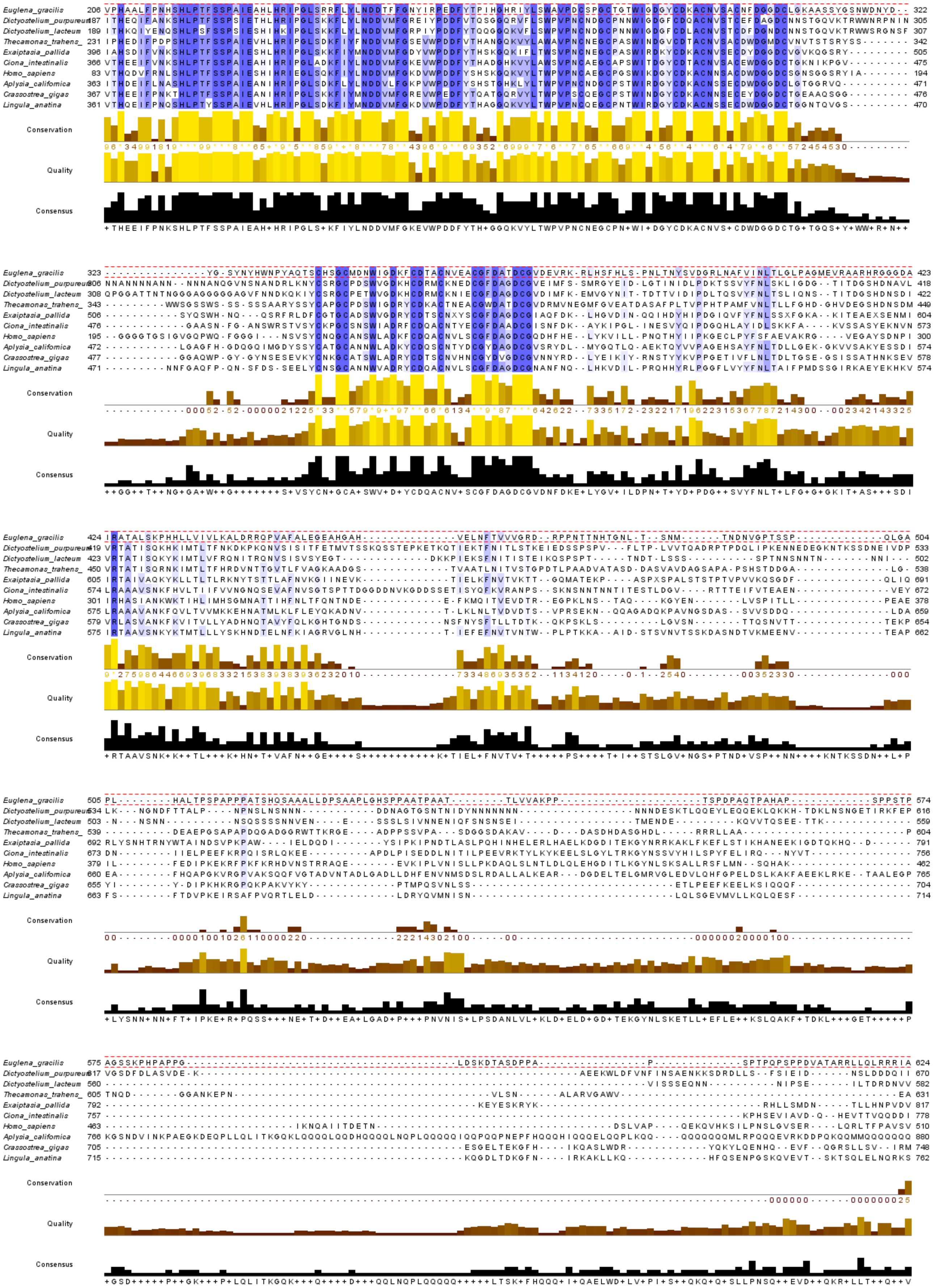
Euglena_gracilis
Dictyostelium_purpureum
Dictyostelium_lacteum
Thecamonas_trahens_
Exaiptasia_pallida
Ciona_intestinalis
Homo_sapiens
Aplysia_californica
Crassostrea_gigas
Lingula_anatina
Conservation
Quality
Consensus

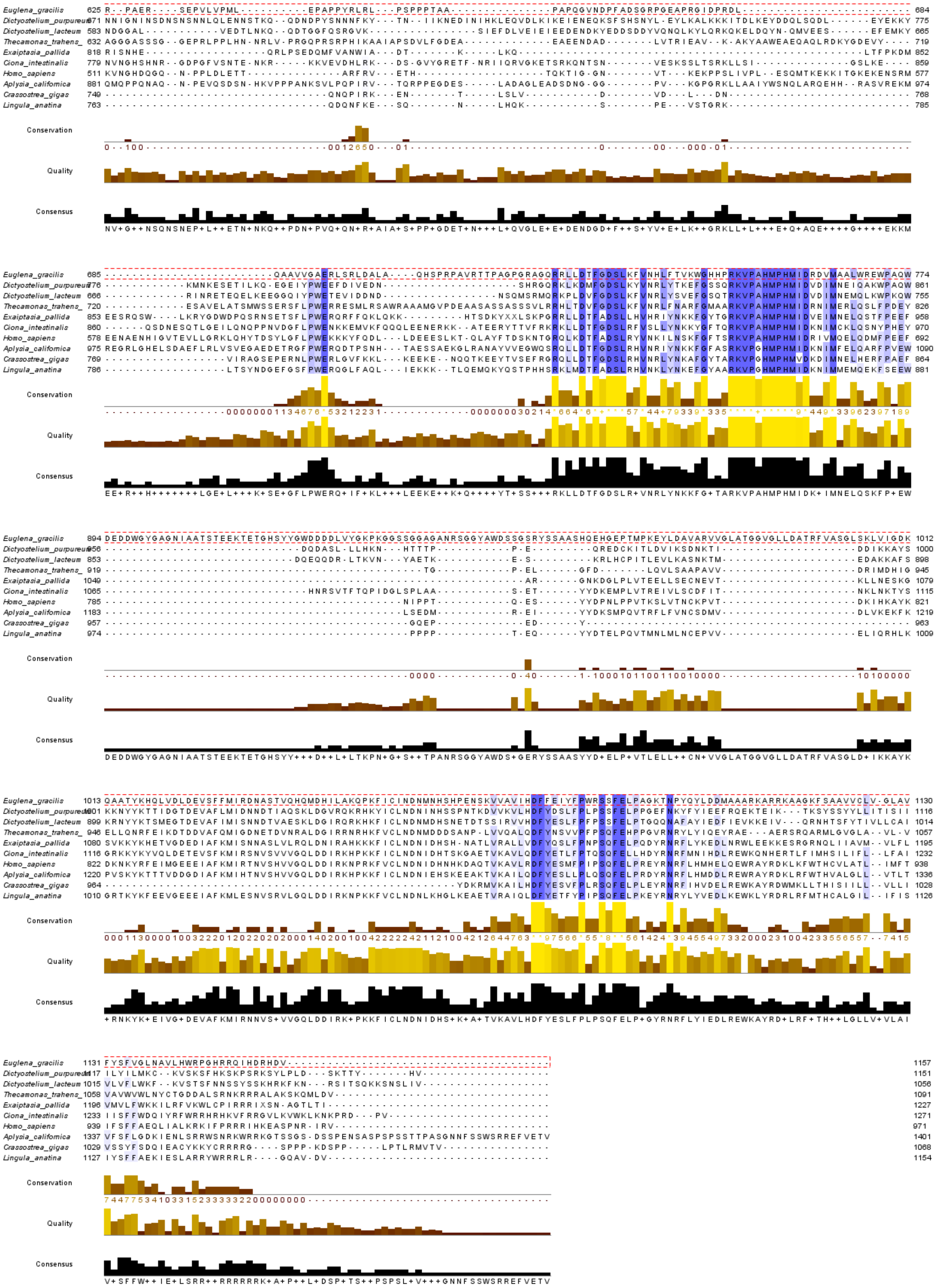
Euglena_gracilis
Dictyostelium_purpureum
Dictyostelium_lacteum
Thecamonas_trahens_
Exaiptasia_pallida
Ciona_intestinalis
Homo_sapiens
Aplysia_californica
Crassostrea_gigas
Lingula_anatina
Conservation
Quality
Consensus

Table Percent identity, similarity, gaps and e value of *Euglena gracilis* *N*-acetylglucosamine-1-phosphotransferase to known protein sequences.

ID	Organism	Identity (%)	Similarity(%)	Gaps (%)	e value
	Euglena gracilis				
XP_003293892.1	*Dictyostelium purpureum*	39	55	8	1e-100
KYQ88805.1	*Dictyostelium lacteum*	37	54	9	1e-98
XP_013758787.1	*Thecamonas trahens*	37	51	12	2e-87
KXJ25731.1	*Exaiptasia pallida*	45	66	3	2e-87
XP_002122528.3	*Ciona intestinalis*	49	64	10	1e-83
EAW97684	*Homo sapiens*	48	64	11	2e-23
XP_005103899.1	*Aplysia californica*	42	57	3	2e-84
EKC24377.1	*Crassostrea gigas*	44	60	3	1e-89
XP_013416777.1	*Lingula anatina*	47	63	2	2e-86
